# Untargeted metabolomics and quantification analysis reveal the shift of chemical constituents between instant dark teas individually liquid-state fermented by *Aspergillus cristatus, Aspergillus niger*, and *Aspergillus tubingensis*

**DOI:** 10.3389/fmicb.2023.1124546

**Published:** 2023-02-09

**Authors:** Si-yu Liao, Yi-qiao Zhao, Wen-bao Jia, Li Niu, Tunyaluk Bouphun, Pin-wu Li, Sheng-xiang Chen, Wei Chen, Dan-dan Tang, Yue-ling Zhao, Yao Zou, Ming-zhi Zhu, Wei Xu

**Affiliations:** ^1^College of Horticulture, Tea Refining and Innovation Key Laboratory of Sichuan Province, Sichuan Agricultural University, Chengdu, China; ^2^Key Laboratory of Tea Science of Ministry of Education, National Research Center of Engineering Technology for Utilization of Functional Ingredients from Botanicals, College of Horticulture, Hunan Agricultural University, Changsha, China; ^3^Faculty of Science and Agricultural Technology, Rajamangala University of Technology Lanna Lampang, Lampang, Thailand

**Keywords:** instant dark teas, fungi, liquid-state fermentation, metabolome, quantification analysis

## Abstract

Instant dark teas (IDTs) were individually liquid-state fermented using the fungi *Aspergillus cristatus, Aspergillus niger*, and *Aspergillus tubingensis*. To understand how the chemical constituents of IDTs were affected by the fungi, samples were collected and measured by liquid chromatography-tandem mass-tandem mass spectrometry (LC-MS/MS). Untargeted metabolomics analysis revealed that 1,380 chemical constituents were identified in positive and negative ion modes, and 858 kinds of chemical components were differential metabolites. Through cluster analysis, IDTs were different from the blank control, and their chemical constituents mostly included carboxylic acids and their derivatives, flavonoids, organooxygen compounds, and fatty acyls. And the metabolites of IDTs fermented by *A. niger* and *A. tubingensis* had a high degree of similarity and were classified into one category, which showed that the fungus used to ferment is critical to the formation of certain qualities of IDTs. The biosynthesis of flavonoids and phenylpropanoid, which involved nine different metabolites such as p-coumarate, p-coumaroyl-CoA, caffeate, ferulate, naringenin, kaempferol, leucocyanidin, cyanidin, and (-)-epicatechin, were significant pathways influencing the quality formation of IDTs. Quantification analysis indicated that the *A. tubingensis* fermented-IDT had the highest content of theaflavin, theabrownin, and caffeine, while the *A. cristatus* fermented-IDT had the lowest content of theabrownin, and caffeine. Overall, the results provided new insights into the relationship between the quality formation of IDTs and the microorganisms used in liquid-state fermentation.

## 1. Introduction

Dark tea is a typical post-fermented tea in China, including Yunnan Pu-erh tea, Hunan Fu-brick tea, Shaanxi Fu-brick tea, Hubei Qing-brick tea, Sichuan Kang-brick tea, and Guangxi Liu-bao-tea (Lin et al., 2021), which is associated with diverse health benefits, such as inhibiting fat deposition, antioxidant, anti-diabetic, anti-cancer, cardiovascular protective, gastrointestinal protective, hepatoprotective, neuroprotective, photoprotective, and sleep regulation (Peng et al., 2014; Lin et al., 2021; Wu et al., 2021; Wei et al., 2022). In pile-fermentation, which is a kind of solid-state fermentation and the key process to forming the unique characteristics of dark tea (Li et al., 2018; Xu et al., 2022b), the microorganism is critical to the quality formation. Chinese dark teas produced *via* different crafts house different microbial communities. *A. cristatus*, which also known as *Eurotium cristatum* (Hubka et al., 2013), is considered the dominant microorganism and plays a key role in the quality formation of Fu-brick tea (Zhu et al., 2020; Wang et al., 2021), contributing to increasing the levels of volatile organic compounds with stale and floral aromas (Xiao et al., 2022). *A. niger* and *A. tubingensis* are the major fungi among Pu-erh tea manufacturers (Abe et al., 2008; Haas et al., 2013; Wang et al., 2015).

Instant dark teas (IDTs), a novel type of tea beverage, are traditionally manufactured using dark tea that has been solid-state fermented, water-extracted, filtered, condensed, and dried (Lu et al., 2015). Additionally, the other way to process instant dark tea is by having microorganisms ferment tea soup, which has the advantages of stability, rapidity, avoiding mixed microbial contamination, and mild manufacturing conditions (Hsu et al., 2002). In previous studies, IDTs were inoculated with *A. cristatus, A. niger*, or *A. tubingensis* for liquid-state fermentation, showing completely different main chemical constituents and sensory qualities (Lu et al., 2015; Wang Q. et al., 2018; Chen et al., 2021; Ma et al., 2021; Wang et al., 2021; Jun et al., 2022). The metabolic pathways, which were critical to the sensory qualities of the IDTs, have unclear relationships with microorganisms.

Metabolomics is an effective tool for explaining the effects of metabolites in tea fermented by microorganisms (Cai et al., 2022). Metabolomics analysis showed that certain microorganisms have important effects on the transformation of metabolites and flavor formation during pile-fermentation (Shi et al., 2021; Li et al., 2022). The previous experiment reveals that *A. cristatus* is critical to the formation of certain qualities of IDT during liquid-state fermentation, using untargeted and targeted metabolomics (An et al., 2021). However, it is unclear how the qualities of IDTs are influenced by the effects of several dominant microorganisms.

In our study, IDTs were fermented in a liquid-state by the fungi (*A. cristatus, A. niger*, and *A. tubingensis*), which were isolated from Fu-brick tea and raw dark tea and identified by phylogenetic analysis. To evaluate the safety of IDTs, ochratoxin and citrinin concentrations were tested using high performance liquid chromatography (HPLC). The content of the main chemical components of IDTs was determined. Metabolites of IDTs were analyzed by ultra-high performance liquid chromatography-hybrid quadrupole time-of-flight/Mass spectrometry (UHPLC-QTOF-MS) and liquid chromatography-tandem mass-tandem mass spectrometry (LC-MS/MS) based untargeted metabolomics. This study has advanced our knowledge regarding how the characteristics of IDTs formed by fermenting in the liquid state.

## 2. Materials and methods

### 2.1. Materials and chemical reagents

Green tea was used as the raw material for liquid-state fermentation and provided by Mabian Wenbin Tea Industry Co., Ltd. (Sichuan, China). Fu-brick tea and raw dark tea were used to isolate the fungal strains provided by Hunan Haoming Tea Industry Food Co., Ltd. (Hunan, China).

Ochratoxin A (OTA), Ochratoxin B (OTB), and Citrinin (CIT) standards were purchased from Pribolab Pte. Ltd. (Qingdao, China). Chromatographic-grade methanol, acetonitrile, and glacial acetic acid were provided by Chengdu Kelong Co., Ltd. (Chengdu, China).

### 2.2. Fungal strains

The strains of Q4, Q54, and ZM1 were isolated from a Fu-brick tea and a raw dark tea and identified using morphological and phylogenetic analysis of fungal internally transcribed spacer (ITS) and partial β-tubulin (*BenA*) gene sequences (Ma et al., 2022), with the identification method as previously reported (Yu et al., 2020). Strains were preserved on potato dextrose agar (PDA) at 4°C. The spore suspensions of the strains were prepared with sterile water and adjusted to 4.0 × 10^6^ CFU/mL for the next liquid-state fermentation.

### 2.3. Tea liquid-state fermentation

Under aerobic conditions, liquid-state fermentation was carried out in a shaking incubator (YS-100B, Changzhou, China). Green tea was infused in distilled water (liquid-to-solid ratio of 60:1 mL/g) and sterilized at 121°C for 20 min. The sterilized tea soup was inoculated with 2% (v/v) spore suspensions and fermented at 25°C with a shaking speed of 190 rpm. Three replicates were set for each treatment, and the blank control was inoculated with the same amount of sterile water. Because of the difference in growth characteristics between the strains, the fermentation duration for *A. cristatus* was 132 h, while the fermentation duration for the other two strains was 72 h. After fermentation ended, the mixtures were, respectively, filtered by multilayer gauze to remove mycelia. The filtrates were centrifuged further at 5,000 rpm for 15 min to obtain instant dark tea (IDTs), which were stored at −80°C in the following experiment.

### 2.4. Determinations of ochratoxins and citrinin

Instant dark teas were filtered by 0.22 μm aqueous filter membrane and then detected the content of ochratoxins and citrinin by HPLC (1260, Agilent, USA). Ochratoxin A (OTA) and ochratoxin B (OTB) were separated using a Phenomenex C_18_ (5 μm, 150 × 4.6 mm) column loaded with water-glacial acetic acid (98:2 v/v) (phase A) and acetonitrile (phase B), with isocratic elution (50:50). Citrinin (CIT) was separated by Phenomenex C_18_ (5 μm, 250 × 4.6 mm) column, using the mobile phase of acetonitrile-isopropanol-phosphoric acid (35:10:55 v/v).

### 2.5. Determination of main chemical compounds

The contents of tea polyphenols, tea pigments (theaflavins, thearubigins, and theabrownin), and amino acids were determined using spectrophotometry, as previously described (Wang et al., 2011, 2014). The content of caffeine was determined using ultraviolet spectrophotometry in GB/T 8312-2013, and the content of organic acids were determined using acid-base indicator titration in GB/T 12456-2021.

### 2.6. Metabolomics

#### 2.6.1. Metabolites extraction

The IDTs samples were thawed at 4°C. Then 100.0 μL of samples fermented by different fungal strains were individually extracted with 300 μL of methanol, 20 μL of internal standard substances were added, vortexed for 30 s, ultrasonically treated for 10 min (incubated in ice water), and incubated for 1 h at −20°C to precipitate proteins. The sample was then centrifuged for 15 min at 4°C, 13,000 rpm. Transfer the supernatant (200 μL) into a 2 mL LC/MS glass vial, take 20 μL from each sample and pool them as QC samples, and take 200 μL supernatant for UHPLC-QTOF-MS analysis.

#### 2.6.2. LC-MS/MS analysis

LC-MS/MS analysis was performed using an HPLC system (1290, Agilent Technologies) with a UPLC BEH Amide column (1.7 μm, 2.1 × 100 mm) coupled to a Triple TOF 5600 (Q-TOF, AB Sciex) at a flow rate of 0.5 mL⋅min^–1^. Mobile phase A consisted of 25 mM NH_4_OAc and 25 mM NH_4_OH in water (pH = 9.75), and mobile phase B was composed of acetonitrile. The gradient program was as follows: 0 min, 95% B; 7 min, 65% B; 9 min, 40% B; 9.1 min, 95% B; 12 min, 95% B. The injection volume was 3 μL. The Triple TOF mass spectrometer was used for its ability to acquire MS/MS experiments. In this mode, the acquisition software (Analyst TF 1.7, AB Sciex) continuously evaluated the full scan survey MS data as it collected and triggered the acquisition of MS/MS spectra depending on preselected criteria. In each cycle, 12 precursor ions whose intensities were greater than 100 were chosen for fragmentation at collision energy (CE) of 30 V (15 MS/MS events with product ion accumulation time of 50 ms each). ESI source conditions were set as follows: Ion source gas 1 at 60 Psi, Ion Spray Voltage Floating (ISVF) 5,000 or −4,000 V in positive or negative modes, respectively.

#### 2.6.3. Data preprocessing and annotation

MS raw data (.d) files were converted to the mzXML format using ProteoWizard and processed by R package XCMS (version 3.2). The preprocessing results generated a data matrix that consisted of the retention time (RT), mass-to-charge ratio (m/z) values, and peak intensity. The R package CAMERA was used for peak annotation after XCMS data processing. An in-house MS2 database was applied to the identification of metabolites.

### 2.7. Statistical analysis

All tests were repeated triple times, and data were expressed as means with standard deviation (SD). Duncan’s one-way ANOVA (for ≥ 3 samples) was carried out using the SPSS 27.0 software (SPSS Inc. Chicago, IL, USA). Differences were considered statistically significant when the *p*-value was less than 0.05. The principal component analysis (PCA), heat map, different metabolite screening (fold change ≥ 2.0, *P* value ≤ 0.05, and variable importance in projection value ≥ 1.0), functional annotation, and enrichment analysis of differential metabolite KEGG were performed using BMK Cloud^1^. Based on the metabolism classification information provided by the HMDB database^2^, the annotated differential metabolism in IDTs samples is statistically mapped. The clusterProfiler, an R packager specially used for enrichment analysis of GO and KEGG, was used to enrich and analyze the annotation results of the differential metabolite KEGG^3^ by using the hypergeometric test method to draw an enrichment network diagram.

## 3. Results and discussion

### 3.1. Strains

**FIGURE 1:**
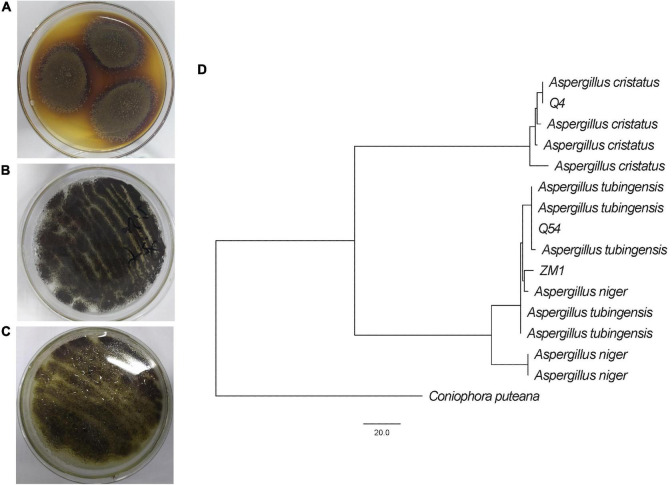
Identification of *A. cristatus* (Q4) **(A)**, *A. tubingensis* (Q54) **(B)**, and *A. niger* (ZM1) **(C)**, including colony morphology on PDA plates and molecular phylogenetic analysis **(D)**.

When cultured on PDA plates, the mycelia of strains Q4, Q54, and ZM1 were golden, white, and white, respectively, and the conidial areas were dark blonde, black, and dark green, respectively. Through the colony morphology and phylogenetic analysis, strains Q4, Q54, and ZM1 were identified as *A. cristatus* (the ITS and partial *BenA* gene sequences were deposited in GenBank under accession numbers OQ135132 and OQ136613, respectively), *A. niger* (the ITS and partial *BenA* gene sequences were deposited in GenBank under accession numbers OQ121833 and OQ136614, respectively), and *A. tubingensis* (the ITS and partial *BenA* gene sequences were deposited in GenBank under accession numbers OQ121834 and OQ136615, respectively) stains, respectively (Figure 1). Considering the safety of IDTs fermented by the strains (Blanc et al., 1995; O’Brien et al., 2005; Malir et al., 2016), the mycotoxins (OTA, OTB, and CIT) were tested by HPLC and results shown that were below limit of detection (Supplementary Table 1).

### 3.2. Changes in chemical components in IDTs

The composition and contents of chemical components determine the sensory of IDTs. In this study, the contents of chemical components in IDTs samples fermented by *A. niger* (IDTB), *A. cristatus* (IDTC), and *A. tubingensis* (IDTD) were listed in Table 1. The contents of tea polyphenols (tea polyphenols, TPs), amino acids (amino acids, AA), and organic acids (organic acids, OA) were reduced under the function of the microorganisms.

**TABLE 1:** Chemical constituents of instant dark teas fermented in liquid-state by *A. cristatus, A. niger*, and *A. tubingensis*.

Chemical constituents (mg/mL)	Instant dark teas samples			
	IDTA	IDTB	IDTC	IDTD
Tea polyphenols	2.050 ± 0.191^A^	0.928 ± 0.144^BC^	0.877 ± 0.445^C^	1.184 ± 0.199^B^
Amino acids	0.473 ± 0.104^A^	0.109 ± 0.186^D^	0.177 ± 0.114^C^	0.204 ± 0.085^B^
Caffeine	0.028 ± 0.001^B^	0.029 ± 0.001^B^	0.016 ± 0.001^C^	0.031 ± 0.001^A^
Theaflavin	0.029 ± 0.021^AB^	0.0048 ± 0.002^C^	0.001 ± 0.006^BC^	0.038 ± 0.001^A^
Thearubigins	0.655 ± 0.080^A^	0.583 ± 0.159^A^	0.092 ± 0.0021^B^	0.384 ± 0.450^AB^
Theabrownins	0.464 ± 0.021^B^	0.388 ± 0.060^BC^	0.309 ± 0.020^C^	0.651 ± 0.087^A^
Tea pigments	1.148 ± 0.041^A^	0.934 ± 0.223^A^	0.410 ± 0.014^B^	0.658 ± 0.181^B^
Malic acid	26.353 ± 8.595^A^	5.243 ± 1.355^B^	23.450 ± 2.978^A^	10.832 ± 3.582^B^
Acetic acid	23.600 ± 7.697^A^	4.567 ± 1.266^B^	21.000 ± 2.666^A^	9.700 ± 3.207^B^
Tartaric acid	29.500 ± 9.622^A^	5.733 ± 1.570^B^	26.250 ± 3.333^A^	12.125 ± 4.010^B^
Citric acid	25.173 ± 8.211^A^	4.780 ± 1.409^B^	22.400 ± 2.844^A^	10.347 ± 3.422^B^
Citric acid monohydrate	27.533 ± 8.980^A^	5.267 ± 1.514^B^	24.500 ± 3.111^A^	11.317 ± 3.742^B^
Lactic acid	35.400 ± 11.546^A^	7.267 ± 1.804^B^	31.500 ± 4.000^A^	14.550 ± 4.812^B^
Total organic acids	220.267 ± 71.842^A^	43.200 ± 11.538^B^	196.000 ± 24.887^A^	90.533 ± 29.939^B^

The different fermentation treatments of instant dark tea were the blank control (IDTA), *Aspergillus niger* (IDTB), *Aspergillus cristatus* (IDTC), and *Aspergillus tubingensis* (IDTD). Data are shown as mean values ± standard deviation. The letters A, B, C (superscript) represent the degree of significant difference in data. The values marked with different uppercase letters within a given row were significantly different (*p* < 0.05).

TPs are the main water-soluble substances in tea (Senanayake, 2013) and the sources of tea soup convergence, whose possible consumption ways were as follows: one part provided energy for the survival of microorganisms, while the other part was converted into their oxidation products under the action of microorganisms. During fermentation, enzymes secreted by microorganisms converted TPs into quinones first, then further oxidized and polymerized to form tea pigments (Wang Q. et al., 2018). Tea pigments, including theaflavins (theaflavins, TFs), thearubigins (thearubigins, TRs), and theabrownins (theabrownins, TBs), are the reason for color changes in tea soup after fermentation and have the characteristics of anti-atherosclerosis, anti-obesity, and maintain muscle health (Gong et al., 2010; Liu et al., 2016; Xu et al., 2022a). Compared to IDT prepared by blank control (IDTA), IDTB had the highest content of TRs, IDTD had the highest content of TPs, TFs, and TBs, while IDTC had the lowest content of TPs, TFs, and TRs (*p* < 0.05). These data indicated that *A. tubingensis* could efficiently utilize TPs and catalyze the transformation from TPs to TFs and TBs, while *A. cristatus* had a weak ability to catalyze the oxidation of TPs but consumed the highest contents of TPs in IDTs fermented by fungi. Our study showed that the mellow mouthfeel of IDTs would better format with the degradation of TPs and the formation of tea pigments under the action of microorganisms (Wang Y. et al., 2018).

The purine alkaloids in teas typically include caffeine, theobromine, and theophylline, and caffeine contributes to tea’s stimulant properties (Wang Q. et al., 2018). The caffeine content of IDTD was significantly higher than the other IDTs (*p* < 0.05), considering that certain secretions from *A. tubingensis* can promote the production of caffeine or the inability of *A. tubingensis* in liquid-state fermentation to use the caffeine originally present in tea soup. The high content of caffeine in IDTD should allow it to be a better stimulant than the other IDTs. Instead, the caffeine content of IDTC was lowest in the IDTs (*p* < 0.05), which indicated that the caffeine originally present in tea soup could be significantly consumed or degraded through liquid-state fermentation by *A. cristatus*. Therefore, IDTC had a more mellow mouthfeel in the IDTs and *A. cristatus* had the potential to develop tea beverages with low content of caffeine. A recent study showed that the content of caffeine increased during the liquid-state fermentation by *A. cristatus* (An et al., 2021), which is different from our results. This may be caused by the difference in microorganisms. Additionally, there was no significant difference in caffeine content between IDTA and IDTB (*p* < 0.05), showing that *A. niger* had no significant effect on caffeine metabolism. This indicated the fungal species (*A. niger, A. tubingensis*, and *A. cristatus*) are responsible for caffeine metabolism, which can affect the caffeine content through different microbial pathways.

AA can help to form the mellow taste of tea (An et al., 2021). The level of AA in IDTs fermented by fungi was significantly lower than the corresponding level in IDTA (*p* < 0.05). In IDTs fermented by fungi, the level of AA in IDTB was the lowest but it in IDTD was the highest (*p* < 0.05), which showed that *A. niger* could significantly consume AA originally, but *A. tubingensis* had the weakest ability to consume AA in the strains used to produce IDTs. This may be because AA, one of the readily metabolize nutrients in tea soup (Wang Q. et al., 2018), were abundantly used during fungal growth.

OA were key constituents in tea leaves that influence the tea quality (Xu et al., 2013). In this study, the contents of OA in IDTs were determined, including malic acid, tartaric acid, acetic acid, citric acid, citric acid monohydrate, and lactic acid. The levels of OA in IDTB and IDTD were higher than the corresponding levels in IDTA and IDTC, and there were no significant differences in the organic levels between IDTA and IDTC. This indicated that *A. cristatus* could cause a better biological activity of IDT than *A. niger* and *A. tubingensis*.

### 3.3. Metabolomics analysis of IDTs

#### 3.3.1. PCA analysis and vene diagram

**FIGURE 2:**
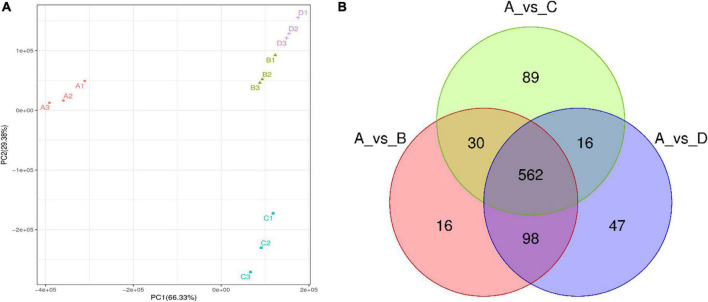
The metabolomics data were subjected to principal component analysis (PCA) **(A)** and Vene diagram **(B)** of differential metabolites in IDTs. The different fermentation treatments of instant dark tea were the blank control [A], *Aspergillus niger* [B], *Aspergillus cristatus* [C], and *Aspergillus tubingensis* [D].

Through non-target metabolomics technology, a total of 1,380 chemicals were detected in the IDTs (A refers to the IDT was produced by the blank control; B refers to IDT was fermented by *A. niger*; C refers to IDT was fermented by *A. cristatus*; D refers to IDT was fermented by *A. tubingensis*) in positive and negative modes (Supplementary Table 2), including 858 metabolites which closely related to the quality formation of IDTs (Figure 2B). The PCA differentiated the four IDTs samples (Figure 2A), indicating that there were significant differences between the IDTs fermented by the strains and the blank control. The principal components of IDTB and IDTD were similar, which were quite different from the principal components of IDTC, for the close biological relationship between *A. niger* and *A. tubingensis* (Juhász et al., 2004).

The compounds with FC value greater than 2.0, *P* value less than 0.05, and VIP value greater than 1.0 were defined as differential metabolites among IDTs samples. Compared with the blank control (Figure 2B), 858 metabolites were clustered after trimming and filtering, and 562 core metabolites were shared by the IDTs fermented by different strains. Additionally, compared with the IDTB and IDTD, the total number of metabolites in IDTC was the minimum, but the number of exclusive metabolites in IDTC was the maximum. There is the least number of similar metabolites between the IDTC and IDTD. This implied that the microorganisms used for liquid-state fermentation are critical to the quality formation of IDTs.

#### 3.3.2. Heatmap and histogram of differential metabolites

**FIGURE 3:**
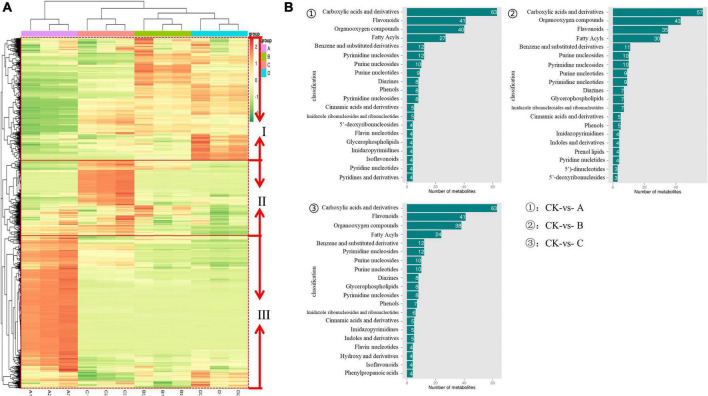
The metabolomics data were subjected to heatmap analysis **(A)** and column diagram differential metabolites **(B)** in IDTs. In the heatmap, a color-coded scale grading from orange to green represented the metabolites from high to low. The different fermentation treatments of instant dark tea were the blank control [A], *Aspergillus niger* [B], *Aspergillus cristatus* [C], and *Aspergillus tubingensis* [D].

Heatmap was used to visualize the differences of metabolites among IDTs fermented by dissimilar strains, in which each column represents an IDT sample and each row represents a metabolite (Figure 3A). *Via* thermogram analysis, the metabolites polymerization in IDTs is divided into three categories. Compared with the blank control, the expression of categories I substance was significantly upregulated in IDTB and IDTD, while the expression of categories II substance was only upregulated significantly in IDTC. For the category III substance, it was significantly downregulated in IDTs fermented by the three stains, indicating that there was consistency in the transformation of the metabolites of IDTs under the function of the three fungi strains. According to the similarity in metabolite classification, the composition of metabolites of IDTB and IDTD, confirms our previous conclusion. Therefore, in the heatmap diagram, IDTB and IDTD were classified into one category, and IDTC belonged to one group with them for the metabolites of it were significantly different from IDT produced by the blank control.

Based on the metabolites classification information provided by the HMDB databases, the annotated differential metabolism in IDTs is statistically mapped (Figure 3B), and the number of substances species was more than 20 were considered as the key metabolites. The diagram showed that the key metabolites in IDTs production were carboxylic acids and their derivatives, flavonoids, organooxygen compounds, and fatty acyls. It was different for the order of the number of these key metabolites in IDTs fermented by dissimilar strains. And the key metabolites numbers in IDTC showed more differences than it in IDTB and IDTD, which had the largest number of carboxylic acids and their derivatives and the smallest number of fatty acids. Besides, for the other two key metabolites, the number of flavonoids in IDTC was smaller than it in the other two IDTs fermented by fungi. This indicated that flavonoids and carboxylic acids and their derivatives were more used by *A. cristatus* in the instant dark tea production process. This may be due to the relatively low utilization of organooxygen compounds and fatty acyls by *A. cristatus*, or *A. cristatus* can promote the synthesis of these two metabolites, which needs further experimental verification.

#### 3.3.3. Analysis of enrichment network and metabolic pathway of metabolites

**FIGURE 4:**
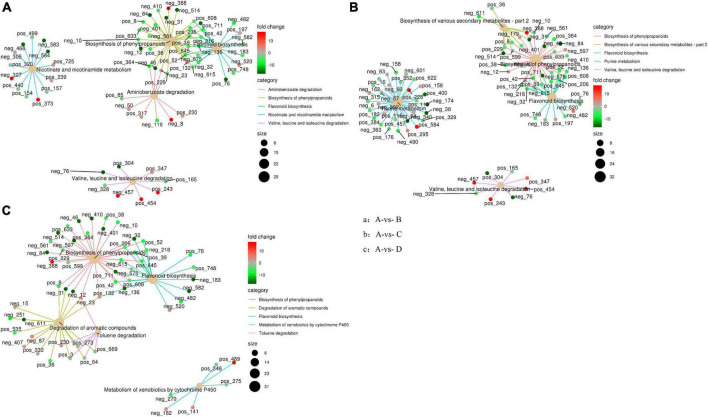
Enrichment network diagram of differential metabolites in IDTs. Pictures respectively represent metabolites pathways between IDT produced by blsnk control and IDTs fermented by fungi. The light-yellow node refers to the associated metabolic pathway, and its size of it indicates the number of metabolites included by these pathways. The dots connected with the node refer to the specific metabolites annotated to the pathway, along with the color depth indicating the difference multiple taking the value of log 2. The different fermentation treatments of instant dark tea were the blank control [A], *Aspergillus niger* [B], *Aspergillus cristatus* [C], and *Aspergillus tubingensis* [D].

The enrichment network of metabolites in IDTs is drawn in Figure 4, along with heat dots representing the levels of associated metabolites. The results showed the main metabolic pathway in IDTs fermented by the strains. The main pathways in IDTs were as follows: IDTB involved aminobenzoate degradation, biosynthesis of phenylpropanoids, flavonoid biosynthesis, nicotinamide metabolism, and valine, leucine and isoleucine degradation; IDTC involved biosynthesis of phenylpropanoids, biosynthesis of various secondary metabolites metabolites-part 2, flavonoid biosynthesis, purine metabolism, and valine, leucine and isoleucine degradation; IDTD involved biosynthesis of phenylpropanoids, degradation of aromatic compounds, flavonoid biosynthesis, metabolism of xenobiotics by cytochrome P450, and toluene degradation.

In this study, the biosynthesis of flavonoids and phenylpropanoids was the mutual metabolic pathway in IDTs fermented by the three stains, including the largest number of metabolites for their biggest size of the node. The metabolites in flavonoid biosynthesis were a comprehensive decline under the function of the strains, indicating that the flavonoid substances in tea soup were transformed and utilized with the influence of microorganisms. IDTs fermented by microorganisms can obtain a mild taste because flavonoid is one of the sources of the astringent sense of tea soup. Flavonoid is also one of the main components of tea polyphenols (He et al., 2020), so it may be the central carbon source for microbial growth provided by the tea soup. Besides, expect the synthesis of chorismite, the metabolites of phenylpropanoids biosynthesis have significantly risen under the function of the strains. Phenylalanine is the precursor of phenylpropanoid biosynthesis and is eventually converted into flavanols and flavonol glycosisdes through the flavonoid biosynthesis pathway after catalysis by a series of enzymes (Wu et al., 2020; Sun et al., 2022). In our previous study, we certified that amino acid contents were significantly reduced under the action of microorganisms. This indicated that the transformation from phenylpropanoids to phenylpropanoid was promoted and the biosynthesis pathway of flavonoid from phenylpropanoid was inhibited, with the effects of microbial extracellular enzymes in the liquid-state fermentation system.

The degradation of valine, leucine, and isoleucine was a collective metabolic pathway in IDTs fermented by *A. niger* and *A. cristatus*, with the metabolites of (s)-3-hydroxyisobutyl-CoA, and (s)-b-aminoisobutyric acid significantly rose and the metabolites of methylcrotonyl-CoA slightly rose. For IDTD, the metabolism of caffeic acid was significantly decreased in the degradation of its aromatic compounds of the IDT. Caffeic acid is a phenolic compound, with the activity of antioxidant, anti-inflammatory, and anti-carcinogenic (Espíndola et al., 2019). Therefore, IDTD may have certain healthcare functions.

**FIGURE 5:**
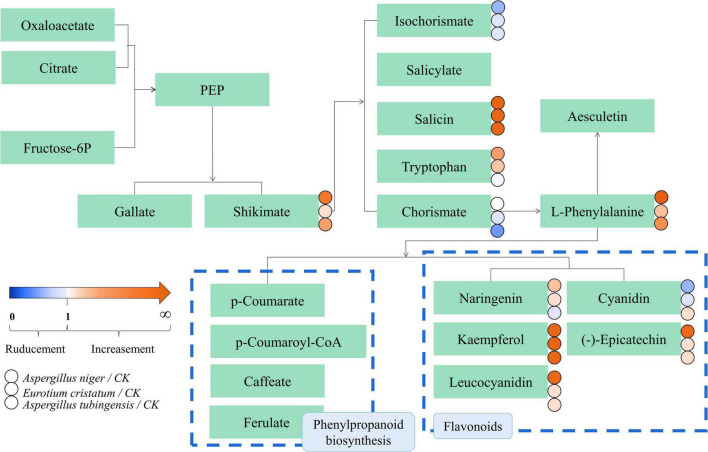
metabolic pathway diagram of the differential metabolites in IDTs. The dots represent the relative content of metabolites between the IDT fermented by microorganisms and the bank control. The dots represent the relative content of metabolites between IDT produced by blank control and IDTs fermented by fungi. The color-coded scale grading from orange to blue represented the metabolites from high to low. The different fermentation treatments of instant dark tea were the blank control [A], *Aspergillus niger* [B], *Aspergillus cristatus* [C], and *Aspergillus tubingensis* [D].

The possible metabolic pathways for the quality formation of IDTs fermented by microorganisms were drawn in Figure 5, along with color-coded dots representing the levels of the associated metabolites. There were 22 substances differentially expressed in the metabolic transformation pathway shown in Figure 5. In the diagram, the biosynthesis of phenylpropanoid and flavonoids were also the main metabolic pathway influencing the quality formation of IDTs, like our previous results. There were nine differential metabolites in the pathways: p-coumarate, p-coumaroyl-CoA, caffeate, ferulate, naringenin, kaempferol, leucocyanidin, cyanidin, and (-)-epicatechin, which may be driven by extracellular enzymes secreted by microorganisms.

As mentioned above, microorganisms are critical to the qualities of IDTs in liquid-state fermentation, for their function in the transformation of water-soluble substances in tea soup. In our study, the transformation and utilization of flavonoids and phenylpropanoid have high consistency under the function of *A. niger, A. cristatus*, and *A. tubingensis* in the process of liquid-state fermentation, which indicated that single fungus fermentation is conducive to targeted changes in the content of certain substances and developing functional tea products or guiding the production of instant dark tea products according to market demand.

## 4. Conclusion

This study explored the relationship between the quality formation of instant dark teas (IDTs) and the dominant microorganisms (*A. niger, A. cristatus*, and *A. tubingensis*) used for liquid-state fermentation, and revealed the main metabolic pathways during fermentation. The fermentation of IDTs by the dominant microorganisms caused the degradation of tea polyphenols, amino acids, and organic acids. Especially, the IDT fermented by *A. tubingensis* had the highest content of theaflavins, theabrownins, and caffeine, while the IDT fermented by *A. cristatus* had the lowest content of theabrownins and caffeine. Metabolic results revealed that 22 metabolites were identified as key metabolites responsible for differential metabolic pathways among IDTs fermented by strains. And the biosynthesis of flavonoids and phenylpropanoids were the key metabolic pathways in the quality formation of IDTs, with nine differential metabolites including p-coumarate, p-coumaroyl-CoA, caffeate, ferulate, naringenin, kaempferol, leucocyanidin, cyanidin, and (-)-epicatechin. Our results advance our insights into the metabolic changes in the quality formation of IDTs processed by liquid-state fermentation using *A. niger, A. cristatus*, and *A. tubingensis*.

## Data availability statement

The data presented in our study are deposited in the https://www.ncbi.nlm.nih.gov/ repository, accession numbers: OQ135132, OQ121833, OQ121834, OQ136613, OQ136614, and OQ136615.

## Author contributions

S-YL and Y-QZ participated in conceptualization, methodology, software, formal analysis, investigation, data curation, and visualization. W-BJ participated in software and investigation. TB participated in adding and editing and reviewing all parts of the manuscript. P-WL and S-XC participated in resources and validation. LN, WC, D-DT, Y-LZ, and YZ participated in conceptualization and methodology. M-ZZ participated in review and editing. WX participated in conceptualization, writing—review and editing, and funding acquisition. All authors contributed to the article and approved the submitted version.

## Footnotes

1
https://www.biocloud.net

2
https://hmdb.ca/

3
https://www.genome.jp/kegg
