# Supplementary material for: Untargeted metabolomics and quantification analysis reveal the shift of chemical constituents between instant dark teas individually liquid-state fermented by Aspergillus cristatus, Aspergillus niger, and Aspergillus tubingensis
Source: Front Microbiol. 2023 Feb 9;14:1124546. doi: 10.3389/fmicb.2023.1124546 (PMC9947791; doi:10.3389/fmicb.2023.1124546)
Supplement: Supplementary file 1 [file Table_1.DOCX]

**Supplementary material**

**Untargeted metabolomics and quantification analysis reveal the shift of chemical constituents between instant dark teas individually liquid-state fermented by *Aspergillus cristatus*, *Aspergillus* *niger*, and *Aspergillus* *tubingensis***

Si-yu Liao^1^, Yi-qiao Zhao^1^, Wen-bao Jia^1^, Li Niu^2^, Bouphun Tunyaluk^3^, Pin-wu Li^1^, Sheng-xiang Chen^1^, Wei Chen^1^, Dan-dan Tang^1^, Yue-ling Zhao^1^, Yao Zou^1,*^, Ming-zhi Zhu^2,*^, Wei Xu^1,*^

1 College of Horticulture, Tea Refining and Innovation Key Laboratory of Sichuan Province, Sichuan Agricultural University, Chengdu 611130, China

2 Key Laboratory of Tea Science of Ministry of Education, National Research Center of Engineering Technology for Utilization of Functional Ingredients from Botanicals, College of Horticulture, Hunan Agricultural University, Changsha, 410128, China

3 Faculty of Science and Agricultural Technology, Rajamangala University of Technology Lanna Lampang, Lampang, 52000, Thailand

* Corresponding authors:

E-mail addresses: zouyao82@163.com (Yao Zou), mzzhucn@hotmail.com (Ming-zhi Zhu), xuweianti@sicau.edu.cn (Wei Xu).

Table captions

Table S1 Contents of three kinds of standard mycotoxin (μg/kg) in IDTs fermented by fungal strains

| Kinds of mycotoxins | Fungal strains used to produce instant dark tea | | |
| --- | --- | --- | --- |
|  | *A. cristatus* | *A*. *niger* | *A.* *tubingensis* |
| OTA | <LOD | <LOD | <LOD |
| OTB | <LOD | <LOD | <LOD |
| CIT | <LOD | <LOD | <LOD |

Table S2 Tentative identification of 1380 metabolites in IDTs

| NO. | ID | name | Instant dark tea samples | | | | | | | | | | | |
| --- | --- | --- | --- | --- | --- | --- | --- | --- | --- | --- | --- | --- | --- | --- |
|  |  |  | A1 | A2 | A3 | B1 | B2 | B3 | C1 | C2 | C3 | D1 | D2 | D3 |
| 1 | neg_1 | D-Malic acid | 7493.393 | 7869.36 | 8348.29 | 2694.358 | 2895.617 | 2897.693 | 1790.094 | 1923.708 | 1996.874 | 2242.535 | 2434.567 | 2536.903 |
| 2 | neg_2 | 2,3-Dihydroxybenzoic acid | 16.80318 | 18.05293 | 18.13106 | 266.4228 | 266.8683 | 291.2756 | 317.4138 | 340.7522 | 363.2271 | 1894.065 | 2027.895 | 2060.631 |
| 3 | neg_3 | Salicylic acid | 716.2911 | 762.4315 | 822.9522 | 467.6505 | 517.4331 | 539.8547 | 572.3487 | 580.7289 | 611.0972 | 596.4633 | 624.8128 | 634.1984 |
| 4 | neg_4 | L-Norvaline | 4037.128 | 4112.666 | 4248.296 | 4652.678 | 4940.555 | 5027.333 | 4484.224 | 4970.89 | 5226.165 | 4354.358 | 4738.031 | 5124.552 |
| 5 | neg_5 | L-Arginine monohydrochloride | 80.39559 | 87.02777 | 94.9617 | 0 | 0 | 0 | 59.80775 | 61.79129 | 62.38591 | 0 | 0 | 0 |
| 6 | neg_6 | Guanosine | 199.9471 | 212.5744 | 212.7333 | 14.65214 | 14.80214 | 15.29404 | 14.4825 | 14.49749 | 15.01674 | 67.52204 | 70.78482 | 74.23667 |
| 7 | neg_7 | Homovanillic acid | 8.803992 | 9.173088 | 9.80323 | 29.89381 | 31.28666 | 33.14264 | 11.09738 | 11.87741 | 12.54923 | 35.70977 | 37.18968 | 40.64996 |
| 8 | neg_8 | Hydroquinone | 0 | 0 | 0 | 10.6686 | 10.85737 | 11.48835 | 10.00562 | 10.99096 | 11.30213 | 66.62741 | 68.74022 | 72.9733 |
| 9 | neg_9 | Pantothenate | 390.3574 | 429.1173 | 450.7646 | 598.3143 | 653.1571 | 659.9158 | 500.4791 | 552.3277 | 593.8321 | 658.9659 | 700.2667 | 740.8649 |
| 10 | neg_10 | L-Phenylalanine | 3904.834 | 4075.583 | 4326.064 | 107.7763 | 109.1896 | 116.1719 | 262.5451 | 272.6829 | 278.6864 | 24.10089 | 25.6263 | 26.94029 |
| 11 | neg_11 | 2-Chloro-L-phenylalanine | 52318.39 | 52667.54 | 52712.88 | 50885.58 | 51363.93 | 55350.83 | 49113.02 | 49352.21 | 50208.42 | 46611.88 | 49993.65 | 51709.32 |
| 12 | neg_12 | Caffeic acid | 134.0594 | 143.863 | 145.3583 | 0 | 0 | 0 | 286.3713 | 302.0658 | 307.5659 | 0 | 0 | 0 |
| 13 | neg_13 | Uridine 5'-monophosphate | 12402.87 | 13758.7 | 14621.46 | 10375.71 | 11161.73 | 11874.55 | 9691.246 | 10010.56 | 10234.91 | 10462.88 | 11363.45 | 11800.94 |
| 14 | neg_14 | 3,4-Dihydroxybenzoic acid | 567.6331 | 567.9867 | 586.6481 | 128.8964 | 142.2792 | 143.2314 | 112.9444 | 119.1077 | 123.0543 | 97.77572 | 105.4017 | 115.5285 |
| 15 | neg_15 | trans-3-Coumaric acid | 190.6023 | 201.3198 | 207.662 | 688.6269 | 722.526 | 732.3164 | 1239.111 | 1244.92 | 1317.26 | 1517.269 | 1669.043 | 1793.505 |
| 16 | neg_16 | L-Glutamate | 1036.188 | 1079.101 | 1092.922 | 44.06856 | 45.30889 | 46.72523 | 542.1027 | 557.6252 | 576.8543 | 31.78498 | 32.71954 | 34.65815 |
| 17 | neg_17 | D-Guanosine | 176.5998 | 190.6152 | 197.1244 | 27.42664 | 29.72309 | 32.16069 | 19.69574 | 21.04164 | 22.23459 | 95.49129 | 103.3829 | 109.9748 |
| 18 | neg_18 | Theophylline | 17.07285 | 18.60031 | 18.60365 | 38.55594 | 41.80751 | 44.17071 | 457.8066 | 477.1888 | 489.8694 | 55.13492 | 55.2577 | 60.16198 |
| 19 | neg_19 | Uridine 5'-monophosphate (UMP) | 236.533 | 258.2348 | 277.3157 | 117.0833 | 122.1347 | 128.3996 | 77.19033 | 81.70568 | 82.75333 | 107.7862 | 116.9411 | 125.3307 |
| 20 | neg_20 | D-Fructose 6-phosphate disodium salt hydrate | 2724.198 | 2795.972 | 2958.136 | 3455.254 | 3713.586 | 4074.105 | 3129.975 | 3161.553 | 3394.144 | 3232.667 | 3393.517 | 3584.985 |
| 21 | neg_21 | Gentisic acid | 1.187778 | 1.273812 | 1.394069 | 7.162256 | 7.721667 | 7.843534 | 47.53768 | 48.57151 | 52.55233 | 0 | 0 | 0 |
| 22 | neg_22 | 3.alpha.-Mannobiose | 158.0332 | 170.8455 | 184.131 | 7.571608 | 8.262235 | 8.364015 | 371.0997 | 403.9705 | 443.9205 | 7.348892 | 8.132118 | 8.471732 |
| 23 | neg_23 | 4-Hydroxybenzoic acid | 80.10306 | 85.07069 | 87.99193 | 499.0483 | 525.6051 | 555.3553 | 128.9625 | 129.2375 | 136.1918 | 2275.431 | 2309.079 | 2320.703 |
| 24 | neg_24 | Adenosine 5'-monophosphate disodium salt | 109.7138 | 118.8664 | 125.1577 | 6.318656 | 6.876223 | 6.931209 | 0.015461 | 0.015531 | 0.01604 | 3.982049 | 4.395464 | 4.784298 |

Continued Table S2

| 25 | neg_25 | cis-9-Palmitoleic acid | 1.774281 | 1.813557 | 1.8392 | 3.429252 | 3.679194 | 3.831787 | 41.25179 | 45.61576 | 48.6253 | 2.940294 | 3.018214 | 3.092377 |
| --- | --- | --- | --- | --- | --- | --- | --- | --- | --- | --- | --- | --- | --- | --- |
| 26 | neg_26 | Sodium gluconate | 3780.41 | 3855.16 | 4031.63 | 117.9603 | 127.908 | 135.738 | 850.5877 | 864.6309 | 879.2147 | 350.5711 | 375.3081 | 392.1067 |
| 27 | neg_27 | L-Fucose | 3014.412 | 3155.068 | 3259.532 | 2733.269 | 2767.266 | 3040.514 | 2729.792 | 2890.608 | 2991.387 | 3039.094 | 3365.43 | 3685.518 |
| 28 | neg_28 | D-(+)-Raffinose pentahydrate | 1797.869 | 1831.689 | 1973.364 | 73.81672 | 74.32892 | 76.13614 | 43.17955 | 45.05349 | 45.14609 | 55.97038 | 58.05494 | 61.54394 |
| 29 | neg_29 | D-Tryptophan | 2889.445 | 3029.693 | 3190.84 | 1038.222 | 1131.814 | 1191.556 | 768.5269 | 816.3454 | 865.9968 | 880.9304 | 931.348 | 948.5228 |
| 30 | neg_30 | UDP-N-acetylglucosamine | 2202.739 | 2423.669 | 2457.556 | 4307.323 | 4418.577 | 4703.665 | 4063.31 | 4114.715 | 4377.111 | 5323.896 | 5879.326 | 6376.184 |
| 31 | neg_31 | 4-Hydroxycinnamic acid | 1236.954 | 1360.087 | 1403.417 | 0 | 0 | 0 | 0 | 0 | 0 | 0 | 0 | 0 |
| 32 | neg_32 | Epigallocatechin | 1067.12 | 1122.499 | 1179.863 | 6.648521 | 6.701943 | 7.122128 | 32.62821 | 32.79461 | 35.76456 | 0 | 0 | 0 |
| 33 | neg_33 | Oxidized glutathione | 558.6197 | 587.8312 | 607.2628 | 531.3041 | 563.375 | 613.9215 | 358.5095 | 375.2159 | 406.0583 | 447.0866 | 494.4143 | 508.2861 |
| 34 | neg_34 | 2-aceto-2-hydroxy-butyrate | 18848.87 | 20265.42 | 21427.83 | 0 | 0 | 0 | 4.031081 | 4.146165 | 4.45407 | 0 | 0 | 0 |
| 35 | neg_35 | 2'-Deoxyinosine | 118.9406 | 121.0023 | 123.8069 | 124.588 | 138.0651 | 140.5767 | 120.916 | 130.1637 | 141.7112 | 139.125 | 146.2227 | 157.6478 |
| 36 | neg_36 | Uridine | 108.0402 | 109.4516 | 112.1491 | 0 | 0 | 0 | 3.848867 | 3.886298 | 4.260545 | 0.974998 | 1.038647 | 1.128688 |
| 37 | neg_37 | Myricetin 3-O-glucoside | 17601.31 | 17662.09 | 18290.12 | 20.68262 | 21.6288 | 23.77132 | 5.954048 | 6.579331 | 6.77167 | 13.95009 | 15.28844 | 16.11743 |
| 38 | neg_38 | Adenosine | 76.59016 | 77.78387 | 83.07197 | 0 | 0 | 0 | 0 | 0 | 0 | 0 | 0 | 0 |
| 39 | neg_39 | (5S,6S)-di-HETE | 909.9177 | 1006.8 | 1041.91 | 1049.062 | 1069.44 | 1092.748 | 76410.38 | 79438.89 | 81892.96 | 1035.876 | 1050.622 | 1144.417 |
| 40 | neg_40 | Kaempferol 3-O-glucuronide | 2.808022 | 2.833409 | 2.960288 | 201.3913 | 202.6873 | 209.2188 | 7.43101 | 7.542988 | 7.813411 | 180.7606 | 186.7294 | 202.7296 |
| 41 | neg_41 | Astilbin | 224.1553 | 246.7888 | 253.9741 | 3.141941 | 3.472159 | 3.777811 | 1.611439 | 1.654235 | 1.692458 | 3.13135 | 3.462548 | 3.580932 |
| 42 | neg_42 | Kaempferol 3-O-glucoside | 10903.58 | 11119.34 | 11180.01 | 9950.371 | 10053.79 | 10584.87 | 10382.95 | 11002.83 | 11154.84 | 10709.49 | 11628.87 | 11679.78 |
| 43 | neg_43 | Shikimic acid | 59.36935 | 59.96266 | 62.45217 | 104.8433 | 114.5146 | 120.4436 | 169.8221 | 178.7016 | 185.8901 | 100.7613 | 107.4524 | 116.6782 |
| 44 | neg_44 | CDP-Choline | 84.38723 | 85.17784 | 91.96495 | 0 | 0 | 0 | 0 | 0 | 0 | 0 | 0 | 0 |
| 45 | neg_45 | 2-amino-2-deoxyglucitol-6-phosphate | 0 | 0 | 0 | 206.8857 | 209.7154 | 222.5082 | 124.5714 | 128.7704 | 131.8291 | 179.4167 | 185.5481 | 194.5535 |
| 46 | neg_46 | Gallic acid | 282.1895 | 284.8995 | 305.0395 | 0 | 0 | 0 | 0 | 0 | 0 | 0 | 0 | 0 |
| 47 | neg_47 | 2-Pyrocatechuic acid | 30.85821 | 32.36275 | 33.08141 | 78.88419 | 85.86876 | 88.96241 | 76.40849 | 79.40836 | 83.94222 | 46.39453 | 48.93508 | 51.71437 |
| 48 | neg_48 | Quercetin 3-O-glucoside | 65.82726 | 68.87487 | 74.59464 | 0 | 0 | 0 | 0 | 0 | 0 | 0 | 0 | 0 |
| 49 | neg_49 | PA(16:1(9Z)/0:0) | 5.747991 | 6.337854 | 6.87985 | 2800.963 | 2996.031 | 3132.304 | 0 | 0 | 0 | 10727.15 | 11086.55 | 11562.72 |
| 50 | neg_50 | Quinone | 1.288365 | 1.345723 | 1.441571 | 6.272524 | 6.604968 | 7.049673 | 3.862168 | 4.285751 | 4.565587 | 12.30445 | 12.99875 | 13.62075 |

Continued Table S2

| 51 | neg_51 | Epicatechin gallate | 6.437162 | 6.560364 | 6.951335 | 92.05707 | 98.64445 | 103.3169 | 45.24013 | 47.69948 | 51.35287 | 59.32536 | 60.01996 | 60.31513 |
| --- | --- | --- | --- | --- | --- | --- | --- | --- | --- | --- | --- | --- | --- | --- |
| 52 | neg_52 | tris-acetate | 6.917744 | 7.179886 | 7.274182 | 32.62423 | 33.94764 | 34.54456 | 25.14836 | 25.72124 | 26.06548 | 24.79957 | 25.71062 | 26.94872 |
| 53 | neg_53 | 1,2-cyclohexanedione | 95.32555 | 103.8569 | 107.3769 | 200.7812 | 206.471 | 209.8202 | 211.3628 | 224.4896 | 241.6544 | 197.4995 | 217.0501 | 229.7257 |
| 54 | neg_54 | Sucrose-6-phosphate | 33.98842 | 37.70606 | 39.7405 | 11.18097 | 11.80658 | 12.41563 | 11.18211 | 12.29825 | 13.05171 | 20.29392 | 21.24747 | 21.77859 |
| 55 | neg_55 | diacetylchitobiose-6-phosphate | 7332.431 | 8133.253 | 8729.522 | 475.0278 | 486.7061 | 530.897 | 3211.657 | 3371.821 | 3559.205 | 302.4943 | 321.168 | 334.0746 |
| 56 | neg_56 | pyrimidin-2-one-ribonucleoside | 341.9598 | 352.6379 | 361.796 | 0 | 0 | 0 | 5.030693 | 5.306474 | 5.669066 | 1.037238 | 1.059664 | 1.073311 |
| 57 | neg_57 | Inosine | 39.84882 | 40.86061 | 44.72278 | 15.82157 | 17.17091 | 18.10967 | 13.97148 | 14.05123 | 14.68144 | 0.582674 | 0.602917 | 0.644574 |
| 58 | neg_58 | dCMP | 1.187518 | 1.222531 | 1.298675 | 692.2613 | 750.3777 | 771.6147 | 517.7553 | 570.6365 | 621.9219 | 750.6305 | 782.2488 | 798.3717 |
| 59 | neg_59 | 6,7-Dimethyl-8-(1-D-ribityl)lumazine | 257.8578 | 271.5083 | 274.1161 | 594.8292 | 595.9861 | 640.3413 | 1969.004 | 2004.471 | 2026.886 | 675.4057 | 679.2926 | 686.6181 |
| 60 | neg_60 | Adenosine diphosphate ribose | 45.25482 | 49.49852 | 52.51278 | 141.0689 | 146.184 | 147.876 | 149.083 | 165.2084 | 170.013 | 186.7644 | 200.7244 | 212.6958 |
| 61 | neg_61 | 1-Nitro-7-hydroxy-8-glutathionyl-7,8-dihydronaphthalene | 78.71906 | 79.80024 | 82.74771 | 130.5956 | 135.2389 | 143.2907 | 153.619 | 158.0182 | 169.965 | 75.79684 | 83.23906 | 85.66067 |
| 62 | neg_62 | 5'-O-[N-(L-Aspartyl)sulfamoyl]adenosine | 2927.269 | 3009.748 | 3038.139 | 0.58185 | 0.643006 | 0.684456 | 1.553786 | 1.719121 | 1.817835 | 0 | 0 | 0 |
| 63 | neg_63 | 1-Phosphatidyl-D-myo-inositol | 6026.51 | 6547.541 | 7074.232 | 7315.194 | 7752.619 | 8441.764 | 7444.016 | 7676.038 | 7850.773 | 7567.689 | 8268.798 | 8965.661 |
| 64 | neg_64 | AICAR | 546.1641 | 549.2938 | 586.7731 | 190.8322 | 193.282 | 194.9713 | 2038.271 | 2138.653 | 2314.333 | 152.3803 | 154.6998 | 165.6607 |
| 65 | neg_65 | D,L-cyclohexanephosphinothricin | 215.0706 | 216.8802 | 226.8512 | 316.4846 | 328.979 | 349.2769 | 338.0595 | 358.7115 | 390.7354 | 515.6368 | 539.4844 | 545.2185 |
| 66 | neg_66 | Cyclic GMP | 107.1759 | 109.8691 | 110.1687 | 173.4456 | 183.1004 | 196.5627 | 74.43664 | 80.64489 | 83.12795 | 168.9649 | 172.7641 | 184.706 |
| 67 | neg_67 | 2-Amino-3-carboxymuconic acid semialdehyde | 0.414479 | 0.441381 | 0.469608 | 4.259945 | 4.267758 | 4.693651 | 2.269463 | 2.453282 | 2.631837 | 0.927202 | 0.956411 | 1.049814 |
| 68 | neg_68 | N-Acetyl-D-valine | 24.65022 | 25.93679 | 27.33888 | 33.9583 | 37.66405 | 39.42798 | 48.63767 | 54.02385 | 54.17924 | 25.51324 | 26.89034 | 28.5797 |
| 69 | neg_69 | 2-Hydroxy-cis-hex-2,4-dienoate | 208.9234 | 226.7772 | 236.1058 | 296.9737 | 304.2988 | 317.5448 | 4353.077 | 4371.213 | 4773.935 | 220.8653 | 240.3002 | 246.7585 |
| 70 | neg_70 | beta-ketophosphonate | 972.9348 | 1022.704 | 1031.056 | 90.45601 | 95.86056 | 102.7041 | 0 | 0 | 0 | 61.56016 | 62.83375 | 65.71818 |
| 71 | neg_71 | beta-D-Galactose | 202.9986 | 224.9715 | 243.0037 | 55.44959 | 60.59961 | 63.73913 | 65.38312 | 69.66893 | 71.41352 | 66.48081 | 71.23679 | 73.70753 |
| 72 | neg_72 | Tetrahydrodipicolinate | 287.4665 | 310.4802 | 311.1375 | 46.72534 | 51.01262 | 54.62959 | 8.499694 | 9.130671 | 9.869817 | 86.43785 | 86.67907 | 90.64007 |
| 73 | neg_73 | beta-N-acetylneuraminate | 137478.3 | 145556.8 | 158786.2 | 41.36748 | 43.16932 | 45.29805 | 8.160974 | 8.772667 | 9.592567 | 41.77512 | 45.4419 | 47.20989 |
| 74 | neg_74 | 1-(sn-glycero-3-phospho)-1D-myo-inositol | 68.54829 | 72.41609 | 76.20511 | 0 | 0 | 0 | 10.12276 | 10.36965 | 10.5576 | 0 | 0 | 0 |

Continued Table S2

| 75 | neg_75 | L-Ascorbate 6-phosphate | 17988.37 | 18034.83 | 18542.97 | 11967.44 | 12106.53 | 12537.06 | 2071.561 | 2244.598 | 2254.783 | 4223.491 | 4539.35 | 4846.332 |
| --- | --- | --- | --- | --- | --- | --- | --- | --- | --- | --- | --- | --- | --- | --- |
| 76 | neg_76 | Isovaleryl-CoA | 64.95345 | 69.1922 | 69.61849 | 0 | 0 | 0 | 0 | 0 | 0 | 0 | 0 | 0 |
| 77 | neg_77 | 5-methyl-2-furancarboxaldehyde | 233.6286 | 236.2106 | 249.496 | 0 | 0 | 0 | 0 | 0 | 0 | 0 | 0 | 0 |
| 78 | neg_78 | Flavin Mononucleotide | 79.74234 | 81.84435 | 89.17683 | 0 | 0 | 0 | 0 | 0 | 0 | 0 | 0 | 0 |
| 79 | neg_79 | Thymidine | 282.2798 | 302.6365 | 321.3267 | 0 | 0 | 0 | 0 | 0 | 0 | 0 | 0 | 0 |
| 80 | neg_80 | L-Palmitoylcarnitine | 29.02665 | 29.91281 | 31.70348 | 40.23384 | 41.83732 | 43.04949 | 360.3585 | 375.014 | 401.6506 | 27.8859 | 30.78024 | 33.48522 |
| 81 | neg_81 | N-Acetyl-D-galactosamine 6-phosphate | 7.672881 | 7.822156 | 7.877294 | 588.188 | 606.0735 | 616.2568 | 505.9518 | 508.6093 | 553.5433 | 632.0504 | 636.2596 | 677.1215 |
| 82 | neg_82 | 5-hydroxy furancarboxylic acid | 33.01194 | 33.52988 | 34.83449 | 5.241141 | 5.746527 | 5.812116 | 0.747914 | 0.830165 | 0.909486 | 13.44604 | 14.04103 | 14.72 |
| 83 | neg_83 | 5'-Phosphoribosyl-N-formylglycineamide | 77.12876 | 80.93313 | 83.09912 | 401.9924 | 407.7742 | 426.9487 | 196.8038 | 197.4696 | 210.3862 | 354.1486 | 355.811 | 366.0375 |
| 84 | neg_84 | Isochorismate | 151.7658 | 154.6663 | 156.2491 | 0 | 0 | 0 | 0 | 0 | 0 | 0 | 0 | 0 |
| 85 | neg_85 | Piperonal | 316.9401 | 321.9714 | 335.7147 | 342.1652 | 342.5001 | 354.8428 | 258.7041 | 275.7107 | 281.3609 | 397.8389 | 410.1478 | 437.961 |
| 86 | neg_86 | Citicoline | 2965.186 | 3225.232 | 3234.459 | 1726.459 | 1795.366 | 1875.607 | 1883.68 | 2006.962 | 2107.9 | 1454.832 | 1523.299 | 1648.477 |
| 87 | neg_87 | 3-(3-Hydroxyphenyl)propanoic acid | 6.841601 | 7.324511 | 7.926403 | 209.9593 | 221.9795 | 243.3587 | 45.25097 | 45.57574 | 49.92402 | 180.4998 | 193.1281 | 195.536 |
| 88 | neg_88 | 2,3-dihydrodipicolinate | 829.6946 | 866.0445 | 942.8044 | 1045.966 | 1100.493 | 1208.297 | 15264.43 | 16305.87 | 16972.78 | 775.0237 | 800.6299 | 809.8642 |
| 89 | neg_89 | fructoselysine-6-phosphate | 109.2742 | 118.4633 | 120.5183 | 90.33268 | 93.60725 | 94.77322 | 98.17807 | 108.8108 | 111.3926 | 92.25477 | 99.15023 | 100.2679 |
| 90 | neg_90 | N3-(4-methoxyfumaroyl)-L-2,3-diaminopropanoate | 533.5293 | 585.2824 | 642.5475 | 53.17352 | 58.83148 | 60.08589 | 79.0267 | 79.92645 | 86.38784 | 93.11473 | 100.7558 | 107.736 |
| 91 | neg_91 | 2-Succinyl-5-enolpyruvyl-6-hydroxy-3-cyclohexene-1-carboxylate | 6785.303 | 6949.616 | 7356 | 959.4613 | 1001.267 | 1032.477 | 972.5169 | 1063.337 | 1118.226 | 1001.687 | 1070.593 | 1174.944 |
| 92 | neg_92 | Arbutin | 20.08514 | 21.07963 | 21.73811 | 50.26061 | 50.67488 | 51.7712 | 100.4695 | 109.8818 | 116.6394 | 40.50452 | 42.45231 | 43.27377 |
| 93 | neg_93 | alpha-methylmethionine | 409.7718 | 419.8924 | 434.7801 | 753.8876 | 777.9939 | 831.9377 | 759.7803 | 761.2735 | 782.495 | 803.3185 | 814.556 | 849.3634 |
| 94 | neg_94 | 3-Deoxy-D-manno-octulosonate | 9.009179 | 9.332835 | 10.04082 | 23.96176 | 26.5418 | 28.65304 | 19.51214 | 19.82032 | 21.74481 | 26.95822 | 28.24425 | 28.38093 |
| 95 | neg_95 | azaguanine | 24.37955 | 26.86866 | 27.12066 | 102.6456 | 110.6925 | 117.1489 | 124.4946 | 127.3435 | 128.7448 | 55.251 | 59.49082 | 61.97548 |
| 96 | neg_96 | Guanosine 3'-phosphate | 1576.37 | 1606.256 | 1627.331 | 1281.954 | 1385.397 | 1479.05 | 1208.415 | 1292.616 | 1322.957 | 1288.496 | 1320.515 | 1439.192 |
| 97 | neg_97 | pteroyl-alpha-glutamylglutamate | 909.498 | 965.4759 | 1061.18 | 0.380615 | 0.411087 | 0.41747 | 0 | 0 | 0 | 0.719059 | 0.794549 | 0.812281 |
| 98 | neg_98 | L-rhamnitol | 4065.57 | 4422.161 | 4708.37 | 4645.993 | 4805.32 | 4847.604 | 4639.619 | 5066.484 | 5231.408 | 5207.588 | 5261.139 | 5395.554 |

Continued Table S2

| 99 | neg_99 | Gluconolactone | 84.37188 | 89.46738 | 94.32943 | 112.8399 | 122.0309 | 131.8329 | 92.42195 | 101.9656 | 111.8126 | 140.2068 | 149.0818 | 159.8022 |
| --- | --- | --- | --- | --- | --- | --- | --- | --- | --- | --- | --- | --- | --- | --- |
| 100 | neg_100 | 3-dehydroquinate | 12.64114 | 13.16444 | 14.10332 | 16.03608 | 16.78675 | 17.38454 | 23.52973 | 23.97651 | 25.98495 | 29.90323 | 30.98583 | 33.77519 |
| 101 | neg_101 | 5'-methylthiotubercidin | 23.12762 | 24.45376 | 25.63134 | 158.6518 | 168.6505 | 177.924 | 156.506 | 159.2953 | 170.0874 | 199.4676 | 214.5768 | 235.1644 |
| 102 | neg_102 | D-Sedoheptulose 7-phosphate | 1402.784 | 1510.788 | 1532.26 | 15.0734 | 16.42045 | 17.79898 | 12.52477 | 13.32351 | 14.01485 | 12.5355 | 13.18419 | 14.08567 |
| 103 | neg_103 | L-d-1-Pyrroline-5-carboxylic acid | 44.99449 | 49.85334 | 51.04847 | 65.6343 | 67.28231 | 67.61372 | 59.50938 | 64.10581 | 65.2929 | 61.90821 | 66.00558 | 67.76098 |
| 104 | neg_104 | Isorhamnetin 3-O-glucoside | 91.6655 | 96.43069 | 96.93751 | 0 | 0 | 0 | 0 | 0 | 0 | 0 | 0 | 0 |
| 105 | neg_105 | Maltotriose | 101.8613 | 104.9389 | 110.2601 | 548.6781 | 561.9719 | 585.8011 | 586.6983 | 617.5156 | 633.9611 | 593.3945 | 616.9273 | 669.8526 |
| 106 | neg_106 | Engeletin | 190.4934 | 201.1133 | 203.6734 | 47.358 | 52.53689 | 56.76328 | 173.2143 | 188.4625 | 190.9448 | 192.7112 | 193.8601 | 203.4727 |
| 107 | neg_107 | 5'-amino-5'-deoxyuridine | 11.84785 | 12.98835 | 13.19684 | 36.72804 | 38.50046 | 40.4224 | 2.164001 | 2.396171 | 2.407202 | 33.1558 | 36.36475 | 39.13017 |
| 108 | neg_108 | L-histidinol | 77.24272 | 80.7663 | 86.31299 | 68.80796 | 75.00779 | 77.33604 | 69.05937 | 75.70443 | 81.25402 | 84.01509 | 87.54732 | 94.07898 |
| 109 | neg_109 | Xanthurenic acid | 105.2642 | 111.2816 | 117.4489 | 8.289028 | 8.827584 | 8.880209 | 21.00216 | 21.07747 | 21.57944 | 4.494377 | 4.8916 | 5.286144 |
| 110 | neg_110 | cis-3-(3-Carboxyethenyl)-3,5-cyclohexadiene-1,2-diol | 114.971 | 118.9899 | 127.4873 | 784.6827 | 834.7194 | 894.0931 | 135.9989 | 139.6359 | 143.864 | 125.4765 | 139.2666 | 147.0454 |
| 111 | neg_111 | Delphinidin 3-O-(acetylglucoside) | 69.94623 | 70.42173 | 75.02916 | 0 | 0 | 0 | 0 | 0 | 0 | 0 | 0 | 0 |
| 112 | neg_112 | carboxin | 48.22554 | 49.68376 | 51.70155 | 34.61397 | 34.68309 | 37.77511 | 38.3024 | 41.92828 | 45.12915 | 22.80145 | 23.39364 | 24.3472 |
| 113 | neg_113 | UDPglucose | 4544.121 | 4576.875 | 4973.371 | 4736.581 | 5243.967 | 5666.375 | 4698.847 | 4904.376 | 5265.714 | 5889.302 | 6102.875 | 6211.142 |
| 114 | neg_114 | 2-thiouridine | 0 | 0 | 0 | 45.28404 | 45.58838 | 46.27783 | 23.92286 | 25.59075 | 26.93554 | 58.56769 | 59.31098 | 65.12825 |
| 115 | neg_115 | 2'-Deoxycytidine | 2849.071 | 2998.032 | 3143.167 | 3603.133 | 3978.683 | 4041.105 | 3786.935 | 3923.145 | 4181.604 | 4131.213 | 4421.358 | 4773.919 |
| 116 | neg_116 | Vanillic acid | 541.0997 | 594.0865 | 600.3995 | 4.10935 | 4.312521 | 4.563035 | 12.09956 | 12.82274 | 13.20962 | 3.129764 | 3.374247 | 3.54873 |
| 117 | neg_117 | 2,5-diamino-6-hydroxy-4-(5-phosphoribosylamino)pyrimidine | 135.4555 | 137.1136 | 146.5461 | 188.276 | 192.1854 | 196.3169 | 28.36002 | 30.68092 | 30.80201 | 127.4838 | 137.2684 | 141.574 |
| 118 | neg_118 | Propinol adenylate | 16.01994 | 17.68368 | 18.35928 | 51.93417 | 53.53886 | 56.17631 | 0.988867 | 1.072805 | 1.07631 | 65.66069 | 66.39656 | 68.17354 |
| 119 | neg_119 | Chitobiose | 238.5556 | 238.8994 | 242.7298 | 214.2422 | 228.3422 | 248.9319 | 209.7296 | 230.9489 | 252.0772 | 250.0186 | 254.8212 | 274.0837 |
| 120 | neg_120 | S-Ribosyl-L-homocysteine | 137.5427 | 151.2338 | 155.402 | 154.0944 | 155.2951 | 157.4941 | 113.2358 | 113.717 | 116.1789 | 88.9356 | 92.93558 | 96.02139 |
| 121 | neg_121 | S-adenosyl-L-(2 hydroxyl-4-methylthio)butyrate | 77.58076 | 85.49766 | 93.90235 | 43.53598 | 44.7943 | 47.10861 | 69.20544 | 74.7789 | 76.60104 | 23.43535 | 24.31677 | 25.98864 |

Continued Table S2

| 122 | neg_122 | 6-diazo-5-oxo-L-norleucine | 59.65447 | 60.14762 | 62.39001 | 0 | 0 | 0 | 38.57397 | 42.39373 | 43.7621 | 0 | 0 | 0 |
| --- | --- | --- | --- | --- | --- | --- | --- | --- | --- | --- | --- | --- | --- | --- |
| 123 | neg_123 | Cytosine | 0 | 0 | 0 | 2.997953 | 3.250169 | 3.300745 | 75.22952 | 78.24223 | 84.59181 | 5.01554 | 5.286552 | 5.506604 |
| 124 | neg_124 | 3-Hydroxy-2-methyl-4-pyrone | 801.3458 | 824.4561 | 850.6059 | 0 | 0 | 0 | 0 | 0 | 0 | 0 | 0 | 0 |
| 125 | neg_125 | Hydrocinnamic acid | 90.96466 | 92.49504 | 99.32231 | 98.96333 | 101.5789 | 110.8393 | 86.86271 | 89.29943 | 92.65646 | 100.1106 | 102.6407 | 103.1568 |
| 126 | neg_126 | P1-uridyl-P2-methyl diphosphate | 26.43972 | 27.56359 | 28.07552 | 0.96017 | 0.987296 | 1.047723 | 0 | 0 | 0 | 6.294635 | 6.778899 | 7.037028 |
| 127 | neg_127 | Nicotinamide ribotide | 103.1864 | 110.9942 | 117.7554 | 98.54428 | 104.2606 | 111.6129 | 94.07276 | 96.87611 | 100.6473 | 93.56396 | 94.92436 | 100.3759 |
| 128 | neg_128 | 7-Methylguanosine 5'-phosphate | 599.9004 | 661.7647 | 667.2039 | 80.56532 | 88.17752 | 88.3681 | 121.4443 | 131.0073 | 133.1073 | 82.27681 | 88.80401 | 95.32233 |
| 129 | neg_129 | 5'-azido-5-deoxyuridine | 1592.125 | 1760.058 | 1919.822 | 315.6154 | 318.9248 | 321.7589 | 794.7711 | 833.9229 | 858.2621 | 69.82078 | 71.05681 | 71.38759 |
| 130 | neg_130 | Ferulic acid | 132.5651 | 142.1012 | 149.2466 | 162.8799 | 171.844 | 183.3407 | 85.15109 | 88.74611 | 92.57968 | 96.30015 | 100.4817 | 107.2712 |
| 131 | neg_131 | Vitisin B | 121.7801 | 121.9941 | 127.1592 | 195.0062 | 196.8802 | 215.1501 | 133.8445 | 143.3578 | 149.3578 | 183.5466 | 192.0845 | 198.1108 |
| 132 | neg_132 | Shikimate 3-phosphate | 12821.85 | 13234.29 | 13546.68 | 11763.89 | 12488.52 | 13700.4 | 11889.02 | 12679.96 | 13902.7 | 11726.58 | 12668.9 | 13182.7 |
| 133 | neg_133 | Beta-pseudouridine | 306.1656 | 316.9422 | 325.8853 | 129.8443 | 135.1621 | 145.5621 | 301.7973 | 316.308 | 344.4407 | 8.018133 | 8.306981 | 9.13277 |
| 134 | neg_134 | Raffinose | 0 | 0 | 0 | 26.65939 | 29.41349 | 31.28526 | 8.889627 | 8.918136 | 9.529105 | 18.1386 | 18.65735 | 19.27871 |
| 135 | neg_135 | Homovanillyl alcohol | 0 | 0 | 0 | 5.702723 | 6.249887 | 6.356837 | 235.6381 | 243.1709 | 245.6372 | 90.05917 | 97.05881 | 104.3771 |
| 136 | neg_136 | Kaempferol | 146.1025 | 156.1586 | 169.9543 | 0.473662 | 0.47853 | 0.507085 | 4.34563 | 4.496868 | 4.860157 | 0 | 0 | 0 |
| 137 | neg_137 | 3-dehydro-shikimate | 98.8388 | 103.8552 | 105.5277 | 0.794916 | 0.832238 | 0.91039 | 1.650998 | 1.801411 | 1.968159 | 1.86151 | 2.048517 | 2.122522 |
| 138 | neg_138 | Uracil | 137.33 | 142.9109 | 156.644 | 115.0035 | 115.8216 | 116.9258 | 116.0929 | 118.3599 | 120.8206 | 119.8724 | 132.4176 | 135.6378 |
| 139 | neg_139 | Peonidin | 287.5011 | 305.3118 | 333.7392 | 803.4695 | 830.2645 | 880.9192 | 717.7353 | 749.3645 | 782.708 | 754.7836 | 794.9027 | 868.5673 |
| 140 | neg_140 | PA(20:4(5Z,8Z,11Z,14Z)e/2:0) | 1302.065 | 1389.744 | 1506.83 | 188.6807 | 198.2856 | 201.9144 | 382.7222 | 404.3897 | 434.615 | 250.932 | 258.9301 | 274.54 |
| 141 | neg_141 | N3-fumaramoyl-L-2,3-diaminopropanoate | 7660.888 | 8287.174 | 8726.681 | 40.06193 | 40.74889 | 42.78808 | 334.1304 | 355.4644 | 358.7897 | 12.01405 | 12.9081 | 13.79439 |
| 142 | neg_142 | 2-deoxy-D-ribose 5-phosphate | 798.468 | 815.0605 | 868.8384 | 818.6402 | 903.6657 | 960.7317 | 772.6463 | 778.1232 | 828.7544 | 855.4365 | 923.4287 | 945.174 |
| 143 | neg_143 | 4-Acetamidobutanoic acid | 0 | 0 | 0 | 62.81685 | 68.17213 | 72.40001 | 17.38993 | 18.76677 | 19.90431 | 71.30571 | 78.45045 | 83.99474 |
| 144 | neg_144 | Castavinol C2 | 0 | 0 | 0 | 115.8747 | 128.4934 | 133.88 | 119.6463 | 123.0692 | 134.5578 | 123.9631 | 137.6477 | 147.6667 |
| 145 | neg_145 | Malvidin 3-O-glucoside | 175.6158 | 182.2371 | 188.8289 | 4.780568 | 4.863818 | 5.310996 | 4.651072 | 4.769619 | 4.806095 | 1.258696 | 1.301886 | 1.360239 |
| 146 | neg_146 | gamma-glutamyl-ethylamide | 17.31384 | 18.04365 | 18.58804 | 17.84214 | 19.1416 | 19.28375 | 17.22119 | 17.38491 | 17.48942 | 19.53467 | 19.78446 | 20.39337 |

Continued Table S2

| 147 | neg_147 | (1R)-Glutathionyl-(2R)-hydroxy-1,2-dihydronaphthalene | 1180.587 | 1259.953 | 1360.682 | 220.9403 | 226.8625 | 226.9894 | 394.7343 | 431.1028 | 466.7829 | 252.56 | 274.535 | 292.6339 |
| --- | --- | --- | --- | --- | --- | --- | --- | --- | --- | --- | --- | --- | --- | --- |
| 148 | neg_148 | Beta-Alanine | 31.35349 | 34.64818 | 35.43766 | 0 | 0 | 0 | 0 | 0 | 0 | 0 | 0 | 0 |
| 149 | neg_149 | O-Acetyl-L-homoserine | 97.99618 | 102.314 | 111.9272 | 135.6086 | 139.9201 | 147.2688 | 241.1651 | 252.7523 | 269.7577 | 135.9655 | 149.687 | 159.9269 |
| 150 | neg_150 | Thiamine monophosphate | 12418.21 | 12894.13 | 13990.14 | 0 | 0 | 0 | 0 | 0 | 0 | 4.632462 | 4.685187 | 4.794064 |
| 151 | neg_151 | 2-(alpha-D-Mannosyl)-3-phosphoglycerate | 42.85743 | 46.35441 | 47.49605 | 90.07805 | 98.3738 | 106.5922 | 32.70912 | 34.94522 | 35.49003 | 59.56001 | 66.01012 | 66.85313 |
| 152 | neg_152 | cyclogutamate | 212.7564 | 225.7757 | 243.4256 | 73.21539 | 74.40698 | 77.06619 | 97.782 | 105.5348 | 115.1999 | 57.61019 | 61.11842 | 66.78437 |
| 153 | neg_153 | 2,3-dihydro-2,3-dihydroxybenzoate | 35.04856 | 36.19501 | 36.40727 | 33.00576 | 36.53146 | 37.51807 | 30.11695 | 31.63953 | 33.55873 | 33.09927 | 34.07123 | 35.36711 |
| 154 | neg_154 | 5-Phenyl-1,3-oxazinane-2,4-dione | 5.497267 | 5.564281 | 5.686474 | 24.28591 | 25.9486 | 26.17527 | 5.28476 | 5.776749 | 5.899895 | 56.13223 | 60.46723 | 65.45326 |
| 155 | neg_155 | 4-(Glutamylamino) butanoate | 44.34324 | 48.24114 | 51.12319 | 41.95645 | 44.20475 | 45.79866 | 49.47684 | 53.35123 | 55.76932 | 48.23133 | 50.21491 | 53.85146 |
| 156 | neg_156 | 5'-deoxyuridine | 1575.037 | 1658.059 | 1748.869 | 1.588218 | 1.659088 | 1.821674 | 20.46861 | 22.04836 | 23.31001 | 7.331137 | 7.525739 | 8.078387 |
| 157 | neg_157 | Isorhamnetin | 240.2774 | 258.936 | 261.638 | 225.2278 | 235.025 | 235.5579 | 275.1346 | 300.3096 | 326.5193 | 96.53548 | 99.46438 | 105.0846 |
| 158 | neg_158 | xanthosine | 92.04406 | 96.21303 | 100.946 | 8.795146 | 9.027711 | 9.849396 | 3.945127 | 4.311214 | 4.705703 | 2.407243 | 2.504495 | 2.647478 |
| 159 | neg_159 | 5'-ethylthioadenosine | 0 | 0 | 0 | 495.9345 | 500.5 | 526.9688 | 696.9068 | 728.5055 | 759.7351 | 1412.235 | 1528.058 | 1551.361 |
| 160 | neg_160 | Malonic semialdehyde | 59.1603 | 64.00848 | 66.83355 | 37.40581 | 38.96037 | 41.71365 | 31.32307 | 34.6341 | 38.04906 | 47.56958 | 47.60287 | 50.08932 |
| 161 | neg_161 | Ureidopropionic acid | 52.66036 | 54.97731 | 57.80246 | 0 | 0 | 0 | 0.2577 | 0.275363 | 0.294895 | 0 | 0 | 0 |
| 162 | neg_162 | SAICAR | 284.3038 | 290.661 | 313.2327 | 39.03528 | 42.61799 | 43.33805 | 29.95077 | 31.18098 | 33.54798 | 33.27289 | 36.48638 | 37.0942 |
| 163 | neg_163 | Riboflavin | 247.8179 | 254.8051 | 274.8155 | 35.88729 | 39.00942 | 41.49509 | 32.86531 | 33.148 | 34.87961 | 27.11579 | 29.26652 | 29.46404 |
| 164 | neg_164 | Porphobilinogen | 36.13646 | 38.24005 | 39.6221 | 36.37858 | 39.59956 | 41.75649 | 47.31041 | 47.4342 | 51.64829 | 37.52819 | 38.66651 | 39.87969 |
| 165 | neg_165 | 3',5'-cyclic dAMP | 173.7683 | 174.112 | 174.4748 | 0 | 0 | 0 | 0 | 0 | 0 | 0 | 0 | 0 |
| 166 | neg_166 | Cyanidin 3-O-(acetylglucoside) | 117.2866 | 122.9405 | 123.8222 | 18.07053 | 18.21607 | 19.13751 | 0 | 0 | 0 | 9.681144 | 10.28935 | 11.14665 |
| 167 | neg_167 | formycin B | 233.2632 | 236.6461 | 251.778 | 6.476467 | 6.500087 | 6.58436 | 80.90193 | 86.50441 | 88.87211 | 1.36542 | 1.469426 | 1.481263 |
| 168 | neg_168 | (1R,6R)-6-Hydroxy-2-succinylcyclohexa-2,4-diene-1-carboxylate | 9.225209 | 9.727803 | 9.855001 | 97.14544 | 99.22487 | 102.8193 | 128.3327 | 133.1236 | 141.3752 | 69.26137 | 76.69275 | 84.14633 |
| 169 | neg_169 | pyrithiamine phosphate | 12.15557 | 13.23546 | 13.31072 | 79.7153 | 81.05951 | 85.81242 | 42.40702 | 46.54296 | 47.01981 | 102.6339 | 107.3068 | 111.166 |
| 170 | neg_170 | N-Formyl-L-tyrosine | 19.01758 | 20.49148 | 21.73205 | 57.34715 | 60.86949 | 63.93742 | 67.06376 | 69.87557 | 71.07762 | 68.84888 | 70.55009 | 70.73899 |

Continued Table S2

| 171 | neg_171 | Petunidin 3-O-(acetylglucoside) | 78.51142 | 81.63699 | 84.91684 | 276.6804 | 297.5034 | 320.2143 | 179.5716 | 190.8886 | 202.4043 | 372.8352 | 373.9026 | 406.7141 |
| --- | --- | --- | --- | --- | --- | --- | --- | --- | --- | --- | --- | --- | --- | --- |
| 172 | neg_172 | Biotinyl-5'-AMP | 54.3446 | 55.64242 | 60.87992 | 34.09859 | 37.7506 | 41.50902 | 68.99226 | 76.53847 | 78.33413 | 23.76219 | 23.93324 | 25.16161 |
| 173 | neg_173 | L-Cystine | 49.31213 | 51.35478 | 53.26529 | 48.17093 | 51.96179 | 52.67265 | 46.67274 | 51.28771 | 53.39007 | 40.91819 | 42.76313 | 45.68084 |
| 174 | neg_174 | Deoxyguanosine | 38.2392 | 39.2975 | 41.0967 | 0 | 0 | 0 | 0 | 0 | 0 | 0 | 0 | 0 |
| 175 | neg_175 | 4-(Cytidine 5'-diphospho)-2-C-methyl-D-erythritol | 6.156629 | 6.785656 | 6.857388 | 16.98039 | 18.46604 | 19.13678 | 17.83633 | 19.15818 | 20.61736 | 5.755373 | 5.994766 | 6.287377 |
| 176 | neg_176 | PE(18:1(11Z)/18:1(11Z)) | 5163.362 | 5262.019 | 5743.311 | 4544.071 | 4577.936 | 4733.955 | 4323.339 | 4406.106 | 4623.934 | 4323.048 | 4732.041 | 4885.718 |
| 177 | neg_177 | coformycin | 127.2401 | 135.9297 | 147.0151 | 59.34636 | 63.09501 | 64.40208 | 59.89733 | 64.0457 | 64.53276 | 53.64488 | 59.16038 | 64.16994 |
| 178 | neg_178 | 2-(6-Hydroxy-2-methoxy-3,4-methylenedioxyphenyl)benzofuran | 3453.305 | 3470.285 | 3471.567 | 307.3283 | 317.3948 | 323.9835 | 416.0428 | 419.9698 | 454.8873 | 269.4741 | 275.3061 | 292.2891 |
| 179 | neg_179 | Beta-D-Fructose 6-phosphate | 102.0316 | 108.0288 | 117.2338 | 195.1588 | 196.9037 | 197.6586 | 229.9932 | 252.354 | 263.5976 | 127.4677 | 139.25 | 151.2617 |
| 180 | neg_180 | 2,3-Dihydro-2,5-dihydoxy-6-methyl-4-H-pyran-4-one | 273.7111 | 279.2548 | 281.0892 | 0 | 0 | 0 | 0 | 0 | 0 | 0 | 0 | 0 |
| 181 | neg_181 | Pyrophosphate | 927.1469 | 974.6206 | 1028.795 | 845.7261 | 906.2607 | 906.4051 | 941.9786 | 999.5733 | 1096.7 | 1022.868 | 1072.066 | 1085.406 |
| 182 | neg_182 | 2-(S-Glutathionyl)acetyl glutathione | 7.346757 | 8.04201 | 8.333569 | 60.00042 | 64.40789 | 65.98536 | 52.84446 | 55.69882 | 58.55961 | 58.25771 | 59.01011 | 64.12183 |
| 183 | neg_183 | Quercetin | 44.47565 | 47.41006 | 48.48521 | 1.405943 | 1.557515 | 1.648254 | 10.02119 | 10.98445 | 11.40125 | 0 | 0 | 0 |
| 184 | neg_184 | 5-O-(1-Carboxyvinyl)-3-phosphoshikimate | 8282.074 | 8553.417 | 8984.738 | 7893.852 | 8053.873 | 8282.093 | 8274.615 | 8891.06 | 8921.012 | 7735.523 | 8468.454 | 9123.904 |
| 185 | neg_185 | N2-Succinyl-L-ornithine | 751.9021 | 798.0174 | 806.8991 | 10.86218 | 11.58208 | 12.0968 | 47.12113 | 51.16992 | 55.3602 | 36.25898 | 39.43265 | 41.78698 |
| 186 | neg_186 | L-histidinol phosphate | 0.60079 | 0.603311 | 0.655135 | 4.940105 | 5.244635 | 5.273098 | 7.293716 | 7.410831 | 8.009748 | 3.952998 | 4.253964 | 4.262477 |
| 187 | neg_187 | 4-Allyl-2-methoxyphenol | 32.77063 | 33.84556 | 34.98093 | 46.1739 | 48.07483 | 49.48178 | 42.68138 | 45.80154 | 48.99952 | 53.51414 | 56.27535 | 60.70609 |
| 188 | neg_188 | 2,5-Diamino-6-(5'-phosphoribosylamino)-4-pyrimidineone | 6.145669 | 6.504313 | 6.682848 | 49.87984 | 53.12104 | 54.56177 | 12.66884 | 13.18756 | 13.9785 | 34.80146 | 35.93767 | 36.94634 |
| 189 | neg_189 | Cyclotene | 2671.429 | 2916.937 | 3163.021 | 0 | 0 | 0 | 1.281413 | 1.320962 | 1.329554 | 0 | 0 | 0 |
| 190 | neg_190 | (2,3-Dihydroxybenzoyl)adenylate | 81.80481 | 87.56894 | 93.70104 | 10.81094 | 11.88722 | 12.41323 | 0 | 0 | 0 | 0 | 0 | 0 |
| 191 | neg_191 | 5-methylaminomethyl-2-thiouridine | 1661.024 | 1685.458 | 1750.178 | 618.0128 | 662.4441 | 685.0494 | 1095.929 | 1205.794 | 1298.813 | 473.1505 | 484.1907 | 504.7456 |
| 192 | neg_192 | Acetylphosphate | 26.36328 | 28.48569 | 30.89412 | 21.83274 | 22.16341 | 22.75253 | 11.81263 | 13.04136 | 13.98168 | 22.73529 | 24.12681 | 24.46487 |

Continued Table S2

| 193 | neg_193 | Deoxyribose 1-phosphate | 97.64174 | 100.6279 | 101.6389 | 86.38115 | 95.38565 | 96.09275 | 104.017 | 113.7012 | 115.8215 | 93.56469 | 100.1322 | 108.5902 |
| --- | --- | --- | --- | --- | --- | --- | --- | --- | --- | --- | --- | --- | --- | --- |
| 194 | neg_194 | O-acetylcarnitinium | 129.0338 | 139.6447 | 147.4727 | 0 | 0 | 0 | 3.653603 | 3.728337 | 4.037764 | 0 | 0 | 0 |
| 195 | neg_195 | FMNH | 5.449836 | 5.593527 | 6.056907 | 51.04821 | 51.69631 | 54.81989 | 42.16481 | 43.33808 | 47.61868 | 0 | 0 | 0 |
| 196 | neg_196 | Glucosan | 27.75635 | 28.12939 | 29.15995 | 30.22009 | 32.51187 | 33.29369 | 25.67854 | 27.06081 | 28.31916 | 27.74877 | 29.2806 | 30.16277 |
| 197 | neg_197 | N-Formylanthranilic acid | 46.10228 | 48.41633 | 52.97355 | 53.61088 | 54.0346 | 59.07234 | 40.66001 | 41.59312 | 42.17157 | 41.86629 | 45.4283 | 49.23175 |
| 198 | neg_198 | 1-Nitro-7-glutathionyl-8-hydroxy-7,8-dihydronaphthalene | 23.02458 | 23.52707 | 25.39836 | 25.23227 | 28.02684 | 29.98144 | 17.79777 | 18.39869 | 20.06782 | 25.7416 | 28.19484 | 30.93617 |
| 199 | neg_199 | Anethole | 20.85159 | 21.39268 | 22.88927 | 24.2551 | 24.95535 | 26.93165 | 2376.62 | 2557.914 | 2604.257 | 20.78811 | 21.28656 | 22.83032 |
| 200 | neg_200 | Carnosine | 14.26603 | 14.58942 | 14.72106 | 10.49491 | 10.89523 | 11.77782 | 18.57269 | 19.89776 | 21.00356 | 27.24909 | 29.18704 | 31.74514 |
| 201 | neg_201 | D-Phenylalanine | 491.9875 | 532.893 | 579.4972 | 312.5314 | 314.8034 | 333.7997 | 574.8827 | 581.6918 | 587.4415 | 461.57 | 477.0144 | 517.1996 |
| 202 | neg_202 | Salicin 6-phosphate | 59.8711 | 65.03639 | 69.70407 | 1.585825 | 1.726592 | 1.803831 | 45.4597 | 47.09935 | 48.29713 | 0 | 0 | 0 |
| 203 | neg_203 | 4-methylumbelliferone | 294.2441 | 299.8342 | 302.4898 | 0.046303 | 0.049408 | 0.052841 | 1.121943 | 1.135942 | 1.167076 | 0.162707 | 0.176538 | 0.18296 |
| 204 | neg_204 | TDP-rhamnose | 3.515207 | 3.515391 | 3.625653 | 0 | 0 | 0 | 2.673961 | 2.877449 | 2.890319 | 3.423189 | 3.792254 | 3.945041 |
| 205 | neg_205 | dimethyltrisulfide | 2099.167 | 2126.497 | 2202.561 | 1913.297 | 2093.986 | 2139.7 | 1762.481 | 1891.983 | 1999.564 | 1839.115 | 2021.144 | 2185.343 |
| 206 | neg_206 | UDP-Glucuronic acid | 61.78213 | 67.32517 | 72.6366 | 60.06745 | 61.49056 | 66.53524 | 62.2465 | 63.4836 | 68.09171 | 60.67398 | 64.96949 | 68.12919 |
| 207 | neg_207 | alpha-(2,6-anhydro-3-deoxy-D-arabino-heptulopyranosid)onate 7-phosphonate | 13.24589 | 14.18854 | 15.49245 | 17.08674 | 17.95341 | 19.31469 | 10.92656 | 11.02516 | 11.83764 | 13.22659 | 14.63108 | 14.82818 |
| 208 | neg_208 | Mevalonic acid-5P | 21.64457 | 21.91641 | 23.88717 | 1.024674 | 1.028614 | 1.034996 | 0.857277 | 0.941411 | 1.000638 | 1.120094 | 1.1718 | 1.284009 |
| 209 | neg_209 | L-Sorbose | 70.51742 | 70.65987 | 75.66643 | 31.54228 | 32.42018 | 34.36465 | 25.84033 | 25.9077 | 27.08925 | 33.70065 | 34.45465 | 37.21309 |
| 210 | neg_210 | 5-Amino-6-(5'-phosphoribitylamino)uracil | 447.4724 | 467.3032 | 501.7467 | 11.23339 | 11.51468 | 12.44032 | 59.55142 | 62.62647 | 63.13746 | 4.412733 | 4.664564 | 5.109337 |
| 211 | neg_211 | L-seryl-AMP | 99.99067 | 104.3041 | 107.7252 | 28.53828 | 29.37328 | 30.74717 | 21.22901 | 22.1231 | 22.15634 | 27.18028 | 27.36821 | 27.94298 |
| 212 | neg_212 | D-Ribulose 5-phosphate | 10253.46 | 10298.66 | 11167.42 | 9650.572 | 9737.406 | 10461.64 | 8723.852 | 9691.361 | 9762.279 | 8972.386 | 9862.961 | 10253.88 |
| 213 | neg_213 | 5-Thymidylic acid | 33.46703 | 35.924 | 38.80565 | 27.7306 | 28.75371 | 29.46797 | 80.39123 | 85.43117 | 88.99181 | 16.31816 | 16.44626 | 16.94027 |
| 214 | neg_214 | DIMBOA-Glc | 107.802 | 117.9694 | 118.0275 | 1.130883 | 1.158495 | 1.231246 | 0 | 0 | 0 | 0 | 0 | 0 |
| 215 | neg_215 | D-Fructuronate | 68.68659 | 68.95395 | 75.72667 | 63.94696 | 68.27494 | 73.17237 | 57.19362 | 61.61105 | 63.30502 | 66.42075 | 70.80592 | 76.16328 |
| 216 | neg_216 | Catechin | 60064.81 | 60527.28 | 63473.01 | 1078.866 | 1080.893 | 1109.446 | 4140.458 | 4531.872 | 4960.734 | 901.6748 | 905.9559 | 976.787 |

Continued Table S2

| 217 | neg_217 | Pyrrolidonecarboxylic acid | 80.1177 | 81.55624 | 85.91388 | 4.04373 | 4.435928 | 4.721321 | 32.81193 | 35.01167 | 37.49278 | 1.01523 | 1.117092 | 1.118813 |
| --- | --- | --- | --- | --- | --- | --- | --- | --- | --- | --- | --- | --- | --- | --- |
| 218 | neg_218 | S-Adenosylhomocysteine | 248.2602 | 252.5365 | 271.7364 | 0 | 0 | 0 | 0 | 0 | 0 | 0 | 0 | 0 |
| 219 | neg_219 | cephalexin | 422.9878 | 465.6573 | 479.4263 | 0 | 0 | 0 | 0 | 0 | 0 | 0 | 0 | 0 |
| 220 | neg_220 | Isopropylmaleate | 310.7426 | 311.4858 | 314.3776 | 1.008888 | 1.059876 | 1.085052 | 161.1114 | 167.9933 | 173.4643 | 0 | 0 | 0 |
| 221 | neg_221 | N2-hydroxyguanosine 5'-monophosphate | 271.1126 | 300.3182 | 323.9389 | 230.58 | 238.1954 | 254.0367 | 192.9569 | 203.1462 | 212.3739 | 214.0481 | 233.3353 | 239.9775 |
| 222 | neg_222 | Myricetin 3-O-glucuronide | 102.1414 | 106.7655 | 113.815 | 66.52534 | 70.78935 | 77.35403 | 56.87939 | 62.51302 | 65.88705 | 67.37602 | 71.32268 | 78.13628 |
| 223 | neg_223 | Castavinol C1 | 248.2061 | 262.781 | 280.085 | 4433.487 | 4594.196 | 4896.722 | 4070.731 | 4503.197 | 4532.665 | 4458.833 | 4591.553 | 4934.855 |
| 224 | neg_224 | 2-formamido-N(1)-(5-phospho-D-ribosyl)acetamidine | 301.9042 | 304.9259 | 306.4082 | 1243.748 | 1374.552 | 1460.361 | 1598.612 | 1756.939 | 1866.985 | 1035.382 | 1071.7 | 1150.736 |
| 225 | neg_225 | trans-Aconitic acid | 53.89112 | 55.23037 | 57.94268 | 9.143418 | 9.555986 | 10.47799 | 10.42841 | 10.95509 | 11.97108 | 2.292882 | 2.521059 | 2.640001 |
| 226 | neg_226 | D-Lactic acid | 71.42033 | 77.11496 | 82.36271 | 77.65055 | 84.32771 | 89.31921 | 80.07463 | 82.23972 | 87.24891 | 101.1946 | 101.6515 | 107.0703 |
| 227 | neg_227 | D-Pantothenoyl-L-cysteine | 163.3167 | 179.1353 | 182.4394 | 232.6325 | 241.6388 | 247.0927 | 125.0423 | 138.0786 | 146.4821 | 209.9054 | 232.3566 | 244.2244 |
| 228 | neg_228 | cellobiose-6-phosphate | 79.92251 | 79.94435 | 81.19072 | 10.65787 | 11.71809 | 12.21274 | 17.15044 | 17.54312 | 18.04794 | 2.060516 | 2.18249 | 2.224825 |
| 229 | neg_229 | PI(18:0/18:1(9Z)) | 260.9849 | 276.199 | 287.7524 | 357.0945 | 366.5282 | 384.798 | 236.794 | 258.9683 | 268.542 | 254.3889 | 278.3809 | 289.1857 |
| 230 | neg_230 | 4-deoxypyridoxine 5'-phosphate | 0.846067 | 0.935697 | 1.021224 | 1.87657 | 1.971503 | 1.988615 | 2.46196 | 2.623405 | 2.785431 | 3.464869 | 3.611758 | 3.777571 |
| 231 | neg_231 | (2S,3S)-2,3-Dihydro-2,3-dihydroxybenzoate | 329.5539 | 338.7446 | 367.7297 | 180.0243 | 181.2223 | 183.4156 | 255.0565 | 274.9542 | 298.6419 | 105.2868 | 106.2316 | 108.7372 |
| 232 | neg_232 | Delphinidin 3-O-glucoside | 0 | 0 | 0 | 8.606577 | 9.549249 | 10.28 | 0.781296 | 0.802736 | 0.816502 | 6.266758 | 6.589247 | 6.855294 |
| 233 | neg_233 | P1-uridyl-P2-phenyl diphosphate | 276.7188 | 277.2647 | 277.7835 | 257.0153 | 261.4931 | 278.0315 | 238.4339 | 261.8235 | 278.1916 | 232.1183 | 251.9774 | 257.1335 |
| 234 | neg_234 | S-Lactoylglutathione | 0 | 0 | 0 | 97.4512 | 101.6986 | 105.0777 | 5.267622 | 5.322715 | 5.597741 | 37.94031 | 38.80264 | 38.91881 |
| 235 | neg_235 | NMNH | 75.72984 | 83.1471 | 87.38506 | 0 | 0 | 0 | 5.315047 | 5.340395 | 5.710792 | 0 | 0 | 0 |
| 236 | neg_236 | 5-Aminoimidazole ribonucleotide | 130.0854 | 135.3346 | 148.4434 | 14.76 | 15.77221 | 16.23008 | 13.44067 | 13.90565 | 14.15117 | 5.744811 | 6.076631 | 6.665537 |
| 237 | neg_237 | Nicotinic acid mononucleotide | 525.5589 | 572.8126 | 618.001 | 483.247 | 483.6951 | 493.6568 | 404.5602 | 404.9915 | 424.9584 | 426.6936 | 456.6342 | 457.2609 |
| 238 | neg_238 | Cytidine monophosphate | 11.1592 | 11.55318 | 12.02759 | 22.5442 | 23.84804 | 26.14844 | 25.82832 | 27.48864 | 29.53093 | 24.42828 | 24.70273 | 26.05972 |
| 239 | neg_239 | Deoxycytidine | 57.40242 | 60.04297 | 61.20765 | 48.05398 | 53.23811 | 58.07171 | 42.45251 | 44.21564 | 45.21455 | 53.94837 | 57.30726 | 59.64373 |
| 240 | neg_240 | Kynurenic acid | 35.69503 | 37.6945 | 38.64086 | 5.847131 | 6.231326 | 6.78479 | 17.69045 | 18.97563 | 20.29577 | 0 | 0 | 0 |
| 241 | neg_241 | 4-Hydroxy-2-oxoglutaric acid | 228.218 | 247.6326 | 262.6777 | 193.3356 | 211.3791 | 224.6628 | 215.9658 | 229.3084 | 245.7643 | 161.4771 | 175.6541 | 179.714 |

Continued Table S2

| 242 | neg_242 | PS(19:0/16:0) | 15550.46 | 16637.04 | 17776.01 | 14536.34 | 15454.57 | 15793.87 | 13397.73 | 14498.21 | 15821.79 | 10538.63 | 11039.73 | 11330.38 |
| --- | --- | --- | --- | --- | --- | --- | --- | --- | --- | --- | --- | --- | --- | --- |
| 243 | neg_243 | (R)-pantolactone | 0 | 0 | 0 | 29.90588 | 30.93471 | 31.85269 | 0 | 0 | 0 | 67.59068 | 71.48891 | 78.00504 |
| 244 | neg_244 | Trypanothione disulfide | 0 | 0 | 0 | 216.2647 | 216.5064 | 236.4645 | 0 | 0 | 0 | 72.17315 | 79.36105 | 82.65415 |
| 245 | neg_245 | 3,4-dimethylenedioxy mandelic acid | 6.806116 | 7.544839 | 7.98998 | 1203.594 | 1274.439 | 1361.824 | 1320.702 | 1434.26 | 1460.878 | 1027.185 | 1035.308 | 1067.006 |
| 246 | neg_246 | Melibiitol | 34.53331 | 36.96989 | 40.66259 | 77.25463 | 83.42857 | 83.58828 | 76.28552 | 82.33507 | 83.01085 | 76.06294 | 83.69988 | 91.696 |
| 247 | neg_247 | Phosphoribosyl formamidocarboxamide | 0.860001 | 0.916459 | 0.917114 | 3.687331 | 3.898314 | 4.187889 | 2.09147 | 2.275265 | 2.492204 | 1.547188 | 1.571831 | 1.600942 |
| 248 | neg_248 | S-carboxymethyl-D-cysteine | 34.5087 | 38.3024 | 38.58669 | 39.43124 | 42.71351 | 46.77809 | 37.47591 | 38.54229 | 41.37215 | 42.19732 | 42.50675 | 42.83828 |
| 249 | neg_249 | PE(18:1(11Z)/19:0) | 693.2096 | 694.3806 | 728.1264 | 76.40637 | 81.32237 | 87.45774 | 454.4632 | 480.9737 | 489.7756 | 1.168865 | 1.194754 | 1.310855 |
| 250 | neg_250 | 3-Oxo-7,8-dihydro-alpha-ionol | 0 | 0 | 0 | 13.97999 | 15.49763 | 16.09908 | 100.1889 | 106.8921 | 108.7946 | 0 | 0 | 0 |
| 251 | neg_251 | 4-Hydroxy-2-oxohexanoic acid | 92170.13 | 93490.07 | 102466.2 | 33.81284 | 37.20573 | 38.9546 | 158.0395 | 159.8653 | 162.8983 | 189.3073 | 193.988 | 199.2182 |
| 252 | neg_252 | PI(18:1(9Z)/18:1(9Z)) | 56.55533 | 57.14435 | 62.24678 | 70.79327 | 77.53631 | 80.72721 | 63.77844 | 67.99463 | 69.61183 | 38.86846 | 41.31889 | 43.60781 |
| 253 | neg_253 | 7,8-dihydroneopterin 3'-phosphate | 109.9581 | 118.4161 | 121.7407 | 0 | 0 | 0 | 0 | 0 | 0 | 0 | 0 | 0 |
| 254 | neg_254 | S-adenosyl-4-methylthio-2-oxobutanoic acid | 46.07304 | 47.1092 | 49.30778 | 102.4848 | 103.3637 | 103.973 | 64.2884 | 65.94496 | 69.52563 | 106.8492 | 117.3472 | 122.7985 |
| 255 | neg_255 | Ornithine | 21.54083 | 22.47126 | 24.17243 | 19.78398 | 20.34588 | 21.42305 | 38.8403 | 42.71992 | 46.72381 | 22.39978 | 23.33142 | 23.94329 |
| 256 | neg_256 | N-Acetyl-D-tyrosine | 1596.972 | 1745.34 | 1827.865 | 0 | 0 | 0 | 0 | 0 | 0 | 0 | 0 | 0 |
| 257 | neg_257 | penicillin G | 15.02371 | 16.6596 | 18.11444 | 82.53675 | 83.27304 | 86.15744 | 29.13508 | 31.10646 | 34.03813 | 20.92304 | 22.02564 | 22.32494 |
| 258 | neg_258 | UDP-N-acetylmuramoyl-L-alanyl-gamma-D-glutamyl-L-lysine | 0 | 0 | 0 | 86.39498 | 94.72534 | 96.53492 | 90.37144 | 99.40296 | 102.4673 | 108.2116 | 116.8362 | 125.4426 |
| 259 | neg_259 | Caftaric acid | 0 | 0 | 0 | 94.91831 | 104.4318 | 114.2231 | 102.4921 | 111.7302 | 122.7253 | 78.59913 | 84.09233 | 92.21472 |
| 260 | neg_260 | 2',3'-Cyclic UMP | 1248.768 | 1257.896 | 1287.551 | 1328.838 | 1343.857 | 1436.391 | 1420.954 | 1461.765 | 1466.649 | 1210.881 | 1254.306 | 1340.292 |
| 261 | neg_261 | cis-3-(Carboxy-ethyl)-3,5-cyclo-hexadiene-1,2-diol | 42.65187 | 46.1143 | 47.72127 | 7.449287 | 8.236489 | 8.297493 | 16.95774 | 17.32622 | 18.33797 | 6.088487 | 6.508754 | 6.997943 |
| 262 | neg_262 | 2'-Deoxyguanosine 5'-monophosphate | 44.11376 | 48.61899 | 53.04631 | 4.654065 | 5.169905 | 5.312738 | 35.99235 | 39.14257 | 40.88384 | 12.72407 | 13.33033 | 13.37052 |
| 263 | neg_263 | 7,8-Dihydro-7-hydroxy-8-S-glutathionyl-benzo[a]pyrene | 38.23255 | 40.08307 | 41.3142 | 0 | 0 | 0 | 0 | 0 | 0 | 0 | 0 | 0 |
| 264 | neg_264 | 3-Hydroxy-5-methylhex-4-enoyl-CoA | 44.1244 | 46.5731 | 49.608 | 1.340493 | 1.489234 | 1.536402 | 43.46187 | 43.51365 | 46.12678 | 0 | 0 | 0 |

Continued Table S2

| 265 | neg_265 | Oxalacetic acid | 212.0887 | 226.644 | 247.3556 | 224.5984 | 244.8579 | 256.8477 | 225.591 | 227.122 | 243.5343 | 214.4513 | 217.0759 | 217.5613 |
| --- | --- | --- | --- | --- | --- | --- | --- | --- | --- | --- | --- | --- | --- | --- |
| 266 | neg_266 | dTDP-D-glucose | 27.73481 | 28.08715 | 29.35685 | 27.1075 | 27.94888 | 28.43317 | 15.19071 | 16.46673 | 17.732 | 32.58538 | 35.46496 | 37.81176 |
| 267 | neg_267 | 3',5'-cyclic CMP | 79.12439 | 87.42976 | 93.81197 | 2.101218 | 2.111018 | 2.204654 | 0 | 0 | 0 | 0 | 0 | 0 |
| 268 | neg_268 | Castavinol C3 | 0 | 0 | 0 | 150.4875 | 154.6239 | 168.5686 | 100.562 | 110.7529 | 115.8453 | 131.8303 | 137.5289 | 142.9894 |
| 269 | neg_269 | N-(quinolin-8-yl)methanesulfonamide | 799.9096 | 813.5116 | 823.9222 | 0.734328 | 0.737789 | 0.804774 | 0 | 0 | 0 | 4.473054 | 4.605214 | 5.007612 |
| 270 | neg_270 | Glutathione episulfonium ion | 469.0516 | 519.2154 | 554.2036 | 14.88891 | 15.11703 | 16.23003 | 16.34627 | 17.50783 | 18.91213 | 16.61761 | 17.11576 | 18.46205 |
| 271 | neg_271 | Glycineamideribotide | 35.12128 | 37.22454 | 38.96916 | 10.65449 | 11.47339 | 11.5685 | 25.81989 | 26.14607 | 26.34187 | 14.29462 | 14.95234 | 15.29411 |
| 272 | neg_272 | PS(18:1(11Z)/19:0) | 4930.111 | 5005.979 | 5034.298 | 1155.364 | 1198.488 | 1237.043 | 2302.03 | 2311.883 | 2381.891 | 866.8556 | 937.1769 | 954.8347 |
| 273 | neg_273 | bis-(2-methyl-3-furyl) disulfide | 499.6138 | 523.6919 | 538.2396 | 485.2907 | 536.0143 | 553.6654 | 497.0105 | 512.5902 | 513.4256 | 444.0926 | 476.6267 | 484.9047 |
| 274 | neg_274 | 2-furfurylmethylsulfide | 113.1778 | 122.6656 | 128.322 | 8.429076 | 8.432551 | 9.248038 | 5.875465 | 6.344266 | 6.784438 | 5.734758 | 5.959656 | 6.15174 |
| 275 | neg_275 | dTDP | 62.26289 | 65.89335 | 71.35504 | 66.20814 | 67.46272 | 73.85547 | 59.50801 | 64.15305 | 68.70305 | 66.93154 | 67.80483 | 73.89663 |
| 276 | neg_276 | Indoleacetaldehyde | 836.0124 | 872.9584 | 927.9074 | 27.60984 | 28.27267 | 30.45799 | 33.55238 | 35.64085 | 35.80256 | 18.36063 | 19.22573 | 19.98765 |
| 277 | neg_277 | Malvidin glucoside-ethyl-catechin | 152.1315 | 152.3796 | 163.607 | 789.0968 | 843.554 | 866.5074 | 129.4628 | 138.0431 | 143.7608 | 804.3298 | 855.6925 | 869.7959 |
| 278 | neg_278 | dipropyl succinate | 550.5192 | 599.7998 | 637.2236 | 111.0174 | 115.7404 | 125.4381 | 268.2663 | 292.5115 | 299.8332 | 113.1171 | 119.8079 | 124.6116 |
| 279 | neg_279 | O-Succinyl-L-homoserine | 566.6607 | 608.6693 | 612.5009 | 0.927369 | 1.013407 | 1.041466 | 4.004588 | 4.091688 | 4.275777 | 3.773684 | 4.163583 | 4.309888 |
| 280 | neg_280 | CDP-Ethanolamine | 196.4106 | 217.0277 | 231.665 | 266.8261 | 278.0882 | 287.7123 | 235.9567 | 261.4692 | 275.7736 | 231.5722 | 247.9792 | 256.0852 |
| 281 | neg_281 | Cytidine | 41.80426 | 45.41293 | 47.79808 | 29.28213 | 31.75584 | 32.92949 | 23.90529 | 24.421 | 25.91519 | 29.97172 | 33.13652 | 35.65204 |
| 282 | neg_282 | L-Aspartate-semialdehyde | 94.31487 | 98.49114 | 104.8201 | 0 | 0 | 0 | 0 | 0 | 0 | 0 | 0 | 0 |
| 283 | neg_283 | S-D-Lactoylglutathione | 119.4691 | 123.7918 | 128.2495 | 1745.073 | 1747.324 | 1821.983 | 2623.88 | 2708.897 | 2754.061 | 958.468 | 959.9545 | 1054.27 |
| 284 | neg_284 | Glycerylphosphorylethanolamine | 30.23624 | 30.57978 | 30.72212 | 29.08647 | 30.54925 | 31.79999 | 34.55363 | 36.73457 | 37.27153 | 33.76272 | 35.33402 | 36.7731 |
| 285 | neg_285 | Glutathione | 42.07288 | 46.4909 | 47.5794 | 28.24558 | 28.38004 | 30.60335 | 29.14054 | 31.16309 | 31.37569 | 19.22293 | 19.60528 | 20.95974 |
| 286 | neg_286 | fructoselysine | 112.0403 | 120.6904 | 132.2318 | 97.30298 | 101.4974 | 103.3564 | 89.46186 | 90.97687 | 91.73473 | 94.36917 | 103.9804 | 108.3595 |
| 287 | neg_287 | Dihydrolipoamide | 1408.311 | 1415.528 | 1515.175 | 755.31 | 801.3562 | 828.5921 | 736.0092 | 797.5267 | 849.4404 | 851.5743 | 918.199 | 944.5875 |
| 288 | neg_288 | GDP-L-fucose | 109.0337 | 120.4965 | 128.9274 | 40.8431 | 43.87399 | 47.43559 | 7.405358 | 8.118906 | 8.348992 | 72.83087 | 74.32938 | 79.03204 |
| 289 | neg_289 | D-Erythro-imidazole-glycerol-phosphate | 64.9442 | 69.44736 | 73.87036 | 69.09753 | 69.70502 | 71.83539 | 60.1726 | 66.42198 | 69.91773 | 61.6488 | 63.23578 | 63.86425 |
| 290 | neg_290 | L-2-Amino-3-oxobutanoic acid | 148.4604 | 159.0107 | 173.5165 | 207.6896 | 220.8338 | 220.9485 | 244.8283 | 267.987 | 278.9804 | 243.9665 | 250.4496 | 253.7835 |

Continued Table S2

| 291 | neg_291 | D-galactal | 20889.24 | 22095.88 | 22095.91 | 0 | 0 | 0 | 3.068656 | 3.169468 | 3.287484 | 12.88671 | 13.39039 | 14.36443 |
| --- | --- | --- | --- | --- | --- | --- | --- | --- | --- | --- | --- | --- | --- | --- |
| 292 | neg_292 | UDP-D-galacto-1,4-furanose | 87.09178 | 89.14899 | 95.24863 | 82.38234 | 84.35503 | 84.56877 | 63.13354 | 67.97674 | 70.98237 | 73.51135 | 79.76388 | 81.66965 |
| 293 | neg_293 | Hydroxyproline | 259.551 | 280.9385 | 300.8627 | 230.1299 | 242.4251 | 244.148 | 232.1762 | 247.0936 | 263.5876 | 246.8083 | 270.2259 | 291.6875 |
| 294 | neg_294 | phosphonate | 2718.305 | 3008.643 | 3137.296 | 3023.547 | 3156.618 | 3410.764 | 2789.992 | 2914.744 | 3044.711 | 3359.108 | 3501.307 | 3662.678 |
| 295 | neg_295 | Saccharopine | 35.14922 | 35.54329 | 38.33286 | 5.565328 | 5.835773 | 6.261371 | 6.714277 | 7.152732 | 7.562947 | 14.52022 | 15.23029 | 15.70221 |
| 296 | neg_296 | Methylcitric acid | 608.1696 | 620.007 | 626.4312 | 1329.594 | 1456.837 | 1562.865 | 1349.264 | 1464.062 | 1557.843 | 1541.349 | 1546.904 | 1593.525 |
| 297 | neg_297 | L-aspartol adenylate | 2.860485 | 2.944623 | 3.092559 | 203.6821 | 204.9286 | 204.9565 | 6.339922 | 6.729494 | 7.150445 | 134.5158 | 146.1125 | 156.0604 |
| 298 | neg_298 | AMPPNP | 509.3668 | 523.4822 | 546.5217 | 539.304 | 541.6425 | 580.5036 | 524.1706 | 544.7582 | 581.0316 | 503.8432 | 546.5034 | 548.5846 |
| 299 | neg_299 | 1,2-Dihydroxy-3-keto-5-methylthiopentene | 0 | 0 | 0 | 88.19277 | 92.00759 | 97.48194 | 0.764873 | 0.836564 | 0.912069 | 124.9783 | 131.36 | 133.5643 |
| 300 | neg_300 | 4-Amino-2-methyl-5-phosphomethylpyrimidine | 3036.125 | 3190.609 | 3215.313 | 4.191652 | 4.334332 | 4.440814 | 9.334321 | 9.973456 | 10.62141 | 7.010186 | 7.554743 | 7.747395 |
| 301 | neg_301 | 3,4-Dihydroxymandelaldehyde | 0 | 0 | 0 | 35.03929 | 37.04204 | 39.7599 | 89.68919 | 93.62331 | 95.96191 | 64.19952 | 64.31961 | 64.79615 |
| 302 | neg_302 | 4-Phospho-D-erythronate | 251.8928 | 256.7674 | 267.4594 | 212.8863 | 216.4239 | 230.2901 | 243.2515 | 254.9414 | 276.4253 | 228.3715 | 240.8016 | 252.1072 |
| 303 | neg_303 | Deoxyuridine | 31.59245 | 34.09028 | 34.19244 | 1444.182 | 1491.646 | 1548.171 | 1109.219 | 1214.553 | 1227.845 | 1039.965 | 1094.635 | 1188.328 |
| 304 | neg_304 | N-Acetyl-glutamine | 51.544 | 52.24924 | 53.09484 | 56.6355 | 58.4631 | 63.2559 | 54.6108 | 58.33227 | 60.65411 | 47.01778 | 49.56268 | 54.26939 |
| 305 | neg_305 | NADH | 29.53218 | 30.50072 | 30.7785 | 81.38597 | 89.77305 | 95.35293 | 79.04857 | 83.62067 | 91.30929 | 71.40476 | 76.5461 | 77.26053 |
| 306 | neg_306 | PC(O-16:0/18:2(9Z,12Z)) | 31445.94 | 32163.28 | 34635.05 | 24385.36 | 25218.14 | 26905 | 24664.88 | 26601.73 | 27407.42 | 22710.77 | 24050.27 | 26014.2 |
| 307 | neg_307 | Malvidin 3-O-(cafeoylglucoside) | 1.409886 | 1.549558 | 1.59526 | 5.945699 | 6.138286 | 6.183831 | 4.843136 | 5.197412 | 5.35555 | 7.265147 | 7.907025 | 8.614559 |
| 308 | neg_308 | 5,6,7,8-tetrahydrofolyl-L-glutamic acid | 0 | 0 | 0 | 79.96399 | 82.01274 | 83.39343 | 58.30486 | 60.93602 | 66.44075 | 52.67676 | 56.66877 | 60.03902 |
| 309 | neg_309 | 2,5-Diamino-6-(5-phosphono)ribitylamino-4(3H)-pyrimidinone | 28.39176 | 28.84354 | 30.07978 | 20.43714 | 22.6123 | 24.70368 | 29.55997 | 29.69338 | 30.5627 | 21.07186 | 21.83749 | 22.27509 |
| 310 | neg_310 | erythro-4-hydroxy-L-glutamic acid | 50.14857 | 52.28838 | 53.15114 | 49.0762 | 51.31411 | 55.83761 | 56.97136 | 61.89499 | 67.6918 | 49.44766 | 52.47066 | 53.97371 |
| 311 | neg_311 | Dethiobiotin | 0.410464 | 0.425676 | 0.434926 | 11224.7 | 12173.48 | 12832.89 | 2544.163 | 2624.761 | 2828.871 | 10430.53 | 10964.29 | 11858.52 |
| 312 | neg_312 | lipol-AMP | 306.5225 | 314.8863 | 327.4937 | 350.9714 | 355.9348 | 375.148 | 980.9844 | 1084.98 | 1087.032 | 230.3985 | 243.5858 | 256.3752 |
| 313 | neg_313 | N-delta-(phosphonoacetyl)-L-ornithine | 866.8469 | 891.6 | 892.1605 | 1699.362 | 1866.042 | 1891.304 | 12622.49 | 12678 | 13285.53 | 649.1074 | 708.9167 | 713.4113 |
| 314 | neg_314 | Homocarnosine | 158.2671 | 169.9263 | 182.5298 | 104.4025 | 110.3693 | 117.4316 | 156.122 | 167.5329 | 180.8247 | 116.6331 | 129.0175 | 138.247 |

Continued Table S2

| 315 | neg_315 | Phosphoribosylformylglycineamidine | 299.784 | 315.902 | 345.6087 | 1453.793 | 1464.272 | 1598.891 | 1689.453 | 1872.796 | 1884.281 | 973.4501 | 1076.127 | 1107.51 |
| --- | --- | --- | --- | --- | --- | --- | --- | --- | --- | --- | --- | --- | --- | --- |
| 316 | neg_316 | 2-Oxo-3-hydroxy-4-phosphobutanoic acid | 161.473 | 175.4048 | 191.0841 | 158.771 | 168.5517 | 174.6867 | 163.6366 | 179.1636 | 195.0345 | 154.2084 | 161.6246 | 175.7473 |
| 317 | neg_317 | 4-Phosphopantothenoylcysteine | 194.1232 | 214.6771 | 218.3884 | 64.0283 | 65.3905 | 70.61695 | 2.928654 | 3.040624 | 3.158559 | 16.88248 | 17.16943 | 17.37521 |
| 318 | neg_318 | N-Succinyl-2-amino-6-ketopimelate | 14.35677 | 15.39053 | 16.58555 | 9853.134 | 10292.54 | 10670.93 | 10088.89 | 10640.86 | 11456.06 | 8050.115 | 8401.201 | 8646.345 |
| 319 | neg_319 | N6-(1,2-Dicarboxyethyl)-AMP | 131.0594 | 134.6706 | 139.4993 | 671.4827 | 740.748 | 802.64 | 528.4792 | 554.5702 | 564.5576 | 771.5443 | 818.4517 | 845.3674 |
| 320 | neg_320 | 3,4-dihydroxy-2-butanone-4-P | 622.5781 | 687.2044 | 709.8511 | 651.9076 | 682.4999 | 724.5887 | 668.0298 | 723.8336 | 767.486 | 696.9766 | 700.4958 | 760.5369 |
| 321 | neg_321 | 4-Pyridoxolactone | 166.8804 | 185.1703 | 188.9049 | 180.3061 | 184.4545 | 191.8071 | 177.1104 | 185.5266 | 185.9498 | 185.2231 | 191.6716 | 200.0064 |
| 322 | neg_322 | 3-Dehydroshikimic acid | 116196.2 | 117931.3 | 122133 | 2.277334 | 2.497785 | 2.658811 | 5.135808 | 5.591238 | 5.615994 | 2.335597 | 2.44119 | 2.599205 |
| 323 | neg_323 | L-Ribulose 5-phosphate | 120.0156 | 129.1868 | 140.3371 | 351.8019 | 357.4151 | 388.2772 | 630.5283 | 664.5099 | 703.4432 | 295.0225 | 297.0682 | 312.2554 |
| 324 | neg_324 | Cyanidin | 61.84591 | 63.27856 | 67.35622 | 81.00607 | 81.6127 | 81.90002 | 85.73183 | 85.84878 | 89.92984 | 78.30632 | 84.15228 | 89.82941 |
| 325 | neg_325 | Imidazole acetol-phosphate | 39.36374 | 39.65732 | 41.36625 | 37.37783 | 38.0106 | 39.31907 | 34.92652 | 38.12267 | 40.65593 | 37.25307 | 37.71101 | 39.88331 |
| 326 | neg_326 | 5-methyl-2-thiophenecarboxaldehyde | 55.47714 | 58.10606 | 62.75476 | 158.9672 | 169.0781 | 181.0443 | 86.75538 | 91.88299 | 98.76756 | 94.19484 | 94.65113 | 100.4461 |
| 327 | neg_327 | NAD | 0 | 0 | 0 | 25.30223 | 26.747 | 27.53499 | 35.34688 | 36.27363 | 38.37825 | 25.04735 | 26.85368 | 28.55039 |
| 328 | neg_328 | 3-Hydroxyisobutyric acid | 761.0979 | 804.3878 | 870.3806 | 88.16925 | 92.4173 | 101.0677 | 91.51777 | 95.45018 | 100.0486 | 113.6653 | 125.1233 | 129.3431 |
| 329 | neg_329 | 5'-n-propylthioadenosine | 680.6692 | 739.2822 | 780.926 | 17.93661 | 19.79451 | 20.13926 | 8.370419 | 8.544677 | 8.796754 | 4.965047 | 5.270545 | 5.499278 |
| 330 | neg_330 | Tetrahydropteridine | 48.98979 | 49.67468 | 54.55171 | 24.65947 | 27.0961 | 29.48417 | 560.5158 | 616.5497 | 677.4201 | 8.979184 | 9.677767 | 9.718405 |
| 331 | neg_331 | Protoanemonin | 199.3471 | 204.8122 | 209.7623 | 200.1908 | 203.5871 | 219.447 | 185.4456 | 205.9392 | 221.2253 | 199.4148 | 205.2582 | 210.3149 |
| 332 | neg_332 | Adenosine triphosphate | 395.2477 | 409.1358 | 427.8365 | 376.373 | 396.274 | 411.7099 | 417.9833 | 426.1305 | 431.7295 | 396.3397 | 405.5398 | 424.7634 |
| 333 | neg_333 | 5-L-Glutamyl-taurine | 0 | 0 | 0 | 6971.534 | 7242.316 | 7743.973 | 6081.076 | 6556.898 | 7020.772 | 5916.916 | 6398.979 | 6948.117 |
| 334 | neg_334 | meso-Tartaric acid | 133.3532 | 137.3999 | 145.3392 | 130.5918 | 137.5713 | 144.8357 | 132.3883 | 141.1713 | 147.0605 | 133.1665 | 133.98 | 135.024 |
| 335 | neg_335 | (2E)-2-(methoxycarbonylmethyl)but-2-enedioic acid | 486.4327 | 537.8715 | 590.8326 | 494.025 | 524.8861 | 570.8789 | 576.4023 | 592.9998 | 596.3096 | 546.4838 | 547.2941 | 582.8107 |
| 336 | neg_336 | 2-Succinylbenzoate | 111.3177 | 115.4378 | 116.7175 | 0 | 0 | 0 | 1.590625 | 1.609726 | 1.728711 | 0 | 0 | 0 |
| 337 | neg_337 | PE(18:1(11Z)/17:0) | 52.28301 | 54.17647 | 55.98122 | 38.30454 | 41.11795 | 42.00933 | 50.79685 | 54.58337 | 59.19973 | 20.9287 | 22.76425 | 23.08014 |
| 338 | neg_338 | Caprylic acid | 10006.19 | 10235.78 | 10239.09 | 12112.45 | 12831.37 | 13327.87 | 34069.82 | 35973.19 | 36513.08 | 9367.184 | 9798.301 | 10769.51 |
| 339 | neg_339 | 6-phospho-2-dehydro-D-gluconic acid | 90.88493 | 100.2 | 103.8894 | 93.50301 | 94.76359 | 100.6765 | 91.44278 | 92.03019 | 93.12029 | 83.35665 | 86.33278 | 90.16095 |

Continued Table S2

| 340 | neg_340 | (S)(+)-Allantoin | 55.92892 | 58.49951 | 60.70326 | 0 | 0 | 0 | 0 | 0 | 0 | 0 | 0 | 0 |
| --- | --- | --- | --- | --- | --- | --- | --- | --- | --- | --- | --- | --- | --- | --- |
| 341 | neg_341 | PS(18:0/0:0) | 0 | 0 | 0 | 0 | 0 | 0 | 203.789 | 210.7456 | 229.2955 | 0 | 0 | 0 |
| 342 | neg_342 | N-hydroxy DAP | 93.56477 | 94.7177 | 102.6657 | 90.33339 | 97.79042 | 106.2489 | 103.6131 | 110.6026 | 111.0872 | 105.9856 | 117.6599 | 127.0215 |
| 343 | neg_343 | Pyrazinamide | 2688.899 | 2898.588 | 3116.35 | 19.54833 | 21.38617 | 21.73733 | 1658.383 | 1664.006 | 1688.141 | 0 | 0 | 0 |
| 344 | neg_344 | 1-(Methylthio)-3-pentanone | 105.3039 | 116.9267 | 119.5726 | 17.62454 | 18.70212 | 19.83387 | 22.68667 | 22.9743 | 24.09127 | 17.84128 | 17.91698 | 18.18365 |
| 345 | neg_345 | 2-methyl-3-methylthiofuran | 25.25711 | 25.307 | 25.95986 | 24.78656 | 25.03275 | 26.14438 | 22.04779 | 23.295 | 24.67147 | 25.06071 | 26.1978 | 27.21966 |
| 346 | neg_346 | dGTP | 283.1041 | 298.4098 | 299.7968 | 241.948 | 268.3135 | 272.0226 | 314.7384 | 330.7461 | 347.429 | 274.2708 | 293.8892 | 311.6704 |
| 347 | neg_347 | L-Kynurenine | 595.6118 | 613.532 | 668.8633 | 0.95845 | 1.025774 | 1.101005 | 0.094286 | 0.104009 | 0.104312 | 11.38816 | 12.50413 | 13.43957 |
| 348 | neg_348 | 7-cyano-7-carbaguanine | 26.24793 | 28.16392 | 29.46929 | 0 | 0 | 0 | 0 | 0 | 0 | 0 | 0 | 0 |
| 349 | neg_349 | S-carbamylcysteine | 98.1332 | 108.7603 | 116.3623 | 92.24773 | 95.7676 | 96.91899 | 97.92684 | 101.0871 | 109.0573 | 100.0792 | 109.3076 | 117.2565 |
| 350 | neg_350 | serinamide | 127.5268 | 137.7746 | 149.8044 | 0 | 0 | 0 | 0 | 0 | 0 | 0 | 0 | 0 |
| 351 | neg_351 | 3-aminopropylphosphonate | 5.59742 | 6.084025 | 6.544036 | 30.83025 | 34.02671 | 35.25918 | 32.87558 | 33.16658 | 33.44067 | 27.91532 | 30.50811 | 30.6243 |
| 352 | neg_352 | 4-(2-Aminophenyl)-2,4-dioxobutanoic acid | 75.16671 | 81.94797 | 84.56056 | 85.15632 | 92.99215 | 99.27634 | 82.2173 | 87.80559 | 90.22851 | 94.09865 | 95.81223 | 99.18486 |
| 353 | neg_353 | S(8)-aminomethyldihydrolipoamide | 186.8188 | 195.8601 | 205.4856 | 279.118 | 289.0695 | 314.8702 | 251.5272 | 275.1703 | 294.8648 | 289.9931 | 303.0075 | 305.7104 |
| 354 | neg_354 | N-acetyl-L-glutamic acid | 152.8665 | 154.2573 | 155.0016 | 230.0917 | 239.4469 | 254.2589 | 237.2495 | 255.8941 | 266.4766 | 191.1331 | 203.5669 | 216.933 |
| 355 | neg_355 | L-gamma-Glutamyl-L-alanine | 65.36086 | 72.42964 | 73.52026 | 0 | 0 | 0 | 0 | 0 | 0 | 0 | 0 | 0 |
| 356 | neg_356 | Petunidin | 138.5407 | 151.3011 | 154.8507 | 18.0078 | 19.95162 | 21.1231 | 12.40994 | 12.88976 | 12.91901 | 13.39613 | 14.85023 | 14.98939 |
| 357 | neg_357 | 5-iodo-2'-dUMP | 47.91729 | 50.89839 | 53.46202 | 56.58468 | 59.55891 | 63.79661 | 49.96641 | 50.6898 | 51.7982 | 57.54129 | 63.08786 | 65.84802 |
| 358 | neg_358 | 2-Amino-3-phosphonopropionic acid | 0 | 0 | 0 | 4.379396 | 4.615527 | 5.015234 | 30.65202 | 30.78426 | 32.66694 | 0 | 0 | 0 |
| 359 | neg_359 | Melibiose | 71.68803 | 78.07894 | 78.5309 | 0.736715 | 0.748267 | 0.795597 | 0 | 0 | 0 | 0 | 0 | 0 |
| 360 | neg_360 | S-(2-aminoethyl)-L-cysteine | 19.70669 | 20.23551 | 20.43253 | 45.63967 | 49.48051 | 53.22358 | 56.16688 | 62.25725 | 66.24904 | 46.49023 | 47.20268 | 47.31592 |
| 361 | neg_361 | 5'-(dimethylsulfonio)-5'-deoxyadenosine | 381.0978 | 418.9653 | 429.6739 | 72.76633 | 77.41767 | 80.47305 | 289.2299 | 301.4911 | 331.3071 | 14.86918 | 16.42775 | 17.34351 |
| 362 | neg_362 | benzoin | 38.05737 | 39.62183 | 41.0765 | 101.486 | 110.8287 | 114.6688 | 97.72899 | 101.902 | 110.1533 | 100.5756 | 105.5607 | 111.2782 |
| 363 | neg_363 | dIMP | 194.7352 | 199.3654 | 206.3908 | 0 | 0 | 0 | 6.490291 | 6.894771 | 7.474097 | 0 | 0 | 0 |
| 364 | neg_364 | Trehalose 6-phosphate | 12.94879 | 13.34461 | 14.17159 | 517.6507 | 548.3959 | 570.3438 | 212.0299 | 230.195 | 234.7377 | 236.0593 | 246.7091 | 262.5054 |
| 365 | neg_365 | dihydro-10-thiopteroate | 101.2216 | 109.2925 | 112.0889 | 0 | 0 | 0 | 0 | 0 | 0 | 0 | 0 | 0 |

Continued Table S2

| 366 | neg_366 | CDP | 943.1598 | 955.5106 | 963.8374 | 940.9577 | 953.6486 | 1026.145 | 767.9355 | 851.5886 | 919.2753 | 818.2212 | 892.681 | 934.2862 |
| --- | --- | --- | --- | --- | --- | --- | --- | --- | --- | --- | --- | --- | --- | --- |
| 367 | neg_367 | thiolactomycin | 102.4714 | 112.8576 | 121.5948 | 3.202782 | 3.25509 | 3.426515 | 76.1433 | 77.23204 | 78.69729 | 1.86558 | 2.007714 | 2.154332 |
| 368 | neg_368 | FAD | 134.1422 | 144.2649 | 156.5755 | 215.4532 | 222.895 | 231.9177 | 147.6345 | 160.8376 | 164.1151 | 208.4007 | 218.1233 | 230.1517 |
| 369 | neg_369 | 2-Dehydro-D-gluconate | 179.5098 | 196.5338 | 205.891 | 4734.859 | 5044.083 | 5429.639 | 447.0461 | 492.6121 | 539.9312 | 4238.212 | 4308.432 | 4480.171 |
| 370 | neg_370 | Lipoamide | 0.826044 | 0.838844 | 0.862831 | 10.54086 | 10.62289 | 11.08444 | 41.90613 | 42.46847 | 43.28658 | 12.3513 | 12.52691 | 13.68023 |
| 371 | neg_371 | 2,3-Diketo-L-gulonate | 36.90034 | 40.34291 | 42.07927 | 32.98311 | 35.72605 | 38.67591 | 37.20484 | 40.168 | 43.75228 | 36.98702 | 39.42113 | 42.87101 |
| 372 | neg_372 | 2-methyl-3-methyldithiofuran | 82.98154 | 83.97569 | 89.24541 | 77.72852 | 79.20943 | 80.6807 | 77.64314 | 85.56985 | 87.83984 | 72.90919 | 79.6787 | 84.52512 |
| 373 | neg_373 | N-acetylmuramate(beta-methyl)-L-alanyl-D-glutamate | 38.489 | 40.07476 | 42.66 | 17.7948 | 18.02307 | 18.49698 | 15.7631 | 17.09597 | 17.16237 | 15.04598 | 16.37188 | 16.94347 |
| 374 | neg_374 | PhosphoribosylformiminoAICAR-phosphate | 0 | 0 | 0 | 23.06348 | 25.25425 | 25.30191 | 3.166832 | 3.303705 | 3.386573 | 25.83301 | 27.60265 | 27.8709 |
| 375 | neg_375 | 4,5-Dihydro-4-hydroxy-5-S-glutathionyl-benzo[a]pyrene | 1814.814 | 1864.069 | 1985.944 | 619.4922 | 632.1834 | 674.136 | 503.7349 | 511.931 | 534.4076 | 642.8678 | 696.7545 | 704.0443 |
| 376 | neg_376 | N2-Succinyl-L-glutamic acid 5-semialdehyde | 159.7238 | 160.6574 | 170.6629 | 1.872681 | 1.930701 | 2.107077 | 616.4713 | 665.0696 | 714.1017 | 4.090695 | 4.53298 | 4.664084 |
| 377 | neg_377 | PC(o-20:0/18:3(9Z,12Z,15Z)) | 61.01335 | 63.01121 | 63.02205 | 53.49301 | 58.37664 | 60.52837 | 65.15238 | 66.93954 | 72.50483 | 66.63911 | 69.98692 | 73.81328 |
| 378 | neg_378 | dcSAM | 5.384379 | 5.774458 | 5.799481 | 7.989705 | 8.426001 | 9.135212 | 4348.297 | 4647.024 | 4875.641 | 0 | 0 | 0 |
| 379 | neg_379 | 4,5-dihydroxyisophthalate | 61.96188 | 62.16656 | 68.32339 | 62.084 | 64.30409 | 69.03409 | 64.51786 | 65.56816 | 72.10289 | 53.12242 | 55.50512 | 56.12004 |
| 380 | neg_380 | L-Xylo-hexulonolactone | 42.3307 | 46.98731 | 49.18311 | 134.7284 | 136.1164 | 149.3368 | 116.7567 | 121.2712 | 133.3645 | 67.57281 | 72.8427 | 75.02728 |
| 381 | neg_381 | N2-Succinyl-L-arginine | 19.97713 | 20.22584 | 21.60535 | 49.94096 | 50.03246 | 54.08077 | 34.63542 | 37.0797 | 40.31886 | 41.31959 | 42.22504 | 43.48796 |
| 382 | neg_382 | N3-methylcytosine | 61.69553 | 61.86857 | 67.79219 | 91.82397 | 98.7299 | 103.2613 | 81.08743 | 83.04386 | 83.13035 | 75.34789 | 77.46573 | 80.96935 |
| 383 | neg_383 | PS(19:0/19:0) | 19.31336 | 19.97957 | 21.86917 | 13.94691 | 14.651 | 16.06642 | 13.27023 | 14.52253 | 15.70339 | 9.059739 | 9.14353 | 9.373597 |
| 384 | neg_384 | 2,3-pentane-dione | 321.7898 | 343.8613 | 370.9591 | 0 | 0 | 0 | 0 | 0 | 0 | 0 | 0 | 0 |
| 385 | neg_385 | 3-Carboxy-2,3,4,9-tetrahydro-1H-pyrido[3,4-b]indole-1-propanoic acid | 784.7109 | 854.8716 | 873.7004 | 884.3505 | 907.1477 | 945.8937 | 662.1346 | 666.5407 | 729.8417 | 319.8939 | 343.2025 | 344.6382 |
| 386 | neg_386 | N'-Formylkynurenine | 103.094 | 112.5652 | 117.3931 | 91.65648 | 98.82496 | 102.2428 | 103.7745 | 109.9301 | 111.0122 | 92.79809 | 103.0936 | 107.7592 |
| 387 | neg_387 | Hydroxymethylbilane | 459.0655 | 482.2099 | 511.0674 | 0 | 0 | 0 | 0 | 0 | 0 | 0 | 0 | 0 |
| 388 | neg_388 | Chorismate | 0 | 0 | 0 | 97.17034 | 99.142 | 100.1395 | 45.25321 | 50.15091 | 53.71804 | 104.9024 | 115.2288 | 123.4612 |

Continued Table S2

| 389 | neg_389 | D,L-alpha-methylphosphinothricin | 44.97192 | 48.96197 | 53.54609 | 0.059477 | 0.061393 | 0.061954 | 2.55843 | 2.747665 | 2.779734 | 0.070995 | 0.071748 | 0.076249 |
| --- | --- | --- | --- | --- | --- | --- | --- | --- | --- | --- | --- | --- | --- | --- |
| 390 | neg_390 | UDP-2,3-bis(3-hydroxytetradecanoyl)glucosamine | 45.93065 | 47.88933 | 48.38103 | 80.38553 | 82.3477 | 89.2124 | 38.1695 | 41.14437 | 43.02497 | 62.89563 | 64.10412 | 67.22413 |
| 391 | neg_391 | (2S)-2-Hydroxy-3-oxobutyl phosphate | 117.2211 | 129.8014 | 136.1334 | 120.5781 | 124.2984 | 127.7822 | 118.1806 | 119.9373 | 120.4877 | 100.4934 | 110.8516 | 113.0674 |
| 392 | neg_392 | Hypoxanthine | 9.455278 | 9.838917 | 10.15419 | 28.63294 | 30.55596 | 31.92031 | 34.60297 | 38.24372 | 38.40108 | 59.2572 | 63.24172 | 68.07605 |
| 393 | neg_393 | N-(5-Phospho-D-ribosyl)anthranilate | 9.758706 | 9.822257 | 10.4967 | 109.7013 | 110.9411 | 121.0337 | 132.5092 | 143.5864 | 153.4463 | 96.00429 | 104.2639 | 107.2393 |
| 394 | neg_394 | 5-(methylsulfanyl)-2,3-dioxopentyl phosphate | 34175.45 | 37710.44 | 38128.05 | 33002.49 | 35188.1 | 35996.15 | 36223.1 | 36274.42 | 36291.05 | 35361.13 | 35969.94 | 39485.55 |
| 395 | neg_395 | Oxalosuccinic acid | 381.8378 | 410.1863 | 433.4636 | 347.8124 | 370.1133 | 401.2068 | 407.3858 | 408.343 | 410.6301 | 405.1529 | 417.4089 | 434.8539 |
| 396 | neg_396 | S-tubercidinylhomocysteine | 35.68986 | 39.06479 | 41.31379 | 0 | 0 | 0 | 89.82595 | 96.99809 | 104.0168 | 2.045602 | 2.172969 | 2.173615 |
| 397 | neg_397 | Sorbic acid | 427.9954 | 456.4345 | 469.1728 | 429.4177 | 473.9732 | 508.0867 | 598.7501 | 661.631 | 669.3995 | 532.9899 | 538.8748 | 544.4632 |
| 398 | neg_398 | 5-Methylthioribose 1-phosphate | 231.7166 | 239.952 | 246.0847 | 224.16 | 229.6626 | 251.6781 | 212.0073 | 231.0112 | 235.2825 | 195.4636 | 200.9435 | 212.5837 |
| 399 | neg_399 | N-Acetylmuramic acid 6-phosphate | 163.4268 | 163.7637 | 171.8292 | 103.1143 | 105.6934 | 108.531 | 78.7937 | 79.42358 | 83.13504 | 100.8158 | 110.2968 | 118.869 |
| 400 | neg_400 | Thymine | 49.10379 | 53.67738 | 56.3188 | 0 | 0 | 0 | 0.608368 | 0.614402 | 0.630127 | 0.090701 | 0.094616 | 0.10229 |
| 401 | neg_401 | L-Tryptophan | 99.46576 | 107.5257 | 109.5904 | 1.001189 | 1.024284 | 1.112373 | 10.49049 | 10.92843 | 11.15716 | 0 | 0 | 0 |
| 402 | neg_402 | sinefungin | 0 | 0 | 0 | 12.19605 | 13.13155 | 14.2309 | 48.10391 | 50.3269 | 54.97414 | 6.287564 | 6.534931 | 6.935939 |
| 403 | neg_403 | Homoisocitric acid | 1862.651 | 1931.804 | 2009.483 | 2169.855 | 2193.546 | 2312.442 | 1786.221 | 1905.715 | 1914.508 | 2314.312 | 2443.806 | 2565.813 |
| 404 | neg_404 | indolmycin | 241.5029 | 247.6532 | 255.0661 | 255.503 | 258.4269 | 270.2235 | 247.6468 | 263.7217 | 272.1199 | 247.9066 | 251.4829 | 255.4626 |
| 405 | neg_405 | D-arabinonate | 8474.795 | 9092.225 | 9650.28 | 3642.232 | 3866.892 | 4047.551 | 3943.176 | 4355.052 | 4399.808 | 4914.376 | 5317.399 | 5743.907 |
| 406 | neg_406 | Itaconic acid | 46.86762 | 50.12864 | 51.91924 | 34.34359 | 35.79292 | 36.13744 | 32.03538 | 34.55707 | 38.00259 | 44.61277 | 48.47543 | 49.18742 |
| 407 | neg_407 | Acetic acid | 4.459122 | 4.924597 | 5.095657 | 12.71208 | 13.93802 | 15.08892 | 19.77145 | 20.74638 | 21.15561 | 23.8607 | 25.16916 | 27.03297 |
| 408 | neg_408 | N-Acetyl-L-glutamate 5-semialdehyde | 0 | 0 | 0 | 16.67829 | 17.45076 | 18.66519 | 1.014941 | 1.090374 | 1.150796 | 27.37361 | 27.83988 | 30.09431 |
| 409 | neg_409 | udp-galactose | 202.0744 | 221.1828 | 237.5477 | 207.483 | 211.0765 | 227.2238 | 258.1677 | 279.4278 | 279.5779 | 219.5226 | 225.3493 | 247.516 |
| 410 | neg_410 | Salicin | 172.2749 | 179.3105 | 187.7756 | 11.72529 | 12.84226 | 14.08282 | 12.17729 | 12.47441 | 13.70515 | 0 | 0 | 0 |
| 411 | neg_411 | Tetrahydrobiopterin | 559.3642 | 614.5212 | 643.7239 | 627.8316 | 637.3899 | 681.1558 | 561.6905 | 578.4493 | 594.2627 | 629.1163 | 660.9352 | 714.8887 |
| 412 | neg_412 | 2-Maleylacetate | 4264.29 | 4606.053 | 4821.499 | 6814.737 | 7329.965 | 7880.139 | 4962.633 | 5047.337 | 5379.9 | 6358.012 | 6911.19 | 7239.081 |
| 413 | neg_413 | Butyric acid | 103.2566 | 114.4412 | 124.7638 | 94.94021 | 100.8543 | 106.6737 | 90.9298 | 99.64659 | 104.9978 | 64.11757 | 70.30053 | 73.18598 |

Continued Table S2

| 414 | neg_414 | tetrahydrothiophene | 41.54189 | 41.89432 | 45.70455 | 333.8253 | 353.858 | 370.9342 | 155.8084 | 169.4287 | 170.738 | 196.7993 | 209.1572 | 228.3273 |
| --- | --- | --- | --- | --- | --- | --- | --- | --- | --- | --- | --- | --- | --- | --- |
| 415 | neg_415 | methyl-1,4-benzoquinone | 10.83787 | 11.55034 | 11.97968 | 9.124198 | 9.817153 | 9.963774 | 11.43425 | 12.15564 | 12.96092 | 9.423712 | 10.37749 | 11.15096 |
| 416 | neg_416 | N-Succinyl-L-glutamate | 81.95009 | 90.24134 | 95.31915 | 10.01369 | 10.98897 | 11.50874 | 6.468864 | 7.021965 | 7.470543 | 8.989885 | 9.434982 | 9.627247 |
| 417 | neg_417 | 3-(indol-3-yl)pyruvic acid | 27.46315 | 29.64629 | 31.98478 | 15.64465 | 16.37297 | 17.90831 | 14.72465 | 16.2918 | 16.68092 | 13.09347 | 13.87222 | 15.24682 |
| 418 | neg_418 | D-Mannonate | 10.50627 | 11.4012 | 11.70199 | 912.8214 | 958.4629 | 984.6531 | 36.71702 | 37.6569 | 39.53566 | 1322.551 | 1442.197 | 1544.213 |
| 419 | neg_419 | ppGp | 216.0491 | 232.671 | 243.8118 | 190.3709 | 196.3017 | 200.7192 | 208.2532 | 212.0656 | 220.8064 | 208.9863 | 210.913 | 214.1995 |
| 420 | neg_420 | Isopentenyl pyrophosphate | 121.965 | 132.3729 | 136.8184 | 110.8841 | 112.3656 | 115.9693 | 120.9614 | 124.2944 | 126.5168 | 124.1739 | 135.271 | 144.4185 |
| 421 | neg_421 | sn-Glycero-3-phosphocholine | 250.4962 | 277.3433 | 292.1964 | 4.619229 | 4.670145 | 4.956544 | 5.917558 | 6.171566 | 6.436491 | 4.405447 | 4.771855 | 4.914635 |
| 422 | neg_422 | phenyl-1-thio-beta-D-galactopyranoside | 37.37795 | 39.3433 | 42.39324 | 48.84997 | 53.56531 | 58.85689 | 70.94069 | 78.58265 | 85.64346 | 41.7096 | 45.49707 | 46.37158 |
| 423 | neg_423 | 3-Sulfinoalanine | 8.657099 | 8.727357 | 9.070706 | 40.60176 | 42.12536 | 45.55067 | 29.66885 | 31.66171 | 31.95313 | 94.61415 | 99.43608 | 108.8672 |
| 424 | neg_424 | 4-Carboxy-4-hydroxy-2-oxoadipate | 100.3861 | 111.3314 | 116.9302 | 99.1631 | 106.0061 | 115.7488 | 98.68393 | 109.642 | 116.5878 | 92.47462 | 95.91625 | 99.4955 |
| 425 | neg_425 | Cer 18:0;3/18:0;1 | 14623.1 | 15938.76 | 16189.05 | 13830.9 | 14558.08 | 15333.92 | 13643.46 | 15010.69 | 16336.98 | 14204.7 | 14883.72 | 16286.68 |
| 426 | neg_426 | PC(18:0/20:4(8Z,11Z,14Z,17Z)) | 2358.609 | 2372.844 | 2435.112 | 1756.22 | 1801.445 | 1914.4 | 1629.746 | 1634.41 | 1636.668 | 1110.641 | 1161.264 | 1161.773 |
| 427 | neg_427 | PC(18:3(9Z,12Z,15Z)/P-18:1(11Z)) | 19040.43 | 20453.63 | 21276.24 | 15867.58 | 16671.79 | 17213.98 | 16134.69 | 16344.99 | 16900.99 | 14088.82 | 15344.44 | 16249.18 |
| 428 | neg_428 | 2-Hydroxy-6-ketononatrienedioate | 54.12285 | 57.38103 | 59.51495 | 52.1234 | 52.88166 | 54.09288 | 51.31363 | 52.71961 | 52.73678 | 50.50664 | 51.49959 | 52.95699 |
| 429 | neg_429 | 2-Oxaloglutaric acid | 690.6817 | 707.1389 | 745.3147 | 631.2607 | 649.9731 | 657.5161 | 657.2946 | 715.6868 | 760.5002 | 636.0925 | 687.302 | 738.328 |
| 430 | neg_430 | Linoleic acid | 0.059976 | 0.063466 | 0.069664 | 0.922217 | 1.017207 | 1.079327 | 660.4021 | 686.2567 | 708.3662 | 0.509675 | 0.515373 | 0.529035 |
| 431 | neg_431 | Dihydroxyacetone phosphate acyl ester | 63.19969 | 66.09726 | 66.96297 | 59.62795 | 62.26177 | 63.02935 | 55.80851 | 58.26762 | 61.78179 | 56.11133 | 58.68075 | 60.46069 |
| 432 | neg_432 | Arbutin 6-phosphate | 0 | 0 | 0 | 0 | 0 | 0 | 0 | 0 | 0 | 0 | 0 | 0 |
| 433 | neg_433 | Xanthylic acid | 54.20342 | 56.7983 | 59.43422 | 42.65963 | 43.10987 | 43.47195 | 40.44539 | 42.01889 | 43.17579 | 41.41301 | 45.19212 | 47.33002 |
| 434 | neg_434 | UDP-N-acetylmuramoyl-L-alanyl-D-glutamate | 39.10514 | 39.77749 | 41.94104 | 41.25416 | 42.29437 | 43.21562 | 43.18524 | 44.67269 | 46.29554 | 44.56583 | 45.95676 | 47.15006 |
| 435 | neg_435 | 2-Isopropylmalic acid | 78.85438 | 82.03697 | 82.74434 | 309.6097 | 329.6037 | 333.5664 | 475.6123 | 481.2848 | 487.4884 | 103.5704 | 113.3428 | 121.2296 |
| 436 | neg_436 | Uroporphyrin III | 236.291 | 259.0758 | 274.7879 | 453.1264 | 454.6413 | 490.6705 | 471.2099 | 473.1256 | 487.258 | 412.0286 | 455.7526 | 472.0299 |
| 437 | neg_437 | 1-palmitoylglycerone 3-phosphate | 11.75606 | 12.40686 | 12.46347 | 455.2216 | 505.0803 | 539.7888 | 25.11157 | 25.20785 | 27.66915 | 2557.024 | 2709.594 | 2872.422 |
| 438 | neg_438 | Geranyl-PP | 55.36522 | 55.91862 | 56.78789 | 53.99521 | 56.44869 | 60.13501 | 54.65018 | 56.23809 | 61.51243 | 50.96542 | 53.54943 | 54.64794 |

Continued Table S2

| 439 | neg_439 | 2'-(5-triphosphoribosyl)-3'-dephospho-CoA | 2052.918 | 2063.551 | 2132.92 | 1868.604 | 2069.11 | 2225.845 | 1940.175 | 2126.186 | 2309.334 | 1949.457 | 2083.333 | 2235.429 |
| --- | --- | --- | --- | --- | --- | --- | --- | --- | --- | --- | --- | --- | --- | --- |
| 440 | neg_440 | Pyridoxal | 551.7792 | 566.4742 | 589.93 | 549.794 | 575.926 | 596.9653 | 516.4304 | 561.3111 | 573.5876 | 620.503 | 634.8329 | 664.82 |
| 441 | neg_441 | Orotic acid | 30.3018 | 33.14575 | 34.2152 | 34.48223 | 34.57806 | 34.71603 | 32.4257 | 33.12938 | 35.11402 | 31.95857 | 35.28415 | 35.64451 |
| 442 | neg_442 | 2,3-dimercaptopropan-1-ol | 37.38144 | 38.59288 | 39.0046 | 33.63586 | 36.56042 | 38.3346 | 37.54777 | 39.48886 | 39.66407 | 34.34669 | 36.36512 | 38.48838 |
| 443 | neg_443 | 5-Amino-6-(5'-phosphoribosylamino)uracil | 12.1777 | 12.71972 | 13.0993 | 11.32476 | 12.23244 | 12.54879 | 4.679929 | 5.103102 | 5.417602 | 7.824715 | 7.874517 | 8.558183 |
| 444 | neg_444 | 4-oxo-N-acetylneuraminate | 7.416988 | 7.893463 | 8.624728 | 111.2392 | 116.2865 | 117.2569 | 72.03046 | 74.30127 | 75.83364 | 196.2672 | 204.7142 | 222.3141 |
| 445 | neg_445 | dTDP-D-fucosamine | 685.4175 | 735.4275 | 794.355 | 120.9648 | 123.4481 | 133.5534 | 53.81763 | 57.00189 | 57.3536 | 127.3375 | 132.0242 | 143.4795 |
| 446 | neg_446 | 4-Vinylphenol | 29.76709 | 31.36774 | 33.89064 | 122.5179 | 134.7267 | 137.9193 | 71.80747 | 72.7894 | 74.93351 | 848.2611 | 915.8116 | 926.9646 |
| 447 | neg_447 | O-Phosphohomoserine | 16.10062 | 16.83866 | 18.44919 | 0 | 0 | 0 | 7.031914 | 7.339545 | 8.0475 | 0 | 0 | 0 |
| 448 | neg_448 | hydroxyl diethyl succinate | 153.8102 | 164.9137 | 165.9643 | 176.6654 | 186.0074 | 193.1623 | 170.8424 | 189.3267 | 204.0356 | 183.4264 | 192.6673 | 196.6021 |
| 449 | neg_449 | 3-mercaptopropionate | 36.30331 | 40.28685 | 43.28818 | 38.56138 | 41.21355 | 41.8415 | 40.52918 | 40.77564 | 42.78452 | 32.12394 | 32.74476 | 34.11039 |
| 450 | neg_450 | UDP-D-glucose | 56.48108 | 56.62637 | 58.59772 | 27.70197 | 28.22557 | 30.70796 | 56.06645 | 58.68282 | 62.7976 | 46.06736 | 47.50216 | 51.77312 |
| 451 | neg_451 | 3-Oxo-alpha-ionol | 47.71374 | 49.81969 | 52.31207 | 60.32915 | 62.6823 | 67.59 | 63.16946 | 64.68441 | 66.05462 | 58.49587 | 59.3587 | 64.18879 |
| 452 | neg_452 | N1-methyladenine | 39.23438 | 43.57337 | 45.33914 | 47.03784 | 49.11612 | 51.35507 | 51.18323 | 52.9315 | 55.03312 | 69.15411 | 74.57818 | 74.8757 |
| 453 | neg_453 | decoyinine | 3527.674 | 3867.426 | 3957.437 | 5419.015 | 5952.829 | 6284.978 | 6307.034 | 6558.649 | 7040.691 | 5184.045 | 5634.996 | 6005.091 |
| 454 | neg_454 | methylphosphonate | 45.71399 | 47.63286 | 49.14893 | 38.39501 | 41.28187 | 41.49627 | 41.05617 | 44.92586 | 47.50316 | 43.50204 | 48.12218 | 52.48191 |
| 455 | neg_455 | 3-Oxo-beta-ionone | 2.596205 | 2.720297 | 2.725855 | 3.423916 | 3.441112 | 3.709558 | 468.308 | 471.184 | 478.9915 | 1.390581 | 1.450182 | 1.584931 |
| 456 | neg_456 | Dyspropterin | 27.30449 | 28.09833 | 29.07172 | 33.54824 | 35.85359 | 39.0304 | 36.9045 | 37.98527 | 39.44009 | 37.49523 | 38.6939 | 40.14354 |
| 457 | neg_457 | (S)-3-Hydroxyisobutyryl-CoA | 0 | 0 | 0 | 17.43519 | 17.78063 | 17.94174 | 14.96337 | 16.25005 | 16.57168 | 6.450267 | 7.079377 | 7.700626 |
| 458 | neg_458 | D-idarate | 28.3186 | 30.70709 | 31.97816 | 26.46994 | 28.5517 | 29.29425 | 24.78442 | 25.07346 | 26.26455 | 24.66021 | 25.7232 | 27.67138 |
| 459 | neg_459 | Ascorbic acid | 24.33963 | 25.70418 | 27.74656 | 21.8275 | 23.60246 | 24.24673 | 25.87954 | 27.70885 | 29.26189 | 23.18257 | 25.56029 | 25.69768 |
| 460 | neg_460 | L-Xylulose 1-phosphate | 48.70579 | 50.5738 | 51.6797 | 44.0258 | 47.03601 | 49.57671 | 51.36044 | 56.68088 | 58.65907 | 45.67523 | 50.68364 | 54.85597 |
| 461 | neg_461 | FADH | 1.627437 | 1.80249 | 1.845356 | 28.26525 | 31.0957 | 32.06772 | 4.313469 | 4.375589 | 4.501392 | 19.08776 | 20.40654 | 21.10291 |
| 462 | neg_462 | S-Methyl-L-methionine | 158.873 | 162.9648 | 172.8257 | 173.2229 | 183.4609 | 192.5471 | 258.126 | 269.9563 | 287.184 | 210.2384 | 221.7982 | 243.3331 |
| 463 | neg_463 | 5'-methylthioformycin | 2647.708 | 2792.327 | 2796.389 | 3143.958 | 3294.08 | 3436.403 | 3001.162 | 3216.021 | 3429.842 | 2952.306 | 3249.707 | 3367.499 |
| 464 | neg_464 | Glucosamine-1P | 1.79606 | 1.847581 | 2.016083 | 64.49927 | 71.37441 | 74.33634 | 81.75403 | 81.92325 | 89.97762 | 45.97833 | 49.31792 | 49.37373 |

Continued Table S2

| 465 | neg_465 | Vanillyl acetate | 0 | 0 | 0 | 78.96478 | 86.35955 | 88.7871 | 24.03347 | 26.19451 | 27.00753 | 52.46367 | 52.48738 | 56.70721 |
| --- | --- | --- | --- | --- | --- | --- | --- | --- | --- | --- | --- | --- | --- | --- |
| 466 | neg_466 | nicotinic acid D-ribonucleotide | 25.95088 | 27.67017 | 28.7933 | 0 | 0 | 0 | 0 | 0 | 0 | 0 | 0 | 0 |
| 467 | neg_467 | CDPglucose | 107.6175 | 115.1648 | 115.9915 | 111.2938 | 120.5307 | 121.9227 | 105.1538 | 107.4931 | 117.6996 | 109.8004 | 111.6499 | 114.1259 |
| 468 | neg_468 | dATP | 416.2112 | 433.1215 | 468.5954 | 394.642 | 424.6536 | 446.3666 | 543.8069 | 547.5719 | 566.5813 | 455.856 | 468.6458 | 504.4607 |
| 469 | neg_469 | Octanol | 7.755157 | 8.234191 | 8.867069 | 5.690415 | 5.957836 | 6.523179 | 122.0328 | 134.1476 | 139.6078 | 3.876321 | 4.06492 | 4.465128 |
| 470 | neg_470 | Limonene | 128.6374 | 128.9402 | 139.7037 | 194.0697 | 196.4546 | 197.3266 | 223.8796 | 233.726 | 233.7896 | 222.2742 | 237.6024 | 252.2449 |
| 471 | neg_471 | Pyridoxal 5'-phosphate | 0 | 0 | 0 | 7.12424 | 7.212156 | 7.683123 | 5.144873 | 5.240464 | 5.578008 | 10.39692 | 10.56015 | 10.73113 |
| 472 | neg_472 | Phenyl acetate | 19.13879 | 20.02633 | 20.9147 | 0 | 0 | 0 | 1.833546 | 1.95473 | 2.132934 | 0 | 0 | 0 |
| 473 | neg_473 | Tartronate semialdehyde | 7856.185 | 7997.731 | 8467.001 | 7445.368 | 7506.261 | 8146.42 | 7565.813 | 7750.819 | 7761.789 | 7740.096 | 7769.284 | 8422.131 |
| 474 | neg_474 | Allysine | 45.58274 | 47.51171 | 51.52805 | 50.9667 | 55.24123 | 58.69185 | 70.99787 | 77.44863 | 83.89794 | 41.17524 | 43.15009 | 44.63037 |
| 475 | neg_475 | Theaflavin | 1595.693 | 1713.488 | 1735.983 | 0 | 0 | 0 | 0 | 0 | 0 | 0 | 0 | 0 |
| 476 | neg_476 | Apiin | 26926.1 | 28429.88 | 29551.44 | 16013.13 | 16682.18 | 17492.61 | 14217.85 | 15785.2 | 16817.73 | 12755.49 | 13005.03 | 13073.82 |
| 477 | neg_477 | Apigenin 7-glucoside | 3241.34 | 3397.098 | 3578.877 | 7390.701 | 8130.008 | 8271.593 | 4352.995 | 4666.626 | 4826.812 | 4956.794 | 5159.886 | 5499.68 |
| 478 | neg_478 | Quercitrin | 20242.79 | 21724.97 | 22383.38 | 224.421 | 233.5572 | 247.4119 | 128.751 | 138.819 | 148.6827 | 163.6286 | 173.16 | 185.066 |
| 479 | neg_479 | Sterigmatocystin | 0.555703 | 0.582144 | 0.599333 | 397.3911 | 401.6664 | 402.4347 | 41.56886 | 45.15195 | 47.64718 | 152.7012 | 162.5053 | 169.7819 |
| 480 | neg_480 | 12-Oxo-2,3-dinor-10,15-phytodienoic acid | 0 | 0 | 0 | 0 | 0 | 0 | 296.4428 | 327.3578 | 345.0998 | 0 | 0 | 0 |
| 481 | neg_481 | 13,14-Dihydro-15-keto-PGE2 | 997.1472 | 1042.871 | 1126.247 | 1129.701 | 1242.379 | 1294.29 | 159155.1 | 175113.4 | 183528.5 | 961.1321 | 1011.704 | 1066.527 |
| 482 | neg_482 | Chlorogenate | 6512 | 6857.542 | 7522.674 | 17.26269 | 18.22697 | 19.03746 | 439.4077 | 442.0698 | 477.0123 | 17.23976 | 17.38076 | 18.56267 |
| 483 | neg_483 | Alternariol | 1.001534 | 1.11278 | 1.158392 | 3.19991 | 3.506063 | 3.790787 | 0 | 0 | 0 | 1.195674 | 1.211753 | 1.252513 |
| 484 | neg_484 | Serinyl-Histidine | 5.503695 | 6.04006 | 6.369562 | 12.04548 | 12.35442 | 12.36698 | 215.4898 | 220.3069 | 233.7632 | 10.00277 | 10.13821 | 11.0699 |
| 485 | neg_485 | N2,N2-Dimethylguanosine | 0 | 0 | 0 | 175.9068 | 177.5236 | 181.5861 | 187.5056 | 205.503 | 218.1918 | 243.6387 | 248.5265 | 250.7055 |
| 486 | neg_486 | 2-Thiocytidine | 176.2818 | 178.5185 | 195.8196 | 5.05334 | 5.191766 | 5.224447 | 62.02226 | 63.40148 | 67.4617 | 0.932026 | 1.010709 | 1.074409 |
| 487 | neg_487 | Threoninyl-Glutamine | 54.95631 | 55.65739 | 59.41025 | 46.67972 | 49.69552 | 51.98863 | 50.39844 | 55.03737 | 55.80939 | 44.84468 | 46.77642 | 50.64165 |
| 488 | neg_488 | Ononin | 387.2788 | 402.1993 | 422.3329 | 3.552092 | 3.687634 | 3.976419 | 1.667247 | 1.852141 | 1.965498 | 0 | 0 | 0 |
| 489 | neg_489 | 1,3-Dicaffeoylquinic acid | 9.370951 | 10.35829 | 10.6129 | 43.61575 | 43.62523 | 44.19755 | 38.86122 | 41.74134 | 45.0456 | 16.33274 | 17.49919 | 18.38054 |
| 490 | neg_490 | Deoxyinosine | 342.703 | 350.95 | 359.4857 | 0 | 0 | 0 | 4.22101 | 4.675185 | 5.098948 | 0 | 0 | 0 |

Continued Table S2

| 491 | neg_491 | Floxuridine | 87.40192 | 93.18572 | 95.34395 | 12.16319 | 13.07357 | 13.90738 | 28.95002 | 30.98491 | 32.11971 | 3.736615 | 3.954139 | 4.211418 |
| --- | --- | --- | --- | --- | --- | --- | --- | --- | --- | --- | --- | --- | --- | --- |
| 492 | neg_492 | (-)-Catechin 3-O-gallate | 2399.41 | 2411.68 | 2438.79 | 2.469358 | 2.531801 | 2.653123 | 2.006833 | 2.15587 | 2.293675 | 0 | 0 | 0 |
| 493 | neg_493 | Budesonide | 0 | 0 | 0 | 6.287824 | 6.359596 | 6.530237 | 8090.811 | 8193.626 | 8485.832 | 3.66516 | 3.861118 | 3.990185 |
| 494 | neg_494 | Kaempferol 3-O-rutinoside | 15723.91 | 16956.97 | 17091.97 | 4819.271 | 4935.202 | 5099.775 | 5098.066 | 5451.245 | 5830.784 | 3168.991 | 3178.606 | 3344.933 |
| 495 | neg_495 | 5-Hydroxy-L-tryptophan | 5.022115 | 5.052569 | 5.168448 | 126.9506 | 140.4743 | 141.285 | 151.3397 | 163.2985 | 179.2745 | 171.0736 | 171.2436 | 178.4018 |
| 496 | neg_496 | Apigenin 7-O-neohesperidoside | 22282.57 | 23612.82 | 25909.02 | 1529.305 | 1678.094 | 1722.692 | 3842.358 | 3938.739 | 4247.32 | 4670.177 | 4892.06 | 5255.455 |
| 497 | neg_497 | Dexpanthenol | 6.479484 | 7.199408 | 7.207597 | 20.84033 | 21.37532 | 21.53615 | 6390.444 | 6584.543 | 6714.916 | 9.907948 | 10.83809 | 11.75242 |
| 498 | neg_498 | Rutin | 176.3494 | 194.1591 | 197.7726 | 118.6677 | 125.548 | 125.559 | 119.4431 | 124.3664 | 131.4125 | 113.1995 | 122.8793 | 122.9296 |
| 499 | neg_499 | Biopterin | 8.99217 | 9.39419 | 10.08664 | 52.95785 | 53.05887 | 56.27545 | 25.12854 | 26.84799 | 27.07624 | 33.92625 | 36.9293 | 39.31572 |
| 500 | neg_500 | Mimosine | 46.15181 | 49.63679 | 51.21531 | 62.41239 | 62.81217 | 68.70621 | 58.80778 | 64.25776 | 70.01178 | 73.18527 | 74.32094 | 77.3757 |
| 501 | neg_501 | Helenalin | 91.8128 | 93.66883 | 95.50037 | 80.24721 | 82.53654 | 87.85733 | 77.38786 | 78.35534 | 85.08372 | 78.12669 | 79.65665 | 86.5675 |
| 502 | neg_502 | Ethyl -D-glucuronide | 2188.83 | 2344.048 | 2365.764 | 2153.8 | 2263.983 | 2313.879 | 1653.411 | 1769.245 | 1881.548 | 2531.058 | 2703.344 | 2787.111 |
| 503 | neg_503 | Teniposide | 191.9657 | 210.1669 | 223.4592 | 207.0494 | 210.972 | 229.5082 | 219.508 | 230.6947 | 248.051 | 197.7308 | 213.4783 | 214.2213 |
| 504 | neg_504 | Ascorbic acid 6-palmitate | 12.02167 | 13.06224 | 13.99496 | 11.40093 | 12.40984 | 12.72781 | 3911.799 | 4155.227 | 4414.763 | 16.97193 | 17.84513 | 19.16601 |
| 505 | neg_505 | Bilobalide | 104.9034 | 113.2409 | 113.7505 | 50.37278 | 51.51402 | 54.25723 | 108.7814 | 117.124 | 120.1135 | 11.0181 | 11.3178 | 12.07885 |
| 506 | neg_506 | Asparaginyl-Alanine | 1504.866 | 1533.75 | 1626.377 | 0 | 0 | 0 | 28.2617 | 29.52369 | 29.79613 | 0 | 0 | 0 |
| 507 | neg_507 | Procyanidin A1 | 114.5816 | 126.4176 | 134.0855 | 736.1278 | 789.9522 | 828.9939 | 2659.782 | 2718.103 | 2861.244 | 31.45088 | 32.32598 | 33.76587 |
| 508 | neg_508 | Quinate | 167495.3 | 167626.8 | 169641.9 | 1602.886 | 1615.062 | 1684.25 | 1335.063 | 1374.001 | 1451.065 | 1547.083 | 1560.504 | 1634.162 |
| 509 | neg_509 | 4-Hydroxy-2H-pyran-3-carboxaldehyde | 57.47845 | 58.36445 | 60.27864 | 0 | 0 | 0 | 0 | 0 | 0 | 0 | 0 | 0 |
| 510 | neg_510 | 5'-Phosphoribosyl-5-amino-4-imidazolecarboxamide (AICAR) | 10.455 | 11.36178 | 11.77428 | 32.40635 | 35.98388 | 38.70511 | 29.10064 | 32.08849 | 34.29041 | 38.42317 | 39.30244 | 42.02472 |
| 511 | neg_511 | 4-thiouridine | 9.750793 | 10.59824 | 11.05797 | 430.5817 | 442.1449 | 470.1483 | 444.7983 | 464.9413 | 497.2056 | 319.1933 | 338.6255 | 351.6407 |
| 512 | neg_512 | Primidone | 0 | 0 | 0 | 0 | 0 | 0 | 835.9506 | 863.7423 | 895.9022 | 0 | 0 | 0 |
| 513 | neg_513 | Daidzin | 52.97344 | 57.82643 | 60.4716 | 38.02615 | 42.1877 | 42.6013 | 33.36409 | 35.25117 | 37.99438 | 41.69867 | 42.0631 | 43.36634 |
| 514 | neg_514 | Chorismic acid | 176.7193 | 181.4943 | 194.8234 | 0 | 0 | 0 | 0 | 0 | 0 | 0 | 0 | 0 |
| 515 | neg_515 | Gemcitabine | 11.43692 | 12.27416 | 13.31888 | 69.05422 | 73.41176 | 73.82209 | 47.26069 | 49.99544 | 54.95532 | 74.84683 | 82.98863 | 86.67055 |

Continued Table S2

| 516 | neg_516 | Daunorubicin | 79.54941 | 83.90058 | 91.7835 | 0 | 0 | 0 | 0 | 0 | 0 | 0 | 0 | 0 |
| --- | --- | --- | --- | --- | --- | --- | --- | --- | --- | --- | --- | --- | --- | --- |
| 517 | neg_517 | Sinapyl aldehyde | 25.11115 | 25.47904 | 25.53257 | 29.41665 | 31.81492 | 33.48312 | 33.38525 | 35.40258 | 38.52597 | 42.75232 | 45.33405 | 49.27507 |
| 518 | neg_518 | Bergenin | 93.35779 | 98.58644 | 104.886 | 1.61677 | 1.720933 | 1.848266 | 0.906484 | 0.951635 | 0.982674 | 0.56195 | 0.563503 | 0.599899 |
| 519 | neg_519 | N-Formylmethionine | 0 | 0 | 0 | 306.6881 | 321.533 | 337.3976 | 286.3741 | 297.9361 | 305.4616 | 248.5276 | 262.9016 | 263.6052 |
| 520 | neg_520 | Naringin | 86.32957 | 89.01462 | 97.72686 | 2636.944 | 2749.281 | 2989.532 | 4088.775 | 4533.854 | 4616.226 | 1208.272 | 1301.709 | 1422.223 |
| 521 | neg_521 | Baicalin | 57.83403 | 60.73988 | 64.48329 | 6.321831 | 6.941692 | 7.034115 | 0 | 0 | 0 | 5.363892 | 5.682659 | 5.695177 |
| 522 | neg_522 | 6-Keto-PGF1a | 82.22834 | 86.91861 | 88.58349 | 89.09464 | 96.21155 | 103.3752 | 6728.056 | 7052.497 | 7201.666 | 71.64184 | 76.1157 | 78.46845 |
| 523 | neg_523 | 1,3,5(10)-Estratrien-3,17.beta.-diol 17-glucosiduronate | 7786.825 | 8525.051 | 9128.171 | 7904.345 | 8688.564 | 8713.927 | 7776.774 | 8177.116 | 8332.769 | 8927.777 | 9090.006 | 9517.086 |
| 524 | neg_524 | Hygromycin B | 42.36269 | 44.98549 | 48.77506 | 36.79421 | 37.12264 | 38.57887 | 41.13576 | 42.36212 | 45.34866 | 33.12791 | 36.33718 | 37.15295 |
| 525 | neg_525 | 6''-O-Malonyldaidzin | 676.0599 | 716.4661 | 737.2176 | 513.9336 | 518.7476 | 534.6485 | 783.0255 | 828.2283 | 904.2441 | 285.3544 | 297.8535 | 311.6859 |
| 526 | neg_526 | 5,7-Dihydroxyflavone | 1346.942 | 1425.647 | 1495.886 | 5305.41 | 5686.326 | 5976.36 | 7016.327 | 7587.931 | 7788.225 | 3859.722 | 4058.063 | 4210.197 |
| 527 | neg_527 | 16-hydroxy hexadecanoic acid | 119.2709 | 131.101 | 142.1317 | 135.6855 | 139.9227 | 144.0066 | 4683.348 | 5096.698 | 5473.343 | 109.1405 | 117.8464 | 121.4992 |
| 528 | neg_528 | Cysteinyl-Alanine | 1413.239 | 1414.907 | 1533.614 | 1.197689 | 1.280374 | 1.348321 | 4.525303 | 4.684307 | 4.798302 | 1.128007 | 1.24107 | 1.29667 |
| 529 | neg_529 | Ginkgetin | 10.60313 | 11.40586 | 11.42327 | 36.86495 | 39.93122 | 39.9929 | 69.01823 | 71.34682 | 73.99481 | 9.410567 | 9.766349 | 9.977231 |
| 530 | neg_530 | Epigallocatechin gallate | 259778.3 | 286420.5 | 312605.6 | 482.2948 | 502.9398 | 548.7148 | 410.5226 | 454.1096 | 456.67 | 414.0139 | 452.4063 | 469.9478 |
| 531 | neg_531 | Glycitin | 8.930167 | 9.878271 | 10.24009 | 4.59491 | 4.918902 | 5.028795 | 51.9532 | 53.3562 | 53.83025 | 0 | 0 | 0 |
| 532 | neg_532 | Narcissin | 23.92968 | 25.30519 | 27.31035 | 894.4094 | 939.3138 | 969.7215 | 617.7839 | 662.6304 | 685.9145 | 1065.323 | 1175.789 | 1195.512 |
| 533 | neg_533 | Baicalein | 41.27663 | 44.85423 | 45.85691 | 0 | 0 | 0 | 0 | 0 | 0 | 0 | 0 | 0 |
| 534 | neg_534 | Tetracenomycin F2 | 0 | 0 | 0 | 12.1855 | 12.8407 | 13.47836 | 4.774769 | 5.181778 | 5.537047 | 2.23493 | 2.42971 | 2.558518 |
| 535 | neg_535 | (Glutaraldehyde) | 77.15503 | 77.65192 | 82.23147 | 0 | 0 | 0 | 0 | 0 | 0 | 0 | 0 | 0 |
| 536 | neg_536 | 2'-O-methylinosine | 29.32055 | 31.21026 | 33.66383 | 0.421958 | 0.445564 | 0.475846 | 24.40745 | 25.97272 | 27.35293 | 0 | 0 | 0 |
| 537 | neg_537 | MCI-186 | 99.70707 | 99.79132 | 103.7278 | 225.501 | 241.571 | 243.4609 | 220.1373 | 226.0646 | 242.3086 | 243.0941 | 255.4225 | 266.8018 |
| 538 | neg_538 | 3-(2-Hydroxyphenyl)propionic acid | 16.48432 | 17.05289 | 17.30264 | 24.28419 | 25.43672 | 25.96242 | 28.84286 | 31.2351 | 32.50055 | 34.56947 | 34.81085 | 35.22917 |
| 539 | neg_539 | 8-hydroxy Guanosine | 6.993991 | 7.770248 | 8.41204 | 19.01968 | 19.38057 | 20.97562 | 22.57654 | 24.96084 | 26.52059 | 16.2182 | 16.35094 | 16.51692 |
| 540 | neg_540 | Aspartyl-Tyrosine | 185.8847 | 193.3331 | 195.1795 | 0 | 0 | 0 | 0 | 0 | 0 | 0 | 0 | 0 |

Continued Table S2

| 541 | neg_541 | 2-Deoxy-D-glucose 6-phosphate | 209.9588 | 220.0719 | 228.1818 | 292.3055 | 294.3102 | 319.5045 | 231.6476 | 256.6891 | 272.5136 | 291.2871 | 298.1008 | 317.1886 |
| --- | --- | --- | --- | --- | --- | --- | --- | --- | --- | --- | --- | --- | --- | --- |
| 542 | neg_542 | Doxycycline | 12.64217 | 13.86237 | 14.75144 | 34.09738 | 34.21785 | 36.30001 | 16.2416 | 17.83236 | 18.68316 | 35.81828 | 39.72003 | 40.2691 |
| 543 | neg_543 | PHLORACETOPHENONE | 51.59057 | 53.9696 | 55.34938 | 0 | 0 | 0 | 0 | 0 | 0 | 0 | 0 | 0 |
| 544 | neg_544 | 4-Androsten-17.beta.-ol-3-one glucosiduronate | 153.4142 | 166.1966 | 181.7512 | 120.3966 | 124.0898 | 128.8584 | 110.6657 | 122.9026 | 128.6979 | 117.1934 | 122.5527 | 126.4833 |
| 545 | neg_545 | .alpha.-D-(+)-Talose | 4433.379 | 4908.824 | 5298.881 | 707.5082 | 720.5073 | 780.6285 | 645.7982 | 686.7306 | 700.919 | 1034.152 | 1108.818 | 1153.625 |
| 546 | neg_546 | Gentisaldehyde | 136.4753 | 140.7559 | 150.6227 | 8.919088 | 9.15312 | 9.80171 | 15.53066 | 16.11042 | 17.08635 | 10.12498 | 10.52624 | 11.34821 |
| 547 | neg_547 | Astragalin | 0 | 0 | 0 | 63.73658 | 66.81869 | 69.77687 | 0 | 0 | 0 | 76.64938 | 77.11899 | 80.74892 |
| 548 | neg_548 | Sulindac | 0 | 0 | 0 | 42.27788 | 44.76261 | 46.63955 | 0 | 0 | 0 | 33.08174 | 36.46541 | 39.7961 |
| 549 | neg_549 | Diosmetin | 61.34593 | 67.01163 | 69.79605 | 22.42033 | 23.92864 | 25.89445 | 24.05275 | 24.47689 | 25.50679 | 19.76412 | 21.13716 | 22.02276 |
| 550 | neg_550 | Demethoxycurcumin | 50.20115 | 50.78527 | 54.97252 | 38.2982 | 41.27568 | 43.48557 | 40.17146 | 42.06923 | 42.89196 | 34.87934 | 37.94861 | 41.72231 |
| 551 | neg_551 | Tryptophanol | 37.34989 | 38.4982 | 41.5055 | 38.88488 | 40.66287 | 44.0459 | 47.69099 | 48.23791 | 50.03368 | 33.71627 | 36.037 | 39.57351 |
| 552 | neg_552 | Prolyl-Tryptophan | 44.13038 | 47.0115 | 48.9959 | 69.39169 | 73.82109 | 77.39927 | 80.03885 | 84.44979 | 88.693 | 61.89629 | 66.40065 | 68.95549 |
| 553 | neg_553 | 17-Beta-Estradiol-3,17-beta-sulfate | 850.7247 | 875.9434 | 892.0529 | 1164.753 | 1272.275 | 1363.91 | 1246.336 | 1339.581 | 1444.397 | 857.3634 | 919.6621 | 997.0948 |
| 554 | neg_554 | Clopidogrel | 58.41993 | 62.13366 | 63.90254 | 1818.269 | 1896.605 | 1906.907 | 1793.312 | 1896.319 | 2050.657 | 1661.396 | 1820.941 | 1856.368 |
| 555 | neg_555 | Methacycline | 30.09861 | 31.004 | 33.05205 | 0 | 0 | 0 | 8.531524 | 8.713714 | 8.995549 | 0 | 0 | 0 |
| 556 | neg_556 | Hyperoside | 49.69215 | 53.98481 | 55.76099 | 149.0479 | 159.3666 | 168.7724 | 10.81359 | 11.77417 | 12.40008 | 154.2677 | 168.9332 | 170.4694 |
| 557 | neg_557 | Histidinyl-Glutamate | 25.57827 | 25.85496 | 26.55112 | 0 | 0 | 0 | 0 | 0 | 0 | 0 | 0 | 0 |
| 558 | neg_558 | Prolyl-Threonine | 1365.386 | 1513.778 | 1597.202 | 0 | 0 | 0 | 0 | 0 | 0 | 0 | 0 | 0 |
| 559 | neg_559 | Fexofenadine | 0 | 0 | 0 | 0 | 0 | 0 | 80.3293 | 85.96625 | 92.80588 | 0 | 0 | 0 |
| 560 | neg_560 | Histidinyl-Aspartate | 153.1465 | 165.258 | 171.3729 | 2.344394 | 2.455988 | 2.699331 | 6.395377 | 7.004649 | 7.218044 | 1.157111 | 1.248526 | 1.322403 |
| 561 | neg_561 | Shikimate | 100.1629 | 100.5693 | 106.4531 | 5.69789 | 6.243262 | 6.490482 | 7.663343 | 7.837596 | 8.478559 | 7.443471 | 7.778942 | 7.904384 |
| 562 | neg_562 | Cytidine 5'-diphosphocholine (CDP-choline) | 8.391707 | 9.298749 | 10.07057 | 37.09713 | 37.30981 | 37.74121 | 18.38948 | 19.49008 | 20.14255 | 29.43999 | 30.24376 | 32.6218 |
| 563 | neg_563 | Flavin mononucleotide (FMN) | 5105.742 | 5619.147 | 5841.381 | 11.93679 | 12.44905 | 12.66551 | 16.18529 | 16.78738 | 17.15212 | 7.765423 | 8.029045 | 8.613486 |
| 564 | neg_564 | Kojic Acid | 74881.57 | 75099.89 | 76542.11 | 81.36379 | 87.33416 | 93.84082 | 98.00087 | 101.1097 | 105.8956 | 90.51609 | 94.93503 | 101.4404 |
| 565 | neg_565 | Hieracin | 364.5313 | 378.29 | 400.1034 | 55.10299 | 58.82897 | 59.31816 | 50.83427 | 52.77554 | 57.5045 | 43.0295 | 45.47534 | 45.5469 |

Continued Table S2

| 566 | neg_566 | (+-)-Mevalonolactone | 42.01251 | 42.69864 | 43.90221 | 49.26843 | 52.00191 | 54.89731 | 37.31659 | 37.87547 | 37.96572 | 40.68828 | 44.55609 | 45.68167 |
| --- | --- | --- | --- | --- | --- | --- | --- | --- | --- | --- | --- | --- | --- | --- |
| 567 | neg_567 | n-Propyl cinnamate | 120.8367 | 130.9141 | 134.7152 | 196.0683 | 212.869 | 214.8988 | 249.4104 | 259.3402 | 284.6326 | 198.6244 | 203.1642 | 223.4328 |
| 568 | neg_568 | Prostaglandin B1 | 5.037356 | 5.154488 | 5.28866 | 2.030422 | 2.070882 | 2.203502 | 1.758023 | 1.90612 | 1.961461 | 5.354812 | 5.43778 | 5.762404 |
| 569 | neg_569 | Alanyl-Serine | 433.6171 | 438.2097 | 464.421 | 15.58497 | 17.3035 | 17.35202 | 297.7336 | 302.5439 | 328.8927 | 4.022127 | 4.0561 | 4.26804 |
| 570 | neg_570 | 4,6-Dioxoheptanoic acid | 231.3737 | 249.4548 | 273.9404 | 0 | 0 | 0 | 0 | 0 | 0 | 0 | 0 | 0 |
| 571 | neg_571 | 1-Methyluric acid | 77.51121 | 81.32011 | 84.61635 | 1.69799 | 1.698746 | 1.824379 | 2.340261 | 2.525944 | 2.691186 | 2.192862 | 2.324639 | 2.405721 |
| 572 | neg_572 | 4'-Hydroxyflurbiprofen | 804.1876 | 816.1205 | 830.7547 | 34.93732 | 37.6797 | 40.81155 | 61.1672 | 62.26459 | 67.7122 | 8.210436 | 8.711097 | 8.973286 |
| 573 | neg_573 | D-Glucono-1,5-lactone | 0.064956 | 0.069073 | 0.07236 | 457.6898 | 502.5674 | 515.1334 | 1988.646 | 2139.003 | 2158.437 | 2801.807 | 3088.069 | 3328.176 |
| 574 | neg_574 | Estrone | 54.94919 | 58.11671 | 62.53418 | 65.20745 | 71.96591 | 78.27724 | 62.05417 | 63.61923 | 66.2139 | 66.56126 | 71.91038 | 76.17725 |
| 575 | neg_575 | (-)-Naringenin | 44.71324 | 45.64593 | 50.06017 | 9.843402 | 10.10239 | 10.40665 | 7.343742 | 7.957212 | 8.132791 | 8.990056 | 9.438873 | 9.644387 |
| 576 | neg_576 | Suprofen | 314.0848 | 337.5654 | 338.2679 | 152.1991 | 166.9995 | 172.1614 | 818.7449 | 846.0517 | 878.5704 | 102.278 | 108.049 | 116.2531 |
| 577 | neg_577 | Silybin | 17.02502 | 18.62525 | 20.41015 | 40.51736 | 42.26113 | 45.24858 | 44.56036 | 46.68566 | 50.35465 | 69.2183 | 73.20638 | 75.42147 |
| 578 | neg_578 | Nafcillin | 65.82434 | 69.95463 | 71.36655 | 0.879384 | 0.897832 | 0.95836 | 1.620987 | 1.754217 | 1.928654 | 3.439114 | 3.538068 | 3.561447 |
| 579 | neg_579 | 5-methoxyuridine | 11.33839 | 11.8551 | 12.82681 | 39.01207 | 43.25025 | 47.33677 | 28.44101 | 29.49786 | 31.82606 | 49.40336 | 54.25829 | 58.75632 |
| 580 | neg_580 | Amarogentin | 637.0152 | 657.1737 | 657.6284 | 306.4273 | 339.9097 | 358.0059 | 395.5519 | 418.1336 | 438.8358 | 307.1421 | 320.7548 | 352.3329 |
| 581 | neg_581 | Diosmin | 280.6819 | 287.7403 | 299.4327 | 1413.774 | 1414.727 | 1473.357 | 1395.093 | 1487.487 | 1592.127 | 1340.586 | 1341.981 | 1361.465 |
| 582 | neg_582 | Hesperetin | 43.98952 | 45.51907 | 47.09423 | 2.200983 | 2.212196 | 2.420976 | 27.71282 | 30.5133 | 33.31744 | 0 | 0 | 0 |
| 583 | neg_583 | beta-Nicotinamide D-ribonucleotide | 73.86067 | 75.51109 | 82.87756 | 0 | 0 | 0 | 0 | 0 | 0 | 0 | 0 | 0 |
| 584 | neg_584 | Nicotinuric acid | 273.1494 | 295.0306 | 317.7102 | 0 | 0 | 0 | 10.12526 | 10.4654 | 10.76424 | 0 | 0 | 0 |
| 585 | neg_585 | .gamma.-L-Glu-.epsilon.-L-Lys | 2000.203 | 2166.774 | 2362.836 | 2165.345 | 2281.8 | 2487.026 | 2023.193 | 2206.899 | 2387.066 | 2378.644 | 2401.349 | 2543.748 |
| 586 | neg_586 | Boldine | 0 | 0 | 0 | 0 | 0 | 0 | 2.714546 | 2.835675 | 2.839664 | 9.317832 | 9.782733 | 10.12098 |
| 587 | neg_587 | Homatropine | 251.3231 | 252.1095 | 261.631 | 306.698 | 310.2213 | 333.4526 | 260.4427 | 284.977 | 299.0536 | 274.8766 | 304.1758 | 305.6754 |
| 588 | neg_588 | 4-Methylumbelliferyl beta-D-glucuronide | 85.66728 | 85.7466 | 88.23766 | 41.12672 | 45.21149 | 47.65105 | 50.46251 | 55.00843 | 56.36175 | 50.24293 | 51.36639 | 54.81184 |
| 589 | neg_589 | Prolyl-Histidine | 110.6253 | 121.0089 | 126.3605 | 4.784185 | 5.250437 | 5.454645 | 11.69785 | 12.42508 | 12.9352 | 4.021374 | 4.178624 | 4.313931 |
| 590 | neg_590 | theanine | 157.6794 | 161.414 | 166.1378 | 236.3326 | 240.6397 | 260.5496 | 403.8793 | 442.535 | 452.5041 | 254.2036 | 279.6522 | 304.7117 |
| 591 | neg_591 | Glycyl-Methionine | 1191.376 | 1255.765 | 1346.856 | 60.77966 | 63.13591 | 65.12897 | 37.69054 | 40.89692 | 42.40937 | 43.2422 | 43.87762 | 46.8137 |

Continued Table S2

| 592 | neg_592 | Sebacic acid | 300.1917 | 317.492 | 334.8191 | 14.74266 | 15.74384 | 16.3422 | 45.58475 | 45.78353 | 46.81072 | 16.47023 | 17.41165 | 18.46584 |
| --- | --- | --- | --- | --- | --- | --- | --- | --- | --- | --- | --- | --- | --- | --- |
| 593 | neg_593 | Cysteinyl-Asparagine | 0 | 0 | 0 | 3.685258 | 3.782919 | 4.103335 | 4.27762 | 4.617675 | 4.932338 | 10.96911 | 11.88875 | 12.35225 |
| 594 | neg_594 | Pyridoxamine 5'-phosphate | 50.55271 | 55.32687 | 60.14818 | 185.6042 | 188.5385 | 199.5967 | 213.7202 | 220.7852 | 227.2814 | 131.9404 | 138.6947 | 145.7681 |
| 595 | neg_595 | Histidinyl-Serine | 126.1408 | 128.1302 | 132.8837 | 17.50054 | 17.67068 | 18.56051 | 7.00525 | 7.295474 | 7.36506 | 14.05552 | 14.51689 | 15.61131 |
| 596 | neg_596 | Tryptophyl-Glutamate | 64.27736 | 64.51175 | 69.73841 | 57.04079 | 62.89339 | 63.26546 | 63.7708 | 66.60094 | 71.30724 | 55.86939 | 57.764 | 59.6735 |
| 597 | neg_597 | Esculetin | 14.32475 | 14.52386 | 15.2968 | 15.14192 | 16.46748 | 17.83464 | 125.6072 | 135.9248 | 142.0105 | 0 | 0 | 0 |
| 598 | neg_598 | Genkwanin | 17.33303 | 19.21761 | 20.47838 | 15.18825 | 15.19564 | 15.32262 | 12.83102 | 14.25265 | 14.48727 | 15.19197 | 15.50348 | 15.64164 |
| 599 | neg_599 | Glucotropaeolin | 223.0412 | 223.9757 | 226.5534 | 0.11985 | 0.120009 | 0.131679 | 0 | 0 | 0 | 0.0353 | 0.036443 | 0.038496 |
| 600 | neg_600 | Trimethoprim | 64.38429 | 64.96967 | 67.15894 | 46.12599 | 48.85452 | 52.41486 | 42.71708 | 45.06015 | 47.35135 | 48.82831 | 51.27967 | 55.68273 |
| 601 | neg_601 | ADP-ribose | 411.1061 | 421.4397 | 435.5271 | 223.7168 | 225.6051 | 240.1327 | 185.3536 | 204.7123 | 215.7838 | 161.027 | 178.9142 | 190.4481 |
| 602 | neg_602 | Dehydroascorbic acid (Oxidized vitamin C) | 163.6383 | 172.9633 | 183.2973 | 160.1112 | 167.5717 | 179.5293 | 155.0513 | 160.743 | 175.1078 | 150.8874 | 164.5024 | 166.8918 |
| 603 | neg_603 | 5-Methylcytosine | 231.2427 | 247.8613 | 262.4818 | 379.0757 | 383.6076 | 410.4932 | 320.696 | 350.2306 | 371.079 | 385.8191 | 399.5512 | 408.9804 |
| 604 | neg_604 | CITIOLONE | 81.54141 | 86.56326 | 87.40708 | 15.89474 | 17.33823 | 18.5122 | 21.29167 | 22.12807 | 22.92951 | 17.47642 | 19.33785 | 19.99291 |
| 605 | neg_605 | Glycyl-Valine | 29618.74 | 31999.92 | 32510.76 | 26526.58 | 28660.12 | 31382.51 | 26358.12 | 28821.72 | 30977.28 | 21911.08 | 22379.59 | 24115.49 |
| 606 | neg_606 | (R)-mevalonic acid 5-Phosphate | 721.356 | 751.232 | 758.7485 | 17.83744 | 18.26723 | 19.2844 | 17.93772 | 18.02894 | 18.28661 | 10.67862 | 11.12905 | 11.77947 |
| 607 | neg_607 | Mitoxantrone | 0 | 0 | 0 | 0 | 0 | 0 | 978.221 | 989.0936 | 1008.363 | 0 | 0 | 0 |
| 608 | neg_608 | Geldanamycin | 127.3193 | 133.012 | 133.0492 | 47.67003 | 51.63529 | 53.93065 | 15.96627 | 16.78395 | 16.78851 | 50.23453 | 50.70525 | 50.74061 |
| 609 | neg_609 | Metsulfuron-methyl | 7.808979 | 8.214794 | 8.547147 | 33.75251 | 34.99227 | 38.05609 | 2.989738 | 3.287817 | 3.510399 | 20.3665 | 20.76482 | 21.89969 |
| 610 | neg_610 | 3,4-Dihydroxymandelic acid | 87.48725 | 92.54844 | 93.35956 | 0.657389 | 0.721591 | 0.777852 | 5.241036 | 5.566521 | 6.065839 | 0 | 0 | 0 |
| 611 | neg_611 | trans-cinnamate | 637.3949 | 644.7288 | 657.9239 | 0 | 0 | 0 | 1.000564 | 1.066095 | 1.120397 | 0 | 0 | 0 |
| 612 | neg_612 | Sulfamethizole | 45.24885 | 49.27873 | 51.25649 | 1199.278 | 1252.549 | 1261.307 | 1452.132 | 1487.272 | 1565.421 | 791.1211 | 817.625 | 826.558 |
| 613 | neg_613 | Glycyl-Histidine | 119.802 | 122.1724 | 133.335 | 27.65286 | 29.12045 | 30.49161 | 88.48178 | 90.30199 | 96.03666 | 4.825121 | 5.173961 | 5.297606 |
| 614 | neg_614 | Carbamazepine | 260.5364 | 278.9802 | 299.653 | 0 | 0 | 0 | 0 | 0 | 0 | 0 | 0 | 0 |
| 615 | neg_615 | (-)-Epicatechin | 598.4368 | 626.4696 | 639.3192 | 19.40868 | 20.90972 | 21.51624 | 247.7411 | 259.528 | 270.0925 | 2.064554 | 2.188919 | 2.400053 |
| 616 | pos_1 | Theobromine | 21837.25 | 22341.07 | 23988.14 | 21185.63 | 22390.11 | 23683.77 | 20418.07 | 21609.58 | 21950.25 | 22414.58 | 23422.81 | 24997.05 |
| 617 | pos_2 | Caffeine | 297351.9 | 322753.9 | 329344.9 | 328098.7 | 330234.4 | 336091.1 | 315992.8 | 321427.8 | 341430.2 | 306671.6 | 332466 | 353307 |

Continued Table S2

| 618 | pos_3 | 4-Methylcatechol | 112.7092 | 118.4932 | 121.1117 | 25.14279 | 26.07873 | 28.03649 | 30.63418 | 31.04497 | 33.39308 | 19.15566 | 21.10311 | 22.46706 |
| --- | --- | --- | --- | --- | --- | --- | --- | --- | --- | --- | --- | --- | --- | --- |
| 619 | pos_4 | epsilon-Captrolactone | 110936.1 | 121064.2 | 123581.1 | 116015.5 | 121896.9 | 130826 | 114117.1 | 117417.5 | 126197.1 | 121614.9 | 123671.5 | 126121.5 |
| 620 | pos_5 | D-Pyroglutamic acid | 4724.309 | 5099.603 | 5178.275 | 3545.462 | 3693.243 | 3890.035 | 610.4581 | 669.0365 | 708.5632 | 4868.207 | 5044.774 | 5342.799 |
| 621 | pos_6 | Citric acid | 242.6515 | 262.4099 | 264.2966 | 257.6064 | 266.0603 | 281.2188 | 249.8982 | 257.1304 | 257.2742 | 236.3603 | 255.283 | 278.4352 |
| 622 | pos_7 | Decanoic acid | 211.3012 | 217.0014 | 235.4312 | 273.7948 | 304.1983 | 327.4381 | 224.1607 | 227.7174 | 247.4724 | 291.0042 | 291.4794 | 306.9351 |
| 623 | pos_8 | p-Coumaric acid | 3097.468 | 3352.771 | 3371.54 | 66.1174 | 70.3027 | 75.01013 | 106.6705 | 108.6089 | 118.9392 | 92.78261 | 93.98306 | 102.2092 |
| 624 | pos_9 | Phospho(enol)pyruvic acid monopotassium salt | 27.44133 | 30.02235 | 31.30963 | 30.7872 | 32.3369 | 34.61616 | 24.31219 | 24.93501 | 25.45479 | 29.06714 | 29.21341 | 30.60935 |
| 625 | pos_10 | Squalene | 119.0705 | 120.2185 | 120.3157 | 114.4939 | 126.5484 | 138.8923 | 127.4091 | 129.0061 | 130.0371 | 103.7339 | 107.6854 | 111.2823 |
| 626 | pos_11 | Dioctyl Phthalate | 21756.72 | 23910.29 | 25170.2 | 20857.42 | 21597.37 | 22649.58 | 27888.12 | 29336.05 | 30481.26 | 19484.93 | 21119.61 | 22486.95 |
| 627 | pos_12 | Trisodium 2-metylcitrate, racemic mixture | 11602.67 | 12438.57 | 13098.18 | 72.70412 | 74.17336 | 77.37144 | 111.7931 | 117.0628 | 124.8122 | 61.42044 | 68.19578 | 74.57199 |
| 628 | pos_13 | L-Lysine monohydrochloride | 430.1384 | 434.7546 | 467.9238 | 1.075599 | 1.136445 | 1.245444 | 79.48352 | 81.18882 | 87.84441 | 0 | 0 | 0 |
| 629 | pos_14 | Indole-3-acetic acid sodium salt | 0.081479 | 0.088286 | 0.090212 | 38.87639 | 41.0183 | 41.06736 | 18.9835 | 19.48108 | 20.39001 | 41.61409 | 42.44626 | 43.06625 |
| 630 | pos_15 | Xanthurenic acid | 151.2687 | 158.6896 | 161.1831 | 231.4297 | 245.8624 | 259.8864 | 162.9789 | 172.5655 | 183.9948 | 224.0239 | 226.105 | 229.9881 |
| 631 | pos_16 | Guanosine | 235.5325 | 253.3853 | 260.5772 | 29.37712 | 30.06149 | 32.43635 | 27.4619 | 29.33956 | 31.7569 | 103.1099 | 108.6874 | 116.0185 |
| 632 | pos_17 | Guanosine 5'-monophosphate disodium salt hydrate | 1254.591 | 1258.221 | 1283.167 | 1070.765 | 1119.675 | 1192.031 | 801.1291 | 875.0817 | 948.6202 | 1184.504 | 1196.265 | 1249.324 |
| 633 | pos_18 | Diethyl oxalpropionate | 90.58936 | 95.7784 | 99.13876 | 83.03362 | 87.35484 | 88.10473 | 82.07308 | 86.38073 | 90.76932 | 89.99183 | 95.2182 | 103.068 |
| 634 | pos_19 | S-Methyl-5'-thioadenosine | 406.8919 | 449.266 | 450.6751 | 47.54871 | 48.14134 | 48.35687 | 18.64539 | 20.37371 | 20.94675 | 46.28702 | 49.92882 | 54.12992 |
| 635 | pos_20 | mono-Ethyl malonate | 251.2667 | 257.7262 | 273.3698 | 258.6078 | 261.2721 | 278.4392 | 224.4111 | 244.6564 | 266.7008 | 231.8012 | 255.7126 | 279.4054 |
| 636 | pos_21 | Riboflavin | 52.32144 | 57.36288 | 58.33836 | 125.8585 | 133.4411 | 145.9391 | 94.13665 | 94.1496 | 96.80538 | 128.2806 | 141.9812 | 155.9323 |
| 637 | pos_22 | D-Ribose 5-phosphate disodium salt hydrate | 103.7386 | 104.7466 | 110.7047 | 414.4536 | 452.6676 | 484.4399 | 160.9642 | 163.7581 | 168.8264 | 447.0683 | 483.373 | 507.834 |
| 638 | pos_23 | Kynurenic acid | 108.7407 | 113.9131 | 119.723 | 2.143711 | 2.30385 | 2.397694 | 1.080426 | 1.136419 | 1.224923 | 0.09202 | 0.099233 | 0.108918 |
| 639 | pos_24 | Curvularide C | 745.9256 | 763.4119 | 831.2799 | 711.7105 | 736.8646 | 740.0924 | 857.9385 | 906.6384 | 950.9421 | 616.7094 | 672.0864 | 695.1133 |
| 640 | pos_25 | borrelidin | 111.1081 | 122.91 | 125.499 | 126.2368 | 130.8333 | 142.4453 | 110.5347 | 116.7933 | 116.8848 | 107.5576 | 117.5952 | 122.4782 |
| 641 | pos_26 | Sphinganine | 19007.21 | 20408.21 | 21898.28 | 20355.36 | 21220.49 | 21778.04 | 15770.15 | 16110.29 | 16378.13 | 15523.88 | 17137.62 | 17437.46 |

Continued Table S2

| 642 | pos_27 | Kaempferol 3-O-glucoside | 5.017566 | 5.03709 | 5.522684 | 533.4694 | 543.293 | 544.2756 | 378.1877 | 405.3297 | 409.5561 | 566.9239 | 575.9767 | 619.9873 |
| --- | --- | --- | --- | --- | --- | --- | --- | --- | --- | --- | --- | --- | --- | --- |
| 643 | pos_28 | methyl-1,4-benzoquinol | 181.9209 | 200.6911 | 201.4618 | 48.41844 | 49.54862 | 50.09503 | 46.03452 | 47.64627 | 50.87644 | 44.79536 | 47.61603 | 51.34905 |
| 644 | pos_29 | (2S,3S,4R)-2-aminohexadecane-1,3,4-triol | 4303.869 | 4344.622 | 4687.226 | 5289.583 | 5574.018 | 6057.404 | 4432.098 | 4483.105 | 4571.766 | 4513.406 | 4639.993 | 4718.291 |
| 645 | pos_30 | Undecanoic acid | 163.4157 | 173.2541 | 185.3368 | 236.9977 | 247.6492 | 253.2245 | 189.3495 | 190.832 | 205.7836 | 238.6136 | 249.5194 | 268.0653 |
| 646 | pos_31 | capric acid | 16945.34 | 18386.46 | 20140.56 | 17597.2 | 19014.1 | 20476.46 | 15047.12 | 15549.66 | 16277.31 | 19827.01 | 20669.15 | 21270.58 |
| 647 | pos_32 | lauric acid | 87.8913 | 94.52401 | 102.7579 | 153.7892 | 165.3592 | 168.2329 | 81.15796 | 88.30017 | 90.37542 | 154.2879 | 165.7012 | 168.0083 |
| 648 | pos_33 | 3,4-Dihydroxymandelaldehyde | 1939.068 | 2151.502 | 2151.535 | 51.06259 | 56.65768 | 56.77286 | 66.80386 | 71.76424 | 72.50996 | 54.36191 | 55.62093 | 60.09151 |
| 649 | pos_34 | L-Lysine | 887.8154 | 920.0266 | 999.5561 | 999.3217 | 1044.052 | 1130.734 | 1023.281 | 1026.319 | 1067.929 | 1088.948 | 1091.758 | 1124.575 |
| 650 | pos_35 | Astilbin | 352.3472 | 388.9393 | 406.5916 | 254.0942 | 262.4027 | 277.01 | 305.752 | 306.6067 | 308.9797 | 308.5659 | 320.1473 | 346.4443 |
| 651 | pos_36 | trans-Cinnamic acid | 15133.56 | 16318.93 | 16797.73 | 1228.788 | 1293.723 | 1381.674 | 2108.983 | 2273.025 | 2399.595 | 403.1485 | 421.8451 | 462.652 |
| 652 | pos_37 | Norleucine | 57.68957 | 63.91452 | 66.63069 | 12.06192 | 12.50905 | 12.52461 | 11.42459 | 11.6622 | 12.40603 | 11.28114 | 12.366 | 13.41707 |
| 653 | pos_38 | Shikimic acid | 10115.44 | 11194.56 | 11222.41 | 207.802 | 230.756 | 236.9153 | 436.423 | 475.0871 | 484.8181 | 181.6776 | 192.9313 | 200.0079 |
| 654 | pos_39 | Catechin | 30929.5 | 31238.68 | 33461.2 | 259.8173 | 268.7313 | 292.4925 | 1699.046 | 1736.927 | 1889.078 | 228.1634 | 247.7047 | 251.0219 |
| 655 | pos_40 | PS(18:0/0:0) | 1231.361 | 1240.646 | 1249.579 | 1137.563 | 1185.892 | 1204.825 | 1128.985 | 1230.302 | 1265.737 | 1050.258 | 1095.075 | 1133.169 |
| 656 | pos_41 | Phenylpyruvic acid | 12704.93 | 13539.71 | 13805.19 | 72.92233 | 75.0446 | 78.03281 | 280.3105 | 300.4999 | 317.7719 | 117.3327 | 124.8656 | 135.6164 |
| 657 | pos_42 | Epigallocatechin | 16190.84 | 17559.66 | 17915.33 | 76.5966 | 78.32796 | 84.97555 | 153.2765 | 164.5185 | 168.0485 | 74.93052 | 79.58747 | 81.92714 |
| 658 | pos_43 | Isorhamnetin 3-O-glucoside | 0 | 0 | 0 | 59.72451 | 66.31977 | 71.48737 | 36.48013 | 37.75086 | 40.11538 | 46.86527 | 49.68415 | 51.69781 |
| 659 | pos_44 | 1-Nitro-7-glutathionyl-8-hydroxy-7,8-dihydronaphthalene | 23.69514 | 24.11897 | 25.5453 | 47.72239 | 47.93996 | 48.4365 | 63.39428 | 64.14638 | 70.49144 | 39.43166 | 42.5244 | 44.09267 |
| 660 | pos_45 | 4-Methylacetophenone | 258.6458 | 276.2831 | 300.9295 | 328.0126 | 344.7308 | 364.3125 | 252.5812 | 277.8725 | 280.6451 | 321.6066 | 332.6456 | 343.7529 |
| 661 | pos_46 | Protoanemonin | 22.5918 | 23.89158 | 25.27565 | 26.42322 | 28.83693 | 28.8981 | 21.88182 | 23.09481 | 23.7436 | 24.3105 | 24.99975 | 25.86801 |
| 662 | pos_47 | 5'-n-propylthioadenosine | 9.671283 | 9.840849 | 10.42676 | 82.7135 | 86.98229 | 88.86223 | 19.79655 | 21.59014 | 21.78182 | 67.80091 | 70.38944 | 70.68949 |
| 663 | pos_48 | PE(18:1(11Z)/19:0) | 893713.6 | 960284.8 | 970921.3 | 739744 | 818731.1 | 829611.1 | 831072.5 | 905847.3 | 971535 | 658585.6 | 710241.5 | 723477 |
| 664 | pos_49 | 6-Phosphonoglucono-D-lactone | 26.50363 | 27.63594 | 28.49787 | 27.3268 | 28.60552 | 31.2229 | 22.3932 | 22.43505 | 23.8804 | 91.3875 | 96.62719 | 99.96426 |
| 665 | pos_50 | 7-Methylguanosine 5'-phosphate | 159.8103 | 171.598 | 178.0356 | 0.791458 | 0.815678 | 0.848918 | 14.24237 | 15.28372 | 16.80155 | 2.858892 | 3.024665 | 3.158323 |
| 666 | pos_51 | diacetylchitobiose-6-phosphate | 189.7103 | 192.678 | 207.827 | 189.42 | 195.8839 | 210.043 | 220.3182 | 223.3434 | 244.9876 | 176.0401 | 177.3113 | 186.2453 |

Continued Table S2

| 667 | pos_52 | Leucocyanidin | 15431.46 | 15461.91 | 15836.01 | 86.98859 | 88.34604 | 95.21972 | 112.8721 | 120.965 | 122.641 | 73.23694 | 78.59126 | 86.18865 |
| --- | --- | --- | --- | --- | --- | --- | --- | --- | --- | --- | --- | --- | --- | --- |
| 668 | pos_53 | tryptophol | 6273.786 | 6569.82 | 6845.229 | 6914.47 | 6953.622 | 7057.498 | 6293.91 | 6735.303 | 7267.553 | 6844.176 | 7081.855 | 7787.634 |
| 669 | pos_54 | L-Arogenate | 122.297 | 134.2656 | 145.0337 | 131.664 | 131.9358 | 143.61 | 123.3348 | 133.9012 | 145.9834 | 126.1474 | 139.1007 | 142.3172 |
| 670 | pos_55 | 4-oxo-N-acetylneuraminate | 74531.29 | 80726.91 | 81049.94 | 1146.937 | 1203.231 | 1209.933 | 1339.469 | 1404.547 | 1498.096 | 717.0801 | 738.8861 | 755.4321 |
| 671 | pos_56 | Delphinidin 3-O-glucoside | 365.2496 | 390.137 | 404.4507 | 1321.06 | 1429.213 | 1538.719 | 345.6957 | 368.7736 | 385.9517 | 1148.872 | 1251.38 | 1315.077 |
| 672 | pos_57 | Uridine diphosphate-N-acetylgalactosamine | 308.2854 | 327.9521 | 352.8148 | 1455.847 | 1555.182 | 1605.744 | 1291.103 | 1373.955 | 1474.208 | 1847.578 | 1901.567 | 1953.567 |
| 673 | pos_58 | Hydroxyproline | 0 | 0 | 0 | 1585.651 | 1608.519 | 1690.085 | 359.8703 | 362.6819 | 366.7804 | 1194.878 | 1310.865 | 1370.093 |
| 674 | pos_59 | 5'-azido-5-deoxyuridine | 34.56868 | 37.24357 | 38.12627 | 157.87 | 174.0091 | 177.329 | 98.59074 | 100.7398 | 107.8543 | 225.8388 | 227.0361 | 237.4093 |
| 675 | pos_60 | PS(14:0/18:1(11Z)) | 6749.446 | 7203.242 | 7794.909 | 5245.419 | 5756.242 | 6052.731 | 7035.195 | 7468.489 | 7940.814 | 9436.834 | 10044.15 | 11030.89 |
| 676 | pos_61 | PS(18:1(11Z)/16:0) | 4016.402 | 4120.765 | 4375.817 | 3093.647 | 3376.757 | 3631.724 | 4002.03 | 4234.786 | 4570.289 | 5140.827 | 5678.575 | 6228.94 |
| 677 | pos_62 | Vitisin A | 15358.3 | 15970.09 | 16585 | 1497.116 | 1655.47 | 1804.181 | 3500.572 | 3787.662 | 4135.674 | 4323.515 | 4522.369 | 4779.114 |
| 678 | pos_63 | Peonidin 3-O-glucoside | 20.00653 | 21.97034 | 22.42064 | 11.06918 | 11.31178 | 11.54228 | 24.36466 | 25.04154 | 25.89407 | 4.847132 | 4.852512 | 5.048254 |
| 679 | pos_64 | benzyl alcohol | 367.2161 | 379.5238 | 382.0524 | 176.3372 | 193.2017 | 211.5448 | 173.9941 | 185.6266 | 192.5736 | 161.184 | 166.8871 | 171.1172 |
| 680 | pos_65 | 5-Amino-6-(5'-phosphoribitylamino)uracil | 529.8418 | 554.4864 | 600.6783 | 618.3606 | 670.3902 | 672.6651 | 1464.595 | 1550.731 | 1557.748 | 508.1851 | 532.6027 | 556.3644 |
| 681 | pos_66 | UDP-N-acetyl-D-mannosamine | 25.87894 | 27.35394 | 27.71264 | 639.7206 | 654.9933 | 683.2589 | 528.2549 | 578.8742 | 619.4287 | 570.0016 | 579.186 | 591.1607 |
| 682 | pos_67 | Malvidin 3-O-(cafeoylglucoside) | 0 | 0 | 0 | 129.8598 | 129.9217 | 140.9206 | 110.9407 | 115.6847 | 117.1251 | 120.8782 | 130.9913 | 139.3654 |
| 683 | pos_68 | Gamma-glutamyl-L-putrescine | 2822.44 | 2868.593 | 3129.664 | 2949.805 | 3023.364 | 3023.573 | 2675.026 | 2884.27 | 3063.099 | 2715.9 | 2793.876 | 2818.192 |
| 684 | pos_69 | Heptadecanoic acid | 1103.373 | 1108.971 | 1115.543 | 1024.861 | 1074.644 | 1172.708 | 982.7716 | 1054.161 | 1097.404 | 1003.001 | 1004.336 | 1014.948 |
| 685 | pos_70 | 2,5-Diamino-6-(5'-phosphoribosylamino)-4-pyrimidineone | 1304.21 | 1401.397 | 1448.093 | 195.5506 | 208.1114 | 209.2969 | 182.508 | 197.95 | 208.8764 | 181.6753 | 190.6501 | 202.9527 |
| 686 | pos_71 | dCMP | 107.8333 | 111.6879 | 117.3753 | 12.1123 | 12.48109 | 13.60142 | 73.23384 | 75.50571 | 78.67464 | 6.981857 | 7.20099 | 7.915116 |
| 687 | pos_72 | Guanosine diphosphate mannose | 85.12165 | 87.49266 | 90.07816 | 126.176 | 139.5331 | 148.5672 | 166.1453 | 167.8158 | 172.5944 | 133.1691 | 138.1419 | 144.706 |
| 688 | pos_73 | Limonene | 574.8648 | 632.7009 | 650.968 | 667.0386 | 709.1688 | 734.3065 | 603.4961 | 637.8838 | 674.6574 | 598.6475 | 610.3843 | 648.3979 |
| 689 | pos_74 | 6-diazo-5-oxo-L-norleucine | 80.04126 | 84.33374 | 87.17119 | 77.61399 | 77.98746 | 84.88982 | 71.81654 | 79.33425 | 79.92562 | 73.59196 | 78.62224 | 79.1519 |
| 690 | pos_75 | Quercetin 3-O-glucoside | 221.3017 | 236.481 | 255.2643 | 702.9762 | 730.3612 | 739.8583 | 267.3748 | 281.1248 | 296.5119 | 655.0513 | 660.8911 | 668.3528 |
| 691 | pos_76 | Quercetin | 1332.783 | 1388.782 | 1391.173 | 0 | 0 | 0 | 52.97761 | 54.6784 | 55.7004 | 2.721418 | 2.935703 | 3.14306 |

Continued Table S2

| 692 | pos_77 | Kaempferol 3-O-glucuronide | 15.34235 | 15.55442 | 16.64879 | 234.011 | 255.0753 | 260.5789 | 64.45399 | 68.41821 | 72.76973 | 258.5839 | 279.4755 | 303.2632 |
| --- | --- | --- | --- | --- | --- | --- | --- | --- | --- | --- | --- | --- | --- | --- |
| 693 | pos_78 | PC(16:0/18:2(9Z,12Z)) | 450317.4 | 464548.1 | 474967 | 387282.3 | 388988.3 | 390677.6 | 399987.7 | 412554.9 | 418613.8 | 298191 | 309481.7 | 312703.2 |
| 694 | pos_79 | beta-N-acetylneuraminate | 52625.95 | 54932.84 | 55390.13 | 1413.424 | 1556.055 | 1615.88 | 34159.34 | 35002.33 | 37731.71 | 249.9677 | 258.0606 | 266.6024 |
| 695 | pos_80 | Adenosine 2'-phosphate | 185.287 | 187.3031 | 198.3296 | 120.6055 | 123.0322 | 129.1164 | 101.7788 | 103.3373 | 109.3187 | 128.1645 | 135.1162 | 145.6005 |
| 696 | pos_81 | N-Acetylmuramoyl-Ala | 230.8485 | 248.6574 | 266.7799 | 1097.637 | 1174.551 | 1199.636 | 230.2548 | 234.0347 | 255.1549 | 279.8302 | 280.5513 | 284.0879 |
| 697 | pos_82 | 1-(sn-glycero-3-phospho)-1D-myo-inositol | 291.894 | 304.3416 | 311.97 | 11.68089 | 12.07081 | 12.66646 | 15.63852 | 15.9937 | 17.04457 | 11.7196 | 12.36835 | 13.04338 |
| 698 | pos_83 | L-Carnitine | 149.1165 | 152.2488 | 154.0543 | 181.3845 | 187.8843 | 206.2031 | 168.7997 | 180.5169 | 198.1554 | 203.5899 | 211.119 | 212.9098 |
| 699 | pos_84 | 5-methyl-2-furancarboxaldehyde | 0 | 0 | 0 | 40.31526 | 41.42911 | 42.32291 | 0.022352 | 0.024806 | 0.025494 | 47.37078 | 52.35339 | 55.61886 |
| 700 | pos_85 | Vanillic acid | 880.3746 | 941.0453 | 974.335 | 67.28443 | 69.71888 | 75.49283 | 41.27544 | 45.18993 | 45.7102 | 100.1539 | 104.8839 | 110.5498 |
| 701 | pos_86 | Thymidine | 174.1128 | 181.0555 | 181.651 | 161.2112 | 171.2723 | 184.486 | 1369.744 | 1396.064 | 1504.365 | 135.2281 | 144.2604 | 156.2181 |
| 702 | pos_87 | cerulenin | 107.4853 | 114.4933 | 117.6838 | 54.1157 | 54.87747 | 59.18792 | 76.28217 | 76.75081 | 80.77557 | 46.45962 | 49.4816 | 49.65375 |
| 703 | pos_88 | Cyanidin 3-O-(coumaroylglucoside) | 34.34183 | 34.43197 | 36.10753 | 17.55052 | 18.11315 | 18.12354 | 28.95022 | 30.64892 | 33.43709 | 20.73744 | 22.25069 | 23.57689 |
| 704 | pos_89 | CDP-Choline | 21.00122 | 21.30555 | 22.03614 | 28.7471 | 31.7576 | 33.60994 | 18.55408 | 19.66869 | 21.57491 | 21.54461 | 23.40716 | 23.75163 |
| 705 | pos_90 | Isochorismate | 19.89097 | 20.43007 | 21.92131 | 28.27546 | 30.97154 | 33.64659 | 28.99475 | 30.10656 | 30.22343 | 31.56484 | 34.79621 | 36.13724 |
| 706 | pos_91 | Engeletin | 493.8008 | 547.7101 | 577.6593 | 18.62153 | 20.10145 | 20.16085 | 29.78622 | 31.8586 | 34.04667 | 11.21212 | 11.62283 | 12.6555 |
| 707 | pos_92 | 2-Succinyl-5-enolpyruvyl-6-hydroxy-3-cyclohexene-1-carboxylate | 547.9439 | 606.1163 | 644.0566 | 7.356456 | 7.656766 | 7.730481 | 29.95579 | 32.52023 | 33.02986 | 4.265735 | 4.553367 | 4.964396 |
| 708 | pos_93 | D-2-aminopentanoic acid | 2830.043 | 3037.846 | 3054.872 | 2889.234 | 3014.134 | 3249.869 | 2590.221 | 2803.89 | 2827.057 | 2814.189 | 2999.354 | 3133.742 |
| 709 | pos_94 | nicotinic acid D-ribonucleotide | 314.11 | 331.5694 | 351.2432 | 386.554 | 427.5626 | 456.1959 | 360.6786 | 371.2926 | 373.7331 | 421.9858 | 425.6322 | 452.5973 |
| 710 | pos_95 | DDP-N-acetylmuramoyl-L-alanyl-D-glutamate | 497.4718 | 547.8497 | 554.0836 | 892.9791 | 937.9745 | 986.4048 | 766.4017 | 802.9002 | 815.4614 | 798.2817 | 886.1737 | 938.5644 |
| 711 | pos_96 | PG(16:1(9Z)/18:1(11Z)) | 40807.73 | 42302.26 | 46369.98 | 38763.85 | 39127.48 | 39879.22 | 36271.84 | 39318.29 | 40784.75 | 37983.71 | 37988.72 | 40097.87 |
| 712 | pos_97 | Deoxythymidine diphosphate-l-rhamnose | 354.6156 | 385.2197 | 420.3923 | 1.384268 | 1.416209 | 1.416791 | 9.018435 | 9.16009 | 9.234709 | 3.781954 | 4.006573 | 4.182465 |
| 713 | pos_98 | Adenosine diphosphate ribose | 790.8013 | 878.6349 | 895.1688 | 636.5408 | 671.0359 | 710.2183 | 758.573 | 770.6059 | 795.8289 | 580.2365 | 627.7533 | 643.9386 |
| 714 | pos_99 | Isopropylmaleate | 4110.358 | 4548.477 | 4842.677 | 131.5531 | 131.7822 | 133.1259 | 137.258 | 142.6278 | 146.6869 | 123.2604 | 132.2923 | 142.162 |
| 715 | pos_100 | 3-Deoxy-D-manno-octulosonate | 96.20252 | 100.0039 | 107.5001 | 42.01162 | 44.68414 | 46.1072 | 41.45346 | 44.96967 | 48.51021 | 46.42094 | 46.46483 | 51.10466 |

Continued Table S2

| 716 | pos_101 | gamma-glutamyl-ethylamide | 10698.08 | 10830.92 | 11171.2 | 12268.31 | 12366.43 | 12912.18 | 11676.56 | 12075.57 | 12766.19 | 12031.77 | 13249.48 | 14140.78 |
| --- | --- | --- | --- | --- | --- | --- | --- | --- | --- | --- | --- | --- | --- | --- |
| 717 | pos_102 | Carnosine | 56.15473 | 59.41172 | 61.86223 | 3.523774 | 3.555741 | 3.885574 | 4.861056 | 5.365115 | 5.706746 | 8.050945 | 8.630159 | 8.794507 |
| 718 | pos_103 | PE(18:1(11Z)/18:1(11Z)) | 11628.55 | 12331.18 | 13167.05 | 11318.46 | 11761.11 | 12810.95 | 10945.2 | 11796.24 | 11885.22 | 11297.4 | 11320.41 | 12013.94 |
| 719 | pos_104 | D-Fructuronate | 65.95327 | 68.38727 | 72.71776 | 99.908 | 107.2828 | 109.4397 | 113.0826 | 113.6979 | 114.5571 | 58.7137 | 59.98286 | 62.66717 |
| 720 | pos_105 | Citicoline | 61.09675 | 66.19302 | 68.27277 | 26.03761 | 27.0197 | 29.29727 | 36.17373 | 37.40323 | 40.91031 | 37.2036 | 39.96897 | 41.36284 |
| 721 | pos_106 | NMNH | 25.61547 | 25.76494 | 27.90067 | 54.27978 | 56.425 | 57.34428 | 72.32181 | 77.25779 | 81.19225 | 75.63934 | 81.95576 | 88.576 |
| 722 | pos_107 | L-d-1-Pyrroline-5-carboxylic acid | 96.15018 | 105.7836 | 115.4552 | 106.6389 | 114.5981 | 124.5755 | 101.2754 | 105.7627 | 112.1647 | 106.1632 | 114.1349 | 117.0361 |
| 723 | pos_108 | Epicatechin gallate | 53268.22 | 55919.68 | 57463.4 | 4440.629 | 4693.275 | 4825.067 | 3587.858 | 3656.75 | 3829.404 | 4145.845 | 4294.746 | 4652.884 |
| 724 | pos_109 | 3-Oxo-beta-ionone | 19.60446 | 19.67117 | 20.59816 | 15.16543 | 16.23263 | 17.02546 | 12.58848 | 13.67831 | 14.42814 | 6.090527 | 6.256714 | 6.770311 |
| 725 | pos_110 | (R)-pantolactone | 423.2267 | 429.5298 | 462.5327 | 17.79301 | 18.90277 | 20.54869 | 19.90165 | 21.92618 | 22.7461 | 15.87293 | 16.7864 | 17.25732 |
| 726 | pos_111 | cis-3-(Carboxy-ethyl)-3,5-cyclo-hexadiene-1,2-diol | 7676.728 | 8026.011 | 8250.239 | 1230.614 | 1263.08 | 1279.146 | 948.9188 | 966.3187 | 1012.447 | 718.2292 | 791.5243 | 801.9263 |
| 727 | pos_112 | Nicotinamide riboside | 233.1734 | 243.0476 | 261.2002 | 103.5312 | 105.2988 | 112.9126 | 137.7446 | 138.3801 | 147.4351 | 158.2277 | 175.8071 | 177.6789 |
| 728 | pos_113 | SAICAR | 179.7615 | 185.5874 | 198.188 | 8.103381 | 8.128245 | 8.366312 | 15.55165 | 15.99984 | 17.49009 | 6.912467 | 7.17492 | 7.236823 |
| 729 | pos_114 | 2-amino-6-(hydroxymethyl)-7,8-dihydropteridin-4-ol | 45.50524 | 50.16212 | 54.56447 | 115.3452 | 119.5726 | 120.9457 | 86.71084 | 94.72164 | 95.70343 | 115.1478 | 116.9667 | 122.1042 |
| 730 | pos_115 | 5-Amino-1-(5-phospho-D-ribosyl)imidazole-4-carboxamide | 403.7815 | 406.5875 | 443.448 | 684.3593 | 707.4423 | 737.1695 | 5902.94 | 6347.605 | 6800.575 | 513.1814 | 559.774 | 569.7174 |
| 731 | pos_116 | 7,8-dihydroneopterin 3'-phosphate | 46.74615 | 48.97611 | 51.67361 | 53.87791 | 56.88925 | 59.24248 | 20.10052 | 22.26862 | 23.53981 | 51.47982 | 52.51436 | 53.7973 |
| 732 | pos_117 | Decanal | 51096.78 | 52136.56 | 54058.23 | 55192.77 | 57810.1 | 62871.49 | 45818.21 | 47501.73 | 49207.72 | 72422.26 | 74052.21 | 80110.19 |
| 733 | pos_118 | 2'-Deoxyinosine | 332.4441 | 333.3961 | 360.1558 | 417.835 | 449.8789 | 462.0957 | 339.4352 | 363.8124 | 369.46 | 527.8364 | 546.566 | 576.1431 |
| 734 | pos_119 | GDP-L-fucose | 154.2029 | 170.1947 | 185.1271 | 284.8857 | 311.0478 | 322.2989 | 274.2765 | 303.0454 | 328.4066 | 425.2878 | 441.854 | 474.4645 |
| 735 | pos_120 | PS(18:1(9Z)/0:0) | 315.1282 | 327.8213 | 333.8443 | 362.6549 | 375.3287 | 388.6212 | 345.1139 | 352.8011 | 371.2404 | 377.5373 | 382.9014 | 388.562 |
| 736 | pos_121 | 1-phospho-N-acetylmuramoyl-L-alanyl-D-glutamate | 443.6151 | 472.5851 | 496.0715 | 636.989 | 666.1221 | 716.2649 | 773.2333 | 777.1164 | 815.9878 | 542.7809 | 579.033 | 599.2119 |
| 737 | pos_122 | Myricetin 3-O-glucoside | 4912.307 | 5086.855 | 5126.586 | 24.25441 | 26.76927 | 29.01232 | 108.2686 | 118.175 | 124.2491 | 38.49543 | 40.59111 | 44.35846 |

Continued Table S2

| 738 | pos_123 | 2,3-Dihydro-2,5-dihydoxy-6-methyl-4-H-pyran-4-one | 1487.647 | 1572.815 | 1646.655 | 227.1645 | 249.4693 | 261.0141 | 203.0079 | 205.7017 | 212.5089 | 201.6625 | 220.5675 | 239.1241 |
| --- | --- | --- | --- | --- | --- | --- | --- | --- | --- | --- | --- | --- | --- | --- |
| 739 | pos_124 | E-Linalool oxide | 113.4234 | 117.7151 | 127.0386 | 109.8903 | 117.141 | 121.0644 | 117.6776 | 123.2659 | 127.0484 | 107.6505 | 116.0185 | 116.9995 |
| 740 | pos_125 | Cytidine | 26.28824 | 28.22466 | 28.75535 | 53.07712 | 58.60027 | 63.41331 | 57.1294 | 59.20179 | 64.3652 | 47.90511 | 50.71792 | 53.35191 |
| 741 | pos_126 | N-acetyl-L-glutamic acid | 217.7972 | 223.8976 | 240.2066 | 86.83631 | 89.65804 | 94.41215 | 69.48984 | 72.60256 | 73.47191 | 70.6695 | 76.37328 | 78.6715 |
| 742 | pos_127 | 2'-Deoxyguanosine 5'-monophosphate | 43.37283 | 43.49131 | 46.37327 | 53.59303 | 54.61217 | 57.56976 | 48.72192 | 49.93167 | 52.60601 | 49.40474 | 54.41639 | 56.73011 |
| 743 | pos_128 | L-Ala-D-Glu-meso-A2pm | 0 | 0 | 0 | 0 | 0 | 0 | 104.618 | 106.9808 | 107.2528 | 0 | 0 | 0 |
| 744 | pos_129 | Lutein | 16679.81 | 17848.15 | 19281.14 | 17891.76 | 18272.1 | 18541.83 | 17648.79 | 19048.46 | 20056.67 | 17797.69 | 18151.69 | 19378.16 |
| 745 | pos_130 | 3',5'-cyclic CMP | 128.1708 | 130.3809 | 137.0219 | 3.220858 | 3.342157 | 3.412851 | 4.697557 | 4.981382 | 5.293879 | 2.555445 | 2.599861 | 2.840643 |
| 746 | pos_131 | Flavin Mononucleotide | 54.31537 | 56.14424 | 56.27754 | 56.25344 | 56.37218 | 59.81647 | 46.72557 | 47.82357 | 48.52661 | 55.86413 | 61.33487 | 64.72204 |
| 747 | pos_132 | 4-coumaroyl-CoA | 9.591573 | 10.5486 | 11.07134 | 114.4012 | 114.808 | 117.4683 | 91.28386 | 100.2319 | 108.605 | 83.76588 | 91.76685 | 94.02029 |
| 748 | pos_133 | Lipoyl-AMP | 1094.553 | 1182.165 | 1248.744 | 1450.257 | 1462.709 | 1560.674 | 6173.845 | 6635.853 | 7058.619 | 673.1443 | 738.2092 | 738.3825 |
| 749 | pos_134 | 1,5-Decanolide | 127.4056 | 130.5235 | 134.9865 | 115.6576 | 120.2915 | 121.4151 | 111.6286 | 120.4256 | 125.4832 | 123.3859 | 131.2051 | 144.0908 |
| 750 | pos_135 | GMP-lysine | 249.3033 | 261.9264 | 281.3952 | 186.5233 | 201.4478 | 208.8194 | 210.3854 | 211.2376 | 230.6847 | 183.3328 | 190.2376 | 193.9998 |
| 751 | pos_136 | 4-(Cytidine 5'-diphospho)-2-C-methyl-D-erythritol | 22.11536 | 23.20219 | 23.61249 | 120.8941 | 130.0628 | 133.3435 | 112.8059 | 124.925 | 126.8759 | 141.3411 | 156.0134 | 163.4774 |
| 752 | pos_137 | Melibiose | 53.36881 | 56.3646 | 61.20412 | 43.84448 | 45.63029 | 46.01979 | 48.19596 | 52.15168 | 53.17176 | 44.20046 | 46.94492 | 47.98082 |
| 753 | pos_138 | PC(18:1(11Z)/18:3(9Z,12Z,15Z)) | 23395.37 | 25272.5 | 25541.5 | 14358.46 | 15595.33 | 16122.53 | 24222.82 | 25767.73 | 26273.02 | 9367.933 | 9370.639 | 9486.091 |
| 754 | pos_139 | 2,3-dihydro-2,3-dihydroxybenzoate | 83.29136 | 88.84101 | 95.42557 | 22.47522 | 23.98897 | 25.36963 | 21.48462 | 22.5127 | 23.69882 | 27.90248 | 29.98322 | 30.70115 |
| 755 | pos_140 | PC(20:4(8Z,11Z,14Z,17Z)/18:1(11Z)) | 17712.97 | 19643.88 | 20918.81 | 11425.41 | 12098.78 | 13297.33 | 15240.11 | 16792.12 | 17060.79 | 7016.175 | 7351.226 | 7542.131 |
| 756 | pos_141 | S-(Formylmethyl)glutathione | 3.348671 | 3.694541 | 3.945492 | 27.34732 | 30.2043 | 33.08698 | 2.507384 | 2.661084 | 2.888138 | 43.00113 | 44.8911 | 47.47454 |
| 757 | pos_142 | cephaloridine | 50.92471 | 52.92537 | 54.99867 | 48.68851 | 53.90534 | 56.62498 | 39.50408 | 41.26186 | 43.93102 | 60.8671 | 62.88031 | 65.8172 |
| 758 | pos_143 | N-acetyl-D-proline | 9.578914 | 9.626611 | 9.927536 | 12.48484 | 12.6344 | 13.64105 | 12.08589 | 12.67206 | 13.43197 | 12.21069 | 13.08364 | 13.32776 |
| 759 | pos_144 | Ubiquinone 4 | 767.6477 | 812.0762 | 814.7107 | 699.2433 | 773.8987 | 774.8711 | 863.5678 | 908.7414 | 923.1023 | 649.9668 | 721.579 | 759.595 |
| 760 | pos_145 | Phytosphingosine | 246.9617 | 263.4685 | 272.3622 | 249.0613 | 268.1032 | 274.6121 | 1125.158 | 1154.413 | 1166.802 | 256.9839 | 266.3085 | 287.6767 |
| 761 | pos_146 | 5-Amino-6-(5'-phosphoribosylamino)uracil | 46.72618 | 49.56013 | 54.33056 | 4.456165 | 4.861389 | 5.065339 | 7.03802 | 7.096363 | 7.801482 | 7.077923 | 7.705539 | 7.852985 |

Continued Table S2

| 762 | pos_147 | Glycyrrhyzin | 0 | 0 | 0 | 0 | 0 | 0 | 685.8966 | 689.0606 | 741.0221 | 0 | 0 | 0 |
| --- | --- | --- | --- | --- | --- | --- | --- | --- | --- | --- | --- | --- | --- | --- |
| 763 | pos_148 | UDP-N-acetylmuramoyl-L-alanine | 158.0819 | 165.1956 | 166.9775 | 133.662 | 135.0649 | 136.7551 | 158.6061 | 168.5126 | 173.7354 | 122.5814 | 124.3666 | 134.2613 |
| 764 | pos_149 | N-Succinyl-L,L-2,6-diaminopimelate | 36.16728 | 37.62722 | 40.81938 | 32.44856 | 32.81426 | 34.18329 | 177.6904 | 179.6983 | 184.5241 | 39.0051 | 40.21449 | 41.68977 |
| 765 | pos_150 | Galactose | 32.61232 | 33.63897 | 34.88779 | 33.72073 | 33.7479 | 33.80767 | 26.55995 | 29.17669 | 30.22328 | 30.87641 | 33.55432 | 36.03694 |
| 766 | pos_151 | (1R,6R)-6-Hydroxy-2-succinylcyclohexa-2,4-diene-1-carboxylate | 53.42022 | 58.66871 | 59.88535 | 223.5354 | 232.2498 | 239.0065 | 343.688 | 354.5784 | 383.6982 | 351.1407 | 363.8376 | 386.3746 |
| 767 | pos_152 | 4-Phosphopantothenoylcysteine | 69.06433 | 69.14795 | 75.89478 | 12.88625 | 14.16658 | 14.17039 | 41.66919 | 43.16722 | 44.13868 | 4.97411 | 4.975436 | 5.40945 |
| 768 | pos_153 | L-Histidine | 9579.501 | 9773.958 | 10497.79 | 9697.963 | 9871.358 | 10325.38 | 8943.784 | 9742.54 | 10006.73 | 9505.454 | 10099.03 | 10170.26 |
| 769 | pos_154 | Nicotinamide ribotide | 203.0019 | 216.7322 | 228.2122 | 31.31785 | 32.84596 | 33.7466 | 17.92051 | 18.63763 | 20.25703 | 26.65929 | 27.55609 | 29.94835 |
| 770 | pos_155 | Uridine | 47.95016 | 50.10429 | 54.69216 | 15.70555 | 15.76422 | 16.84152 | 13.44312 | 13.79048 | 13.9061 | 13.32539 | 14.77644 | 16.21804 |
| 771 | pos_156 | Tetrahydrobiopterin | 401.5184 | 444.0199 | 447.1821 | 516.7226 | 531.5591 | 537.1166 | 496.4797 | 537.5596 | 567.8673 | 524.0876 | 534.1132 | 564.5444 |
| 772 | pos_157 | 1-Methylnicotinamide | 1339.759 | 1457.483 | 1514.307 | 656.856 | 669.5726 | 716.5923 | 1226.114 | 1240.487 | 1361.292 | 608.4972 | 665.2611 | 679.3999 |
| 773 | pos_158 | AICAR | 38.44366 | 41.22928 | 44.87738 | 20.68363 | 21.8734 | 22.27967 | 84.34908 | 89.63767 | 89.98571 | 0.802288 | 0.878327 | 0.915969 |
| 774 | pos_159 | Oleic acid | 327.7533 | 344.0565 | 349.4497 | 353.1536 | 373.8017 | 406.7671 | 273.9625 | 283.3019 | 284.9848 | 403.1198 | 404.6463 | 417.1336 |
| 775 | pos_160 | IPC 18:0;2/20:0;0 | 4871.329 | 5312.456 | 5445.131 | 4747.764 | 4998.494 | 5168.031 | 5093.765 | 5304.762 | 5677.097 | 4823.521 | 5229.303 | 5706.171 |
| 776 | pos_161 | Deoxyuridine | 20.97587 | 22.88652 | 24.20452 | 699.8729 | 722.426 | 724.5408 | 548.3975 | 582.4137 | 588.9186 | 501.2347 | 553.7396 | 598.2842 |
| 777 | pos_162 | 2-aminogalactopyranose | 15.64285 | 16.57497 | 16.5977 | 14.31641 | 15.8271 | 16.58989 | 152.2426 | 160.9369 | 163.0426 | 8.370381 | 8.460892 | 8.552186 |
| 778 | pos_163 | 1-Nitro-7-hydroxy-8-glutathionyl-7,8-dihydronaphthalene | 14.51268 | 14.66678 | 15.87577 | 31.64096 | 32.39059 | 34.33023 | 8.909376 | 9.386492 | 9.921801 | 29.18519 | 31.07594 | 33.01769 |
| 779 | pos_164 | Megastigmatrienone | 64.70249 | 68.47621 | 74.77714 | 92.02571 | 92.59679 | 93.91444 | 92.65873 | 98.37587 | 100.8987 | 92.52737 | 101.181 | 106.0851 |
| 780 | pos_165 | L-Leucine | 748.4536 | 769.581 | 846.336 | 63.34716 | 64.17474 | 65.71821 | 144.054 | 150.5642 | 152.7992 | 41.93526 | 43.99419 | 44.7097 |
| 781 | pos_166 | S-adenosyl-L-(2 hydroxyl-4-methylthio)butyrate | 216.9609 | 221.4078 | 228.9402 | 273.0426 | 292.7132 | 306.7152 | 319.7407 | 329.0941 | 355.6563 | 271.0929 | 276.7513 | 278.1527 |
| 782 | pos_167 | D-Phenylalanine | 188.2695 | 208.4835 | 219.9951 | 122.6595 | 135.9678 | 146.2264 | 102.5765 | 102.9423 | 104.7162 | 117.8775 | 125.4855 | 132.7193 |
| 783 | pos_168 | S-Acetyldihydrolipoamide | 1135.974 | 1182.418 | 1290.101 | 1187.774 | 1217.258 | 1292.849 | 1124.857 | 1205.341 | 1226.438 | 1135.844 | 1222.777 | 1269.066 |
| 784 | pos_169 | CDP-DG(18:0/22:3(10Z,13Z,16Z)) | 172.9852 | 175.328 | 181.8759 | 180.7406 | 183.5154 | 200.8068 | 212.5545 | 228.3737 | 239.4417 | 283.6763 | 286.6697 | 291.0795 |

Continued Table S2

| 785 | pos_170 | Biotinyl-5'-AMP | 85.03494 | 85.55325 | 93.08933 | 7.528703 | 8.350023 | 8.363173 | 47.27296 | 51.03529 | 54.00353 | 2.477089 | 2.639549 | 2.752794 |
| --- | --- | --- | --- | --- | --- | --- | --- | --- | --- | --- | --- | --- | --- | --- |
| 786 | pos_171 | UDP-N-acetylmuramoyl-L-alanyl-D-glutamate | 0 | 0 | 0 | 0 | 0 | 0 | 0 | 0 | 0 | 1.791359 | 1.947564 | 2.113826 |
| 787 | pos_172 | Tetrahydropteridine | 57.64496 | 63.9787 | 65.85757 | 77.63141 | 78.19715 | 83.53875 | 65.18883 | 67.25047 | 68.52433 | 66.75363 | 72.70843 | 74.1281 |
| 788 | pos_173 | L-Isoleucine | 109.5682 | 118.8357 | 125.9501 | 144.5613 | 155.8935 | 160.3663 | 155.4327 | 167.996 | 182.901 | 209.4777 | 228.6744 | 250.3725 |
| 789 | pos_174 | dihydro-10-thiopteroate | 240.513 | 257.9715 | 278.1551 | 217.9384 | 226.0025 | 234.2682 | 263.8251 | 269.4786 | 295.9372 | 310.423 | 326.5966 | 345.2384 |
| 790 | pos_175 | benzylguanidine | 1408.685 | 1446.644 | 1475.686 | 1332.483 | 1342.924 | 1384.947 | 1319.26 | 1460.131 | 1462.079 | 1408.168 | 1534.446 | 1534.619 |
| 791 | pos_176 | 5'-Phosphoribosyl-N-formylglycinamide | 56.2496 | 61.20608 | 66.12851 | 59.10204 | 60.12719 | 64.31803 | 5.457331 | 5.471042 | 5.644594 | 14.60144 | 15.84638 | 16.8826 |
| 792 | pos_177 | PS(16:0/16:0) | 13336.3 | 13892.15 | 14631.66 | 12276.71 | 13251.09 | 13927.12 | 12478.63 | 13174.4 | 14369.61 | 12398.02 | 12639.98 | 13831.49 |
| 793 | pos_178 | N1-(alpha-D-ribosyl)-5,6-dimethyl-benzimidazole | 113.9157 | 115.3287 | 116.8211 | 106.5071 | 116.9084 | 121.464 | 104.58 | 115.6474 | 118.4838 | 107.4867 | 108.0508 | 113.5742 |
| 794 | pos_179 | ethyl-(2R)-methyl-(3S)-hydroxybutanoate | 682.5647 | 749.0491 | 810.6799 | 135.1518 | 142.1581 | 145.5782 | 59.08397 | 61.24948 | 63.71758 | 42.50789 | 47.02537 | 49.73604 |
| 795 | pos_180 | Trehalose 6-phosphate | 28.09873 | 28.85918 | 31.55963 | 10.27757 | 11.27273 | 11.6107 | 15.60492 | 16.53614 | 16.59763 | 8.663957 | 9.624877 | 9.819838 |
| 796 | pos_181 | D-galactal | 124.9697 | 137.3845 | 139.8656 | 0.080759 | 0.087498 | 0.089664 | 70.03022 | 76.29836 | 80.62194 | 0.019508 | 0.021458 | 0.021492 |
| 797 | pos_182 | Guanosine diphosphate | 96.1146 | 99.64478 | 106.9104 | 8.187447 | 8.924936 | 9.534491 | 7.366877 | 8.012663 | 8.401928 | 7.414757 | 8.046628 | 8.438101 |
| 798 | pos_183 | 5'-deoxyuridine | 0.026952 | 0.027853 | 0.030118 | 87.8456 | 97.54906 | 104.1615 | 1.087249 | 1.091623 | 1.131735 | 27.76082 | 30.6282 | 30.74937 |
| 799 | pos_184 | DIMBOA-Glc | 130.0731 | 133.2916 | 146.0294 | 34.72512 | 36.51516 | 37.48959 | 34.8505 | 35.79649 | 37.52793 | 27.47718 | 28.44367 | 29.37896 |
| 800 | pos_185 | Peonidin 3-O-(acetylglucoside) | 112.3503 | 119.381 | 125.681 | 400.1794 | 408.0832 | 438.7929 | 176.863 | 186.5164 | 193.9074 | 367.6651 | 402.9094 | 434.5422 |
| 801 | pos_186 | menaquinone-1 | 136.9141 | 147.0135 | 152.1239 | 85.18591 | 91.55444 | 93.6758 | 109.9786 | 119.0912 | 130.4941 | 98.14634 | 102.9963 | 113.1763 |
| 802 | pos_187 | Geranyl acetone | 3663.426 | 3847.044 | 4192.699 | 4361.967 | 4487.605 | 4505.922 | 3355.342 | 3355.994 | 3682.374 | 4550.922 | 5050.862 | 5353.951 |
| 803 | pos_188 | Raffinose | 23.39762 | 24.15838 | 24.831 | 0 | 0 | 0 | 152.3908 | 156.4176 | 162.4295 | 0 | 0 | 0 |
| 804 | pos_189 | dTDP-4-acetamido-4,6-dideoxy-D-galactose | 963.2401 | 989.645 | 1039.817 | 170.2685 | 186.0931 | 193.6114 | 164.7612 | 180.8174 | 198.0626 | 187.1468 | 199.0699 | 205.2213 |
| 805 | pos_190 | UDP-N-acetylmuraminate | 78.87039 | 86.47462 | 95.06423 | 20.37725 | 20.67578 | 21.61152 | 25.12726 | 26.71102 | 27.16368 | 24.22994 | 24.48979 | 26.77315 |
| 806 | pos_191 | 2'-Deoxyuridine | 426.9476 | 437.8403 | 443.6704 | 320.7929 | 323.8625 | 355.0016 | 351.264 | 360.8706 | 386.7471 | 258.8911 | 286.3429 | 311.806 |
| 807 | pos_192 | 2,3-Bis(3-hydroxytetradecanoyl)-beta-D-glucosaminyl 1-phosphate | 3276.964 | 3638.867 | 3705.824 | 3863.801 | 3984.18 | 4008.7 | 2466.662 | 2684.209 | 2738.811 | 4186.95 | 4309.68 | 4485.59 |

Continued Table S2

| 808 | pos_193 | N-Acetyl-D-tyrosine | 13.04202 | 14.22325 | 15.49873 | 27.52019 | 28.26418 | 29.60975 | 25.69792 | 26.74855 | 27.63944 | 20.91444 | 21.87702 | 21.93579 |
| --- | --- | --- | --- | --- | --- | --- | --- | --- | --- | --- | --- | --- | --- | --- |
| 809 | pos_194 | Vitisin B | 37.00389 | 40.55933 | 41.45306 | 2.374261 | 2.558933 | 2.685179 | 0 | 0 | 0 | 0 | 0 | 0 |
| 810 | pos_195 | Methyl succinate | 165.2271 | 176.7659 | 188.2793 | 16.51355 | 18.13664 | 19.59576 | 26.09795 | 28.40164 | 29.96609 | 6.886763 | 7.437605 | 7.733608 |
| 811 | pos_196 | L-Proline | 1224.105 | 1307 | 1368.328 | 68.56843 | 74.23872 | 74.75782 | 224.5161 | 232.0412 | 247.21 | 64.14693 | 65.33812 | 69.14326 |
| 812 | pos_197 | Delphinidin | 83.07784 | 91.13034 | 93.26291 | 172.467 | 178.7305 | 186.6096 | 217.2427 | 232.1075 | 239.6782 | 159.1685 | 161.1319 | 169.5001 |
| 813 | pos_198 | D-Galacturonate | 6.898777 | 7.010967 | 7.109661 | 55.1415 | 57.54595 | 60.8869 | 33.70015 | 34.34696 | 36.08941 | 50.4029 | 55.28524 | 56.59424 |
| 814 | pos_199 | 2,5-Diamino-4-hydroxy-6-(5-phosphoribosylamino)pyrimidine | 172.5313 | 174.4576 | 177.2461 | 46.01569 | 47.6992 | 52.06941 | 58.88166 | 60.13591 | 60.62962 | 60.87611 | 64.75723 | 66.72154 |
| 815 | pos_200 | Guanosine 2',3'-cyclic phosphate | 135.4368 | 137.5651 | 138.4589 | 24.83497 | 27.13401 | 28.69784 | 26.24138 | 26.55605 | 29.17153 | 25.62513 | 27.35729 | 29.27559 |
| 816 | pos_201 | Saccharopine | 266.7269 | 281.0242 | 291.9264 | 197.3619 | 219.1612 | 230.8556 | 194.4868 | 204.7013 | 220.8446 | 227.8084 | 238.7682 | 248.9034 |
| 817 | pos_202 | 4-Hydroxy-L-threonine | 188.2737 | 191.4207 | 194.864 | 339.8582 | 361.2133 | 387.3332 | 281.5037 | 299.1178 | 303.0392 | 349.9159 | 379.9199 | 398.6933 |
| 818 | pos_203 | 4-methylumbelliferone | 468.0792 | 498.6557 | 517.3889 | 489.9464 | 511.1556 | 533.7864 | 470.8915 | 483.2898 | 491.9949 | 477.7158 | 484.5907 | 524.7994 |
| 819 | pos_204 | amiclenomycin | 103.6557 | 112.3363 | 119.1857 | 118.3839 | 129.8161 | 142.0864 | 117.9306 | 121.5598 | 124.9464 | 99.54716 | 107.4248 | 113.3954 |
| 820 | pos_205 | S-tubercidinylhomocysteine | 560.5206 | 613.6079 | 654.6447 | 963.1334 | 1065.429 | 1101.105 | 1241.98 | 1351.225 | 1458.042 | 923.5189 | 988.1556 | 1058.736 |
| 821 | pos_206 | Kaempferol | 7784.067 | 7915.488 | 8129.185 | 48.76011 | 53.31026 | 58.41285 | 96.91285 | 99.76133 | 100.7874 | 113.5529 | 115.9083 | 124.2503 |
| 822 | pos_207 | dGDP | 78.03416 | 78.29372 | 82.44085 | 56.27012 | 56.41729 | 61.37357 | 53.9805 | 57.60509 | 58.41229 | 45.30732 | 50.04512 | 50.26696 |
| 823 | pos_208 | Ureidoisobutyric acid | 970.5811 | 1058.12 | 1108.595 | 143.0215 | 148.7002 | 156.5735 | 235.4562 | 253.6031 | 272.6117 | 129.483 | 143.3341 | 146.5271 |
| 824 | pos_209 | 5-Phenyl-1,3-oxazinane-2,4-dione | 191.4385 | 193.9168 | 211.3426 | 12.68924 | 13.34406 | 14.00233 | 32.4433 | 33.92303 | 34.79293 | 12.52929 | 12.98257 | 13.27915 |
| 825 | pos_210 | Cysteinylglycine | 163.637 | 172.1235 | 185.9133 | 56.08809 | 56.62995 | 59.34809 | 47.6505 | 48.67702 | 49.58639 | 50.77834 | 53.48155 | 54.42795 |
| 826 | pos_211 | Propinol adenylate | 49.73366 | 53.9087 | 58.52587 | 64.6511 | 65.22868 | 68.05612 | 69.41775 | 72.86798 | 72.9373 | 78.5084 | 85.42058 | 86.81967 |
| 827 | pos_212 | N-Acetylornithine | 42.92083 | 47.24566 | 51.82921 | 7.68334 | 7.959663 | 8.229982 | 8.426356 | 8.877287 | 9.392169 | 13.04614 | 13.31342 | 14.46555 |
| 828 | pos_213 | PI(14:1(9Z)/0:0) | 463.3293 | 478.2557 | 499.1931 | 527.55 | 557.5464 | 568.9162 | 552.7393 | 586.0321 | 588.2553 | 682.131 | 694.6651 | 742.2807 |
| 829 | pos_214 | 9-[6(RS)-8-diamino-5,6,7,8-tetradeoxy-beta-D-ribo-octofuranosyl]-9H-purin-6-amine | 0 | 0 | 0 | 0 | 0 | 0 | 0 | 0 | 0 | 0 | 0 | 0 |
| 830 | pos_215 | 5'-amino-5'-deoxyuridine | 73.3431 | 80.60423 | 83.80414 | 5.506978 | 5.526118 | 5.942357 | 20.36963 | 21.61063 | 23.15146 | 16.80121 | 18.09258 | 19.42584 |
| 831 | pos_216 | Melibiitol | 0.126188 | 0.13967 | 0.143387 | 78.2684 | 78.73481 | 79.13088 | 80.89983 | 85.59595 | 85.59833 | 92.46083 | 92.78756 | 96.0799 |

Continued Table S2

| 832 | pos_217 | N3-(4-methoxyfumaroyl)-L-2,3-diaminopropanoate | 477.1847 | 491.5166 | 516.3484 | 464.5706 | 498.6533 | 505.1226 | 497.7619 | 512.7235 | 545.4893 | 444.2611 | 492.9222 | 497.1752 |
| --- | --- | --- | --- | --- | --- | --- | --- | --- | --- | --- | --- | --- | --- | --- |
| 833 | pos_218 | Allysine | 150.1175 | 160.6917 | 168.0452 | 127.7708 | 134.7671 | 143.5966 | 126.1693 | 137.9727 | 140.43 | 136.8419 | 140.5133 | 154.4646 |
| 834 | pos_219 | (N(omega)-L-arginino)succinic acid | 707.2677 | 770.2545 | 776.5556 | 2680.649 | 2942.903 | 3015.243 | 1099.491 | 1139.381 | 1139.697 | 2804.088 | 3063.681 | 3367.812 |
| 835 | pos_220 | N1-methyladenine | 36.5425 | 40.51345 | 40.82616 | 65.95167 | 71.43264 | 75.13344 | 54.29818 | 55.4746 | 59.51079 | 72.33866 | 78.15758 | 85.27439 |
| 836 | pos_221 | dcSAM | 0 | 0 | 0 | 38.16528 | 42.20984 | 44.9532 | 0 | 0 | 0 | 161.7554 | 175.813 | 178.0165 |
| 837 | pos_222 | N5-Carboxyaminoimidazole ribonucleotide | 3.459421 | 3.521096 | 3.708177 | 6.432674 | 6.770102 | 7.353119 | 3.024821 | 3.17432 | 3.304015 | 5.92947 | 6.540441 | 6.947574 |
| 838 | pos_223 | Petunidin 3-O-(acetylglucoside) | 194.4397 | 201.334 | 210.2295 | 16.79605 | 17.53368 | 17.64094 | 12.19177 | 12.98958 | 14.20463 | 8.711386 | 9.314603 | 9.979098 |
| 839 | pos_224 | 5'-Deoxyadenosine | 155.3354 | 155.983 | 163.062 | 153.3486 | 157.7884 | 168.5023 | 144.4192 | 146.7583 | 161.3841 | 152.6336 | 154.4451 | 161.7678 |
| 840 | pos_225 | PC(14:0/20:4(8Z,11Z,14Z,17Z)) | 15025.06 | 16042.07 | 16251.86 | 14411.08 | 15901.58 | 16524.91 | 14197.94 | 14866.65 | 14980.79 | 14374.12 | 15818.41 | 16916.55 |
| 841 | pos_226 | L-Ala-gamma-D-Glu-DAP-D-Ala | 255.5625 | 278.62 | 284.761 | 388.2753 | 393.7775 | 432.8708 | 182347.1 | 197459.6 | 198910.3 | 280.9781 | 303.4359 | 308.4829 |
| 842 | pos_227 | 3-Deoxy-D-manno-octulosonate 8-phosphate | 0 | 0 | 0 | 34.7319 | 34.80294 | 35.65931 | 4.533046 | 4.863655 | 5.015069 | 40.01947 | 42.07873 | 43.68371 |
| 843 | pos_228 | dGMP | 0 | 0 | 0 | 10.94708 | 11.90646 | 12.82948 | 13.58162 | 13.84948 | 15.21111 | 11.40838 | 11.95222 | 12.25229 |
| 844 | pos_229 | Vanillin | 399.1987 | 441.6611 | 485.7106 | 29.32517 | 31.16747 | 31.19004 | 39.56162 | 40.72841 | 44.4319 | 33.63412 | 35.14216 | 36.62583 |
| 845 | pos_230 | Benzoyl-CoA | 44.80526 | 44.8921 | 45.10029 | 279.5132 | 307.0935 | 308.4543 | 396.246 | 399.8733 | 438.2348 | 234.0773 | 247.8491 | 271.8027 |
| 846 | pos_231 | Phenylacetyl-CoA | 0 | 0 | 0 | 28.01977 | 29.60112 | 31.88823 | 37.3686 | 37.40907 | 37.62815 | 29.27786 | 30.12698 | 31.6491 |
| 847 | pos_232 | 4-Aminobutyraldehyde | 187.2139 | 195.5676 | 209.6417 | 2.003531 | 2.121071 | 2.238687 | 23.41125 | 25.72628 | 27.07099 | 5.218055 | 5.384353 | 5.652079 |
| 848 | pos_233 | Coenzyme A | 149.2462 | 150.7837 | 160.9068 | 160.5524 | 172.9237 | 187.1491 | 101.9908 | 104.0894 | 110.6457 | 110.3381 | 118.3509 | 129.4285 |
| 849 | pos_234 | 3-beta-Hydroxy-4-beta-methyl-5-alpha-cholest-7-ene-4-alpha-carboxylate | 376.5157 | 408.0855 | 440.1793 | 460.3898 | 489.8713 | 535.1436 | 457.3759 | 461.5854 | 488.7752 | 683.6614 | 728.2372 | 782.2954 |
| 850 | pos_235 | 2,3-pentane-dione | 51.09155 | 51.10731 | 53.21812 | 53.51812 | 57.45964 | 60.99925 | 47.84427 | 47.96538 | 47.99232 | 50.22063 | 55.75071 | 60.37215 |
| 851 | pos_236 | 2-Methoxy-3-isobutylpyrazine | 6659.989 | 7206.659 | 7549.958 | 6221.201 | 6873.487 | 7341.563 | 7248.632 | 7363.079 | 7680.844 | 6580.549 | 6929.297 | 7564.773 |
| 852 | pos_237 | octyl alpha-D-glucopyranoside | 1169.891 | 1259.878 | 1301.357 | 1361.064 | 1408.167 | 1528.989 | 1291.793 | 1294.837 | 1410.755 | 1179.71 | 1285.087 | 1309.58 |
| 853 | pos_238 | 5'-(p-nitrophenyl)thioadenosine | 44.43275 | 47.29543 | 49.0395 | 81.04778 | 86.51991 | 88.93904 | 19.39052 | 21.12513 | 22.41848 | 89.50107 | 97.30032 | 103.8346 |
| 854 | pos_239 | Nicotinate D-ribonucleoside | 302.9379 | 321.6237 | 342.3593 | 1192.135 | 1323.292 | 1408.836 | 1719.19 | 1722.429 | 1776.631 | 730.0891 | 781.3179 | 793.9173 |
| 855 | pos_240 | 5-Aminoimidazole ribonucleotide | 239.0102 | 239.8149 | 262.0177 | 101.5828 | 112.3688 | 119.4069 | 101.0477 | 105.667 | 115.0096 | 84.9649 | 92.18845 | 100.3634 |

Continued Table S2

| 856 | pos_241 | 4,5-Dihydroorotic acid | 37.43537 | 38.96069 | 41.12632 | 12.54022 | 13.77576 | 15.08534 | 22.48603 | 24.36308 | 25.79677 | 12.08499 | 12.8637 | 13.22261 |
| --- | --- | --- | --- | --- | --- | --- | --- | --- | --- | --- | --- | --- | --- | --- |
| 857 | pos_242 | thiabendazole | 54611.72 | 55997.62 | 57529.44 | 57354.04 | 57488.96 | 62005.06 | 58088.74 | 58403.82 | 58920.91 | 53180.56 | 56523.27 | 61765.2 |
| 858 | pos_243 | (S)-b-aminoisobutyric acid | 0 | 0 | 0 | 31.50827 | 34.17169 | 37.27483 | 5.415343 | 5.935158 | 6.395092 | 20.36883 | 21.79352 | 23.25412 |
| 859 | pos_244 | 4-Hydroxyphenylacetaldehyde | 5319.053 | 5519.825 | 5979.195 | 1608.212 | 1661.342 | 1819.581 | 1558.782 | 1694.911 | 1810.412 | 1437.381 | 1548.182 | 1695.608 |
| 860 | pos_245 | S-Adenosylhomocysteine | 1229.854 | 1230.517 | 1294.283 | 243.3278 | 251.0621 | 251.7757 | 755.9395 | 797.0485 | 871.6487 | 238.303 | 242.71 | 257.5903 |
| 861 | pos_246 | 2-(6-Hydroxy-2-methoxy-3,4-methylenedioxyphenyl)benzofuran | 277.8834 | 279.235 | 286.5535 | 270.5123 | 286.6876 | 301.15 | 515.6246 | 518.5282 | 521.9264 | 223.1595 | 225.0453 | 246.9211 |
| 862 | pos_247 | gamma-Glutamyl-beta-cyanoalanine | 0.417807 | 0.440147 | 0.448779 | 0.337159 | 0.347112 | 0.350394 | 88.20988 | 95.68559 | 99.90756 | 0.53801 | 0.563725 | 0.58 |
| 863 | pos_248 | PC(20:5(5Z,8Z,11Z,14Z,17Z)/P-18:1(11Z)) | 26585.49 | 28493.59 | 28736.89 | 20869.12 | 21254.6 | 23023.49 | 24368.38 | 25453.5 | 27942.68 | 13677.57 | 13838.13 | 14042.52 |
| 864 | pos_249 | PI(12:0/16:1(9Z)) | 13.5248 | 13.96999 | 14.71865 | 111.0404 | 121.711 | 124.9482 | 40.04879 | 43.12278 | 46.30606 | 161.0027 | 173.5644 | 177.5975 |
| 865 | pos_250 | N-Acetylmuramic acid 6-phosphate | 16.7661 | 17.03846 | 18.04905 | 0 | 0 | 0 | 4.135096 | 4.468272 | 4.764581 | 0 | 0 | 0 |
| 866 | pos_251 | Petunidin | 309.5599 | 309.857 | 318.7377 | 462.6836 | 472.0072 | 500.566 | 369.962 | 393.9232 | 398.0475 | 593.0282 | 597.9191 | 603.517 |
| 867 | pos_252 | Thiamine monophosphate | 34304.44 | 35183.49 | 38245.37 | 37.71294 | 41.08108 | 41.59566 | 48.04466 | 48.33349 | 49.95214 | 45.62147 | 47.28449 | 49.24404 |
| 868 | pos_253 | Novobiocin | 363.3854 | 368.9016 | 404.8029 | 242.2659 | 265.601 | 289.6323 | 215.4991 | 233.7618 | 247.5719 | 213.7265 | 219.0593 | 224.612 |
| 869 | pos_254 | 5'-amino-5'-deoxyadenosine | 1372.136 | 1522.417 | 1554.746 | 14.39941 | 15.21889 | 15.78424 | 25.1143 | 27.79497 | 29.61316 | 11.74205 | 12.06323 | 12.22527 |
| 870 | pos_255 | PC(18:4(6Z,9Z,12Z,15Z)/24:0) | 421.3544 | 457.42 | 495.7366 | 382.9026 | 383.7142 | 412.101 | 385.0019 | 416.0474 | 430.3038 | 347.5192 | 365.1124 | 394.4405 |
| 871 | pos_256 | (2S,3S,4R)-2-aminoicosane-1,3,4-triol | 3323.963 | 3482.625 | 3658.417 | 3132.511 | 3472.726 | 3615.245 | 3405.269 | 3427.331 | 3598.445 | 3540.895 | 3545.375 | 3833.767 |
| 872 | pos_257 | 2-Aminobenzoic acid | 59.32917 | 63.04512 | 64.88971 | 144.7969 | 155.277 | 168.4963 | 70.14349 | 73.13911 | 74.69413 | 249.9545 | 273.7246 | 299.4307 |
| 873 | pos_258 | CDP-Ethanolamine | 88.25029 | 93.55508 | 102.5293 | 33.57053 | 35.27062 | 38.31522 | 32.55505 | 35.00086 | 37.61156 | 37.64672 | 38.02832 | 39.37717 |
| 874 | pos_259 | 5-Thymidylic acid | 363.5216 | 367.8245 | 400.0689 | 205.9748 | 228.5505 | 249.3221 | 231.3533 | 241.3369 | 244.4905 | 281.2714 | 286.1925 | 310.8859 |
| 875 | pos_260 | 4,6-Dideoxy-4-oxo-dTDP-D-glucose | 2551.313 | 2588.17 | 2734.803 | 1284.519 | 1305.366 | 1306.878 | 1408.159 | 1430.041 | 1460.221 | 997.0203 | 1087.197 | 1132.06 |
| 876 | pos_261 | Nonanoic acid | 1007.911 | 1091.421 | 1140.32 | 1164.795 | 1187.049 | 1224.695 | 875.6127 | 893.918 | 941.3815 | 1175.523 | 1298.649 | 1343.457 |
| 877 | pos_262 | Ethyl 7-octenoate | 145.0888 | 146.0983 | 157.9183 | 148.0167 | 150.0346 | 163.9772 | 143.0734 | 154.1311 | 168.7792 | 143.1574 | 144.4527 | 144.7551 |
| 878 | pos_263 | CMP-3-deoxy-D-manno-octulosonate | 41.78797 | 42.54559 | 44.31827 | 26.7578 | 29.10935 | 30.9805 | 15.57384 | 16.02588 | 17.1663 | 28.04215 | 28.8361 | 30.86766 |
| 879 | pos_264 | Uridine diphosphate-N-acetylglucosamine | 487.7975 | 509.1724 | 518.3429 | 523.0675 | 557.802 | 580.9887 | 589.4287 | 608.1865 | 662.3952 | 553.6442 | 608.0722 | 644.0696 |
| 880 | pos_265 | Indole | 91492.39 | 101537.9 | 108964.8 | 92558.7 | 100673.5 | 103408.3 | 92393.37 | 99417.44 | 105151.4 | 93629.92 | 97856.39 | 104553 |

Continued Table S2

| 881 | pos_266 | Lipoic acid | 157.156 | 166.0188 | 180.7703 | 0 | 0 | 0 | 0 | 0 | 0 | 0 | 0 | 0 |
| --- | --- | --- | --- | --- | --- | --- | --- | --- | --- | --- | --- | --- | --- | --- |
| 882 | pos_267 | N2-Succinyl-L-arginine | 0 | 0 | 0 | 8.110192 | 8.149418 | 8.528204 | 0 | 0 | 0 | 1.583501 | 1.610357 | 1.634181 |
| 883 | pos_268 | Carvone | 266.8082 | 293.2518 | 315.7533 | 270.1617 | 282.7367 | 289.051 | 288.1185 | 295.3507 | 324.8007 | 292.9873 | 294.9101 | 317.2301 |
| 884 | pos_269 | 5'-O-[N-(L-Aspartyl)sulfamoyl]adenosine | 0 | 0 | 0 | 64.06362 | 69.76727 | 70.52649 | 72.40611 | 76.08024 | 80.18161 | 72.96794 | 78.06869 | 79.96278 |
| 885 | pos_270 | LPA(16:0/0:0) | 13983.61 | 14581.16 | 15229.65 | 13855.35 | 14955.92 | 14997.15 | 13546.93 | 13988.44 | 14167.34 | 13923.93 | 15052.88 | 16519.94 |
| 886 | pos_271 | Glutathionylspermidine | 205.4847 | 212.8054 | 232.9144 | 233.7477 | 254.9485 | 274.8161 | 238.7583 | 249.5785 | 266.382 | 314.8306 | 348.1426 | 349.1576 |
| 887 | pos_272 | 3,4-Dihydroxyphenylacetaldehyde | 199.4976 | 204.0892 | 212.7664 | 25.40695 | 25.86523 | 27.00457 | 72.72354 | 75.2201 | 77.53824 | 30.74585 | 30.84852 | 32.29432 |
| 888 | pos_273 | (Hydroxymethylphenyl)succinyl-CoA | 64.33143 | 65.24622 | 69.34004 | 28.8912 | 31.63087 | 32.01323 | 35.13787 | 38.46649 | 39.74218 | 29.12082 | 32.13379 | 33.5014 |
| 889 | pos_274 | FAD | 58.10302 | 60.83171 | 62.49948 | 63.81451 | 64.2234 | 68.43233 | 63.30057 | 67.54829 | 71.71032 | 78.1829 | 83.09641 | 90.10301 |
| 890 | pos_275 | S-(2-Chloroacetyl)glutathione | 570.2402 | 594.571 | 613.7113 | 345.7719 | 353.8688 | 362.7134 | 404.0787 | 408.4332 | 423.5725 | 202.2346 | 223.0627 | 224.0343 |
| 891 | pos_276 | Ethyl lactate | 411.9734 | 442.6301 | 485.0788 | 39.48235 | 43.42433 | 45.09612 | 102.4604 | 103.2297 | 106.6744 | 33.73903 | 34.64925 | 34.90597 |
| 892 | pos_277 | Oxidized glutathione | 3453.059 | 3518.614 | 3768.694 | 2972.516 | 3254.117 | 3265.765 | 2483.486 | 2489.087 | 2646.984 | 3135.085 | 3396.998 | 3736.144 |
| 893 | pos_278 | DL-Pipecolic acid | 3700.157 | 3707.676 | 3804.505 | 8132.747 | 8638.081 | 8715.271 | 5858.647 | 6026.982 | 6207.561 | 9467.592 | 10510.53 | 10719.72 |
| 894 | pos_279 | 2,5-Diamino-6-(5-phosphono)ribitylamino-4(3H)-pyrimidinone | 87.94901 | 94.31219 | 99.14253 | 399.7566 | 428.6564 | 453.817 | 131.9871 | 143.2478 | 144.0544 | 843.8053 | 889.9771 | 959.5325 |
| 895 | pos_280 | 2'-Deoxycytidine | 109.7526 | 114.025 | 116.3444 | 111.5344 | 123.8983 | 131.7463 | 120.8798 | 121.4279 | 122.691 | 107.1599 | 113.2009 | 113.4169 |
| 896 | pos_281 | Butyric acid | 340.5918 | 355.6749 | 362.8028 | 326.4306 | 352.8221 | 382.28 | 369.0239 | 391.1007 | 392.0567 | 330.5902 | 357.5978 | 379.1498 |
| 897 | pos_282 | 4-alpha-Methyl-5-alpha-cholest-7-en-3-one | 420.604 | 429.7291 | 464.8888 | 425.5641 | 436.2021 | 479.5347 | 6158.553 | 6563.056 | 6698.003 | 792.4197 | 852.5473 | 858.9329 |
| 898 | pos_283 | Methyl cinnamate | 48.25405 | 51.49638 | 54.73952 | 8.443679 | 8.443983 | 9.224524 | 1.476889 | 1.636379 | 1.735947 | 7.062224 | 7.437885 | 7.593126 |
| 899 | pos_284 | 5'-methylthiotubercidin | 49.2246 | 52.47644 | 53.40897 | 45.75764 | 47.63656 | 50.55265 | 59.46143 | 62.36136 | 65.12646 | 39.90004 | 42.97692 | 45.23679 |
| 900 | pos_285 | 5-Hydroxylysine | 101.6095 | 102.8537 | 105.6361 | 113.8055 | 114.2438 | 117.4046 | 95.53487 | 99.79332 | 104.4615 | 63.24771 | 64.28083 | 64.3116 |
| 901 | pos_286 | Pantetheine 4'-phosphate | 341.6831 | 371.0425 | 403.0282 | 185.2345 | 193.6065 | 198.3516 | 188.3909 | 203.9857 | 221.9107 | 209.5169 | 211.3468 | 230.6731 |
| 902 | pos_287 | Phosphoribosyl formamidocarboxamide | 0 | 0 | 0 | 35.34949 | 35.49747 | 37.57725 | 3.254904 | 3.366892 | 3.486002 | 28.10985 | 30.98929 | 33.31644 |
| 903 | pos_288 | indolmycin | 1947.589 | 2084.919 | 2230.765 | 3955.471 | 4361.638 | 4396.586 | 3626.461 | 3687.034 | 3887.158 | 4049.537 | 4386.85 | 4633.739 |
| 904 | pos_289 | L-4-Hydroxyglutamate semialdehyde | 11548.31 | 11624.76 | 11688.97 | 1584.879 | 1613.418 | 1760.884 | 8621.096 | 9058.98 | 9585.625 | 1086.256 | 1104.575 | 1158.997 |
| 905 | pos_290 | S-Lactoylglutathione | 24.44952 | 27.06716 | 27.86298 | 110.06 | 118.1146 | 121.027 | 105.2867 | 116.3739 | 118.2299 | 117.9488 | 123.5079 | 127.3853 |

Continued Table S2

| 906 | pos_291 | Orotic acid | 21.25151 | 23.60719 | 23.6213 | 3.550992 | 3.5849 | 3.616555 | 3.273056 | 3.443825 | 3.711344 | 8.765944 | 9.037567 | 9.680699 |
| --- | --- | --- | --- | --- | --- | --- | --- | --- | --- | --- | --- | --- | --- | --- |
| 907 | pos_292 | 1,1,6-Trimethyl-1,2-dihydronaphthalene | 839.754 | 894.3718 | 905.7334 | 808.9987 | 886.8219 | 912.4892 | 929.79 | 978.4452 | 1024.334 | 720.1235 | 785.6495 | 863.0484 |
| 908 | pos_293 | PE(18:0/0:0) | 170.35 | 175.5218 | 186.2117 | 184.9082 | 192.9412 | 197.1996 | 279.4356 | 280.3082 | 288.7612 | 168.5792 | 178.7292 | 189.0474 |
| 909 | pos_294 | Argininosuccinic acid | 17.73902 | 18.90512 | 20.43443 | 7.220531 | 7.225564 | 7.716989 | 1.573111 | 1.721087 | 1.828089 | 13.05032 | 14.16202 | 14.7443 |
| 910 | pos_295 | Deoxyguanosine | 15.45005 | 15.98728 | 16.84165 | 250.5718 | 253.1276 | 262.2066 | 210.656 | 222.6607 | 232.8075 | 261.6212 | 271.1991 | 292.2056 |
| 911 | pos_296 | 5-Aminopentanal | 88.49119 | 95.40046 | 101.847 | 32.88779 | 32.92029 | 34.81831 | 10.32279 | 10.5427 | 11.12907 | 76.62641 | 78.99102 | 85.80072 |
| 912 | pos_297 | 5'-(dimethylsulfonio)-5'-deoxyadenosine | 14897.39 | 15426.97 | 16668.2 | 37229.86 | 38936.93 | 39545.59 | 16871.17 | 17668.25 | 19141.8 | 39900.98 | 44020.81 | 46562.21 |
| 913 | pos_298 | (2S,3S)-2,3-Dihydro-2,3-dihydroxybenzoate | 7848.144 | 8085.137 | 8543.707 | 400.524 | 430.7191 | 447.5108 | 5170.928 | 5347.977 | 5814.173 | 231.4997 | 241.7892 | 251.1095 |
| 914 | pos_299 | Zingerone | 10250.01 | 10778.48 | 11439.52 | 7171.834 | 7326.311 | 7967.337 | 11948.44 | 11962.32 | 12335.44 | 11354.22 | 11486.84 | 12598.02 |
| 915 | pos_300 | (R)-Mevalonic acid | 53.07443 | 53.40272 | 56.53145 | 54.38097 | 56.30682 | 56.34232 | 49.38306 | 49.84495 | 51.39586 | 49.5427 | 51.62895 | 55.40874 |
| 916 | pos_301 | S-Adenosylmethioninamine | 11.99425 | 12.14904 | 12.20431 | 5.728135 | 5.811841 | 5.823345 | 1994.6 | 2040.376 | 2075.626 | 6.06162 | 6.327753 | 6.646049 |
| 917 | pos_302 | S-Ribosyl-L-homocysteine | 243.054 | 269.3743 | 284.5707 | 77.83909 | 84.33831 | 88.97394 | 149.5026 | 152.6034 | 162.0991 | 64.62372 | 69.78881 | 71.0268 |
| 918 | pos_303 | N2-Succinyl-L-glutamic acid 5-semialdehyde | 1.135463 | 1.15699 | 1.214848 | 71.04061 | 73.48284 | 74.72972 | 78.67786 | 80.66349 | 82.50847 | 84.44699 | 92.07351 | 98.73163 |
| 919 | pos_304 | 2-Methylacetoacetyl-CoA | 449.467 | 454.648 | 455.9539 | 0 | 0 | 0 | 0 | 0 | 0 | 0 | 0 | 0 |
| 920 | pos_305 | N-Acetyl-glucosamine 1-phosphate | 35.43409 | 38.72199 | 41.80643 | 176.3238 | 176.5713 | 182.9982 | 146.5977 | 150.5265 | 154.3209 | 158.112 | 160.8851 | 168.4317 |
| 921 | pos_306 | cis-2-Methylaconitate | 3629.27 | 3750.521 | 3861.257 | 18.29759 | 19.5592 | 21.29854 | 14.1361 | 15.06648 | 16.09848 | 11.62993 | 11.7215 | 12.13326 |
| 922 | pos_307 | PS(16:0/18:1(11Z)) | 8648.265 | 9487.402 | 10222.55 | 8112.888 | 8745.766 | 8862.288 | 8834.467 | 8904.974 | 9781.45 | 8038.535 | 8565.657 | 9191.88 |
| 923 | pos_308 | cellobiose-6-phosphate | 55.38782 | 56.76008 | 58.67869 | 143.1554 | 157.6037 | 159.2362 | 58.59177 | 63.54514 | 65.63349 | 138.7635 | 143.1985 | 146.7724 |
| 924 | pos_309 | Tetradecenoylcarnitine | 342.3698 | 364.5316 | 386.7629 | 366.9723 | 404.0433 | 411.7647 | 392.4008 | 416.9905 | 419.1373 | 366.3199 | 377.0362 | 389.3107 |
| 925 | pos_310 | Sucrose | 1026.282 | 1029.599 | 1109.747 | 939.6027 | 1012.879 | 1060.269 | 886.9417 | 949.192 | 1026.461 | 979.2752 | 1037.859 | 1082.475 |
| 926 | pos_311 | UDP-N-acetylmuramoyl-L-alanyl-D-glutamyl-meso-2,6-diaminoheptanedioate | 106.1807 | 112.3242 | 116.771 | 9.338717 | 9.77987 | 10.3757 | 6.077288 | 6.331586 | 6.427756 | 6.587292 | 6.631589 | 6.683093 |
| 927 | pos_312 | N-Acetyl-L-glutamate 5-semialdehyde | 46.00936 | 46.47449 | 49.414 | 96.15633 | 106.2476 | 110.0086 | 95.10399 | 99.85751 | 109.1851 | 87.91467 | 92.53732 | 99.68504 |
| 928 | pos_313 | Acetamidopropanal | 31.99653 | 32.28917 | 35.03362 | 127.916 | 136.2018 | 146.9144 | 52.03603 | 53.76635 | 53.88457 | 123.3685 | 134.2522 | 142.143 |
| 929 | pos_314 | isopropyl beta-D-galactopyranoside | 8.767036 | 9.252033 | 9.777013 | 18.95278 | 20.17636 | 21.41041 | 10.76502 | 11.54056 | 12.00468 | 13.82376 | 13.90742 | 15.00454 |
| 930 | pos_315 | Peonidin | 30.25875 | 31.18479 | 32.19809 | 204.7005 | 212.5324 | 222.752 | 202.2363 | 202.7652 | 222.6162 | 251.1409 | 258.1831 | 262.4304 |

Continued Table S2

| 931 | pos_316 | lipol-AMP | 57.79031 | 62.203 | 63.79197 | 366.0129 | 376.4537 | 384.3474 | 147.3289 | 161.8249 | 162.7762 | 299.1833 | 324.0556 | 346.0408 |
| --- | --- | --- | --- | --- | --- | --- | --- | --- | --- | --- | --- | --- | --- | --- |
| 932 | pos_317 | 3-Hydroxy-5-oxohexanoyl-CoA | 19.82228 | 21.68711 | 23.22256 | 160.7243 | 166.9347 | 167.073 | 58.43836 | 60.87673 | 63.90867 | 142.084 | 157.523 | 167.2852 |
| 933 | pos_318 | 4beta-methylzymosterol-4alpha-carboxylic acid | 0 | 0 | 0 | 122.3133 | 128.5441 | 136.6569 | 0.660314 | 0.680409 | 0.718637 | 1.049164 | 1.099711 | 1.102326 |
| 934 | pos_319 | Astringin | 0 | 0 | 0 | 0 | 0 | 0 | 235.7832 | 243.4658 | 257.8858 | 0 | 0 | 0 |
| 935 | pos_320 | NADH | 90.49044 | 95.86417 | 104.3816 | 41.10851 | 45.17072 | 49.14584 | 40.89835 | 43.85103 | 46.1804 | 37.34655 | 41.07944 | 42.20699 |
| 936 | pos_321 | penicillin G | 77.07194 | 82.62904 | 90.88622 | 5.853557 | 5.919494 | 6.362325 | 2.972065 | 3.169656 | 3.308645 | 2.690219 | 2.714948 | 2.740285 |
| 937 | pos_322 | Dyspropterin | 3917.27 | 4245.12 | 4428.884 | 1762.041 | 1956.065 | 2002.142 | 2512.667 | 2676.472 | 2697.955 | 1399.78 | 1532.826 | 1596.835 |
| 938 | pos_323 | (2,3-Dihydroxybenzoyl)adenylate | 4.88864 | 5.169353 | 5.54842 | 195.0815 | 196.5684 | 200.1564 | 166.6807 | 182.9023 | 195.3593 | 181.6551 | 181.8742 | 198.3392 |
| 939 | pos_324 | PC(16:0/0:0) | 401.6704 | 416.0628 | 446.0272 | 259.7374 | 287.2596 | 300.9402 | 338.1826 | 373.2295 | 395.2545 | 171.005 | 185.0106 | 203.3853 |
| 940 | pos_325 | PS(16:1(9Z)/16:1(9Z)) | 380.3446 | 392.1261 | 428.8218 | 544.181 | 575.173 | 580.1323 | 155.1184 | 159.3567 | 162.043 | 482.4852 | 500.0068 | 513.7295 |
| 941 | pos_326 | D-Galactosamine 6-phosphate | 38.36066 | 39.1526 | 42.14071 | 34.29759 | 37.48778 | 40.87178 | 49.68126 | 52.53561 | 53.30793 | 42.45739 | 44.79728 | 48.79267 |
| 942 | pos_327 | Gamma-Glutamylcysteine | 0 | 0 | 0 | 68.97318 | 75.09869 | 75.79314 | 36.36516 | 38.71601 | 41.59308 | 66.25198 | 72.60632 | 77.42845 |
| 943 | pos_328 | fructoselysine-6-phosphate | 123.9553 | 125.3975 | 130.5513 | 7.575981 | 7.869254 | 8.134983 | 12.80031 | 14.03393 | 14.80625 | 5.701042 | 6.097144 | 6.262403 |
| 944 | pos_329 | Acetyl adenylate | 598.2373 | 616.1773 | 642.0147 | 63.67907 | 66.6489 | 67.4764 | 45.7372 | 49.7427 | 49.81166 | 70.13481 | 73.57372 | 80.67111 |
| 945 | pos_330 | 2',3'-Cyclic UMP | 411.6288 | 448.6077 | 489.4668 | 686.8088 | 705.1902 | 744.7104 | 209.5837 | 222.4257 | 238.9014 | 626.1071 | 648.5197 | 705.282 |
| 946 | pos_331 | N5-methyl-L-glutamine | 230.1256 | 243.2038 | 262.5512 | 254.0492 | 265.7021 | 291.1796 | 297.5972 | 299.6707 | 320.1648 | 263.0472 | 271.5957 | 277.0483 |
| 947 | pos_332 | 4-Hydroxy-2-oxopentanoate | 210.2632 | 212.2726 | 214.56 | 508.8849 | 531.6001 | 581.8045 | 233.8709 | 246.3284 | 255.3219 | 529.1673 | 534.5271 | 534.7978 |
| 948 | pos_333 | Glucose 1-phosphate | 442.8518 | 460.628 | 506.065 | 756.3525 | 760.6329 | 784.2966 | 517.7691 | 531.7518 | 575.281 | 780.0333 | 809.397 | 854.6785 |
| 949 | pos_334 | S(8)-aminomethyldihydrolipoamide | 835.463 | 858.5199 | 942.7156 | 1057.659 | 1085.08 | 1095.96 | 988.0228 | 1074.965 | 1096.288 | 952.1082 | 999.5762 | 1026.639 |
| 950 | pos_335 | L-rhamnitol | 0 | 0 | 0 | 0 | 0 | 0 | 235.7383 | 237.8775 | 250.9123 | 0 | 0 | 0 |
| 951 | pos_336 | globomycin | 3.711308 | 3.99135 | 4.203632 | 24.41638 | 27.01605 | 29.37111 | 52.23261 | 52.96123 | 58.10301 | 11.58255 | 12.75513 | 13.6475 |
| 952 | pos_337 | PA(16:1(9Z)/0:0) | 2257.825 | 2344.659 | 2399.321 | 2296.697 | 2531.726 | 2682.758 | 2328.263 | 2418.779 | 2510.879 | 2230.283 | 2446.869 | 2512.03 |
| 953 | pos_338 | cumene hydroperoxide | 30.55554 | 31.0593 | 31.79779 | 31.50382 | 31.62387 | 33.98929 | 33.95995 | 34.9452 | 36.81136 | 44.44292 | 46.18959 | 50.65079 |
| 954 | pos_339 | Porphobilinogen | 205.2019 | 225.8461 | 240.6631 | 62.3941 | 62.76731 | 63.56962 | 141.2345 | 143.641 | 144.0224 | 23.8643 | 24.91798 | 25.3911 |
| 955 | pos_340 | dTDP-D-fucosamine | 15.3717 | 15.85386 | 16.16362 | 19.83905 | 21.26674 | 21.97003 | 13.39053 | 14.86524 | 15.90344 | 36.99675 | 39.55194 | 39.93458 |

Continued Table S2

| 956 | pos_341 | Orotidylic acid | 0 | 0 | 0 | 0 | 0 | 0 | 67.54941 | 70.49335 | 74.72953 | 0 | 0 | 0 |
| --- | --- | --- | --- | --- | --- | --- | --- | --- | --- | --- | --- | --- | --- | --- |
| 957 | pos_342 | 4,5-Dihydro-4-hydroxy-5-S-glutathionyl-benzo[a]pyrene | 179.2924 | 196.6933 | 201.6312 | 0 | 0 | 0 | 0 | 0 | 0 | 0 | 0 | 0 |
| 958 | pos_343 | Sucrose-6-phosphate | 52.54348 | 57.68774 | 62.8895 | 371.7031 | 393.6977 | 411.6556 | 258.7547 | 258.9396 | 270.9366 | 256.4833 | 280.3087 | 301.0789 |
| 959 | pos_344 | 3-Carboxy-2,3,4,9-tetrahydro-1H-pyrido[3,4-b]indole-1-propanoic acid | 101.2748 | 111.4317 | 121.6025 | 133.0325 | 146.7291 | 156.0869 | 240.8435 | 245.0201 | 249.4155 | 185.6961 | 187.3553 | 192.6766 |
| 960 | pos_345 | D,L-cyclohexanephosphinothricin | 234.2828 | 255.7383 | 262.6625 | 141.0541 | 149.706 | 162.0923 | 107.4459 | 119.0969 | 119.2589 | 142.8977 | 143.4143 | 143.918 |
| 961 | pos_346 | CDPglucose | 330.133 | 339.2679 | 355.9945 | 35.37865 | 39.21218 | 39.50117 | 14.78838 | 14.97598 | 16.17135 | 41.60676 | 42.87325 | 46.93493 |
| 962 | pos_347 | Tiglyl-CoA | 2.017554 | 2.149805 | 2.184961 | 28.44652 | 29.6166 | 31.9485 | 263.7443 | 271.7801 | 292.0273 | 21.45036 | 23.61216 | 25.55522 |
| 963 | pos_348 | 2-(S-Glutathionyl)acetyl chloride | 267.3855 | 269.2048 | 278.812 | 101.4594 | 103.8984 | 112.5379 | 194.566 | 211.4156 | 226.9546 | 59.45646 | 59.90537 | 62.14562 |
| 964 | pos_349 | L-Palmitoylcarnitine | 3317.169 | 3460.455 | 3751.571 | 3378.201 | 3589.584 | 3897.07 | 3066.853 | 3297.365 | 3399.556 | 3345.132 | 3517.173 | 3601.609 |
| 965 | pos_350 | 3-Hydroxypropionyl-CoA | 608.2835 | 642.2616 | 704.945 | 491.9689 | 522.2775 | 556.7388 | 388.7153 | 396.659 | 425.1663 | 494.3181 | 500.7205 | 508.4953 |
| 966 | pos_351 | UDP-N-acetyl-3-(1-carboxyvinyl)-D-glucosamine | 23.03508 | 25.03129 | 26.04453 | 21.99017 | 23.04997 | 24.63127 | 47.06653 | 49.10092 | 49.67111 | 30.05357 | 31.0609 | 33.01724 |
| 967 | pos_352 | dIMP | 413.1311 | 451.1834 | 454.3331 | 3.843605 | 3.850241 | 3.891897 | 57.33507 | 60.81642 | 66.25691 | 4.136791 | 4.579975 | 4.695402 |
| 968 | pos_353 | xanthosine | 295.5785 | 328.1424 | 344.4638 | 404.2595 | 419.0931 | 460.8953 | 370.7977 | 384.1507 | 408.5947 | 490.5383 | 529.8071 | 558.894 |
| 969 | pos_354 | ergosteryl 3-beta-D-glucoside | 323.3485 | 335.6836 | 343.9344 | 349.5647 | 378.3901 | 404.4404 | 331.271 | 342.9751 | 373.8601 | 345.1596 | 345.9366 | 379.0242 |
| 970 | pos_355 | cis-3-(3-Carboxyethenyl)-3,5-cyclohexadiene-1,2-diol | 108.0951 | 117.7845 | 125.8166 | 96.36984 | 98.90717 | 105.3905 | 105.1735 | 106.3355 | 110.3334 | 99.57316 | 103.4214 | 113.451 |
| 971 | pos_356 | PE(18:1(11Z)/16:1(9Z)) | 10068.79 | 10453.51 | 11277.19 | 6103.868 | 6118.956 | 6316.497 | 8170.853 | 8322.52 | 8447.96 | 4624.411 | 4792.277 | 4896.56 |
| 972 | pos_357 | PE(19:0/17:0) | 50344.77 | 55207.24 | 58121.92 | 43238.82 | 43642.38 | 47398.81 | 44764.09 | 47182.9 | 51550.81 | 42640.08 | 46912.97 | 49617.62 |
| 973 | pos_358 | octyl beta-1,6-D-galactofuranosyl-alpha-D-glucopyranoside | 0 | 0 | 0 | 1.485848 | 1.628114 | 1.746871 | 124.3705 | 130.6575 | 136.1178 | 0 | 0 | 0 |
| 974 | pos_359 | 5-Ethyl-4-methyloxazole | 4389.329 | 4469.175 | 4674.759 | 3327.637 | 3459.976 | 3532.551 | 3313.623 | 3339.692 | 3546.858 | 3201.679 | 3444.826 | 3756.779 |
| 975 | pos_360 | tris-acetate | 0 | 0 | 0 | 9.683903 | 10.19258 | 10.20495 | 0 | 0 | 0 | 20.30061 | 21.54794 | 22.437 |
| 976 | pos_361 | L-3-Oxoalanine | 0.005868 | 0.006168 | 0.006439 | 46.49066 | 51.37589 | 52.84604 | 0.009544 | 0.010326 | 0.011183 | 132.7044 | 144.1708 | 149.4083 |

Continued Table S2

| 977 | pos_362 | PC(18:1(9Z)/0:0) | 132.096 | 134.1089 | 138.7282 | 79.94146 | 88.27935 | 90.56399 | 136.2481 | 144.906 | 146.876 | 52.80134 | 56.39411 | 60.341 |
| --- | --- | --- | --- | --- | --- | --- | --- | --- | --- | --- | --- | --- | --- | --- |
| 978 | pos_363 | Hexanediol | 2.447919 | 2.596832 | 2.779995 | 784.9477 | 809.5642 | 889.9768 | 632.9116 | 651.7277 | 712.4645 | 1517.562 | 1665.705 | 1810.296 |
| 979 | pos_364 | Gallic acid | 4902.758 | 5289.527 | 5444.934 | 19.91755 | 21.3925 | 22.94268 | 31.33887 | 31.59205 | 34.12128 | 25.16179 | 25.25425 | 25.28174 |
| 980 | pos_365 | L-seryl-AMP | 67.36518 | 69.72021 | 74.49165 | 90.83937 | 96.95518 | 102.2704 | 203.8675 | 219.8858 | 236.1209 | 92.41827 | 96.56022 | 104.4289 |
| 981 | pos_366 | 7,8-Dihydroneopterin | 53.84582 | 57.82835 | 58.70738 | 65.44312 | 71.06282 | 77.56326 | 76.48725 | 76.74189 | 77.70829 | 69.12144 | 76.30439 | 83.39119 |
| 982 | pos_367 | Heptyl acetate | 1134.123 | 1196.368 | 1284.849 | 1580.186 | 1605.23 | 1611.033 | 1213.329 | 1238.811 | 1320.333 | 1368.209 | 1496.61 | 1613.076 |
| 983 | pos_368 | 5-Amino-6-ribitylamino uracil | 25.3775 | 27.26332 | 29.53155 | 27.09068 | 30.01274 | 30.64186 | 17.11283 | 18.97077 | 19.10836 | 24.68231 | 24.97842 | 26.78112 |
| 984 | pos_369 | 4-Allyl-2-methoxyphenol | 1.498083 | 1.624068 | 1.658473 | 3.392921 | 3.546818 | 3.642767 | 1.530775 | 1.654462 | 1.749612 | 2.110412 | 2.323062 | 2.551695 |
| 985 | pos_370 | Phosphoribosyl-AMP | 224.2218 | 236.625 | 246.222 | 74.18657 | 80.62569 | 83.08389 | 190.1833 | 199.6428 | 199.9296 | 67.51835 | 73.45341 | 75.87345 |
| 986 | pos_371 | 3-mercaptohexyl acetate | 28.2221 | 29.53575 | 31.03285 | 26.43284 | 26.96839 | 27.01102 | 21.2644 | 23.61678 | 25.62779 | 23.75405 | 25.9753 | 28.53584 |
| 987 | pos_372 | 3-Hydroxy-7,8-dihydro-beta-ionol | 21.81089 | 23.35838 | 24.19465 | 98.60869 | 108.2127 | 113.2934 | 46.10509 | 46.29496 | 46.51756 | 34.43907 | 38.24475 | 41.73812 |
| 988 | pos_373 | Gamma-Aminobutyric acid | 0 | 0 | 0 | 76.21744 | 76.79862 | 82.2911 | 19.63209 | 21.48759 | 23.228 | 55.18857 | 59.93669 | 64.79395 |
| 989 | pos_374 | vitispirane | 115.2555 | 124.5306 | 136.002 | 118.1492 | 122.777 | 134.4038 | 124.6855 | 134.4895 | 140.5292 | 121.8876 | 122.5504 | 123.0022 |
| 990 | pos_375 | (1R)-Hydroxy-(2R)-glutathionyl-1,2-dihydronaphthalene | 134.5636 | 142.0739 | 145.2118 | 0 | 0 | 0 | 0.104718 | 0.1152 | 0.115913 | 0.007465 | 0.008088 | 0.008619 |
| 991 | pos_376 | 1-Methyladenosine | 101.1788 | 102.7814 | 103.9118 | 80.84808 | 89.76659 | 91.95306 | 92.23887 | 93.18875 | 94.499 | 84.08604 | 86.01346 | 89.82646 |
| 992 | pos_377 | serinamide | 61.8968 | 67.64376 | 70.83025 | 7.505502 | 7.535545 | 8.273925 | 5.886715 | 6.272914 | 6.489222 | 5.499298 | 6.08378 | 6.186882 |
| 993 | pos_378 | 2-Octaprenyl-3-methyl-6-methoxy-1,4-benzoquinol | 193.9283 | 199.1379 | 200.1279 | 261.1707 | 288.4659 | 304.4218 | 674.2135 | 693.4503 | 720.7023 | 194.693 | 205.9098 | 211.2367 |
| 994 | pos_379 | Biotin | 592.3742 | 599.0318 | 631.3147 | 6712.87 | 7146.344 | 7207.818 | 7333.263 | 7337.033 | 8019.036 | 7387.227 | 7436.034 | 7524.434 |
| 995 | pos_380 | PE(18:0/18:1(9Z)) | 36055.96 | 36627.4 | 37689.74 | 27222.47 | 28535.1 | 30351.43 | 31658.83 | 34373.43 | 36919.75 | 14912.59 | 16065.06 | 16527.97 |
| 996 | pos_381 | PS(19:0/16:0) | 11259.16 | 11627.81 | 12091.46 | 6442.067 | 6753.515 | 7279.468 | 10193.62 | 10351.08 | 11089.53 | 4483.793 | 4527.15 | 4592.417 |
| 997 | pos_382 | PE(18:1(11Z)/16:0) | 185.4525 | 198.3938 | 202.5643 | 162.5851 | 173.2038 | 180.2517 | 167.4483 | 179.9226 | 185.5916 | 120.995 | 121.8053 | 123.3326 |
| 998 | pos_383 | (6R)-6-(l-erythro-1,2-dihydroxypropyl)-5,6,7,8-tetrahydro-4a-hydroxypterin | 50.1992 | 51.65178 | 55.3383 | 32.64648 | 34.24919 | 36.27075 | 36.52166 | 40.00276 | 42.63162 | 36.9499 | 40.36985 | 42.15761 |
| 999 | pos_384 | Adenosine | 9681.436 | 10177.07 | 10682.69 | 457.0058 | 477.4145 | 513.3832 | 183.3538 | 194.8423 | 194.9023 | 970.0627 | 1074.617 | 1141.825 |

Continued Table S2

| 1000 | pos_385 | Tyrosol | 23.67904 | 26.06231 | 27.97193 | 14.6552 | 15.43329 | 16.97567 | 16.55727 | 16.99569 | 18.39817 | 9.211429 | 10.04642 | 10.06334 |
| --- | --- | --- | --- | --- | --- | --- | --- | --- | --- | --- | --- | --- | --- | --- |
| 1001 | pos_386 | formycin B | 152.7574 | 156.9078 | 161.5639 | 116.7015 | 129.4074 | 135.839 | 105.5128 | 114.4 | 124.3764 | 75.21778 | 81.14482 | 88.34168 |
| 1002 | pos_387 | N-Succinyl-2-amino-6-ketopimelate | 208.629 | 228.0651 | 245.0864 | 250.6268 | 259.6019 | 268.8731 | 259.0004 | 268.2117 | 283.6897 | 312.3327 | 345.2132 | 350.8691 |
| 1003 | pos_388 | 3-Hydroxy-2-methyl-4-pyrone | 3197.075 | 3260.012 | 3301.322 | 2821.184 | 3055.731 | 3167.142 | 2935.483 | 3188.038 | 3301.813 | 3023.731 | 3056.687 | 3143.67 |
| 1004 | pos_389 | 2-(alpha-D-Mannosyl)-3-phosphoglycerate | 51.39024 | 54.69866 | 55.51829 | 87.98281 | 88.18199 | 89.34741 | 17.21578 | 18.21305 | 19.77752 | 43.74376 | 44.34474 | 47.17292 |
| 1005 | pos_390 | (R)-Pantothenic acid | 161.0166 | 177.9686 | 188.4036 | 12044.74 | 12315.89 | 12912.48 | 3203.158 | 3439.691 | 3652.072 | 10350.69 | 11399.68 | 12535.4 |
| 1006 | pos_391 | 2-Deoxyglucose | 473.2064 | 480.8622 | 488.902 | 33.47555 | 34.41754 | 36.80986 | 42.86207 | 47.58118 | 48.9446 | 32.93394 | 34.17104 | 34.69783 |
| 1007 | pos_392 | PS(17:0/18:1(11Z)) | 35339.54 | 35841.25 | 38364 | 26614.6 | 29468.64 | 30604.51 | 30876.11 | 34175.49 | 34680.86 | 24329.17 | 25018.64 | 26038.9 |
| 1008 | pos_393 | Citrulline | 313.3331 | 345.0496 | 350.966 | 181.5527 | 189.3976 | 204.6821 | 188.1982 | 189.2129 | 200.9301 | 189.7509 | 197.8752 | 211.3435 |
| 1009 | pos_394 | P1-uridyl-P2-methyl diphosphate | 110.8987 | 118.0555 | 127.7651 | 0 | 0 | 0 | 0 | 0 | 0 | 0 | 0 | 0 |
| 1010 | pos_395 | Tiglylcarnitine | 54.21189 | 54.6895 | 58.57651 | 86.5254 | 89.67743 | 97.70388 | 585.3628 | 645.508 | 682.0342 | 100.6967 | 104.136 | 106.2238 |
| 1011 | pos_396 | zymosteryl oleate | 1743.606 | 1920.69 | 2032.043 | 1726.332 | 1832.846 | 2010.854 | 1913.339 | 2055.823 | 2226.356 | 1793.262 | 1886.861 | 2000.403 |
| 1012 | pos_397 | 15(S)-HETE | 23.6658 | 23.82708 | 26.16886 | 142.5629 | 145.765 | 149.8921 | 47.67104 | 52.16487 | 53.59312 | 33.90529 | 34.5132 | 37.95563 |
| 1013 | pos_398 | N-benzylbenzamidine | 2.471208 | 2.683625 | 2.839869 | 113.4536 | 124.8443 | 127.4138 | 134.7817 | 140.4807 | 144.1155 | 93.50292 | 94.06679 | 102.3963 |
| 1014 | pos_399 | carboxin | 0 | 0 | 0 | 56.68935 | 62.13652 | 62.64434 | 32.23505 | 34.98453 | 37.57788 | 38.22068 | 39.35531 | 42.30959 |
| 1015 | pos_400 | 5-Phosphoribosylamine | 172.8159 | 188.8324 | 196.6414 | 0 | 0 | 0 | 0 | 0 | 0 | 0 | 0 | 0 |
| 1016 | pos_401 | 1-monohexadecanoylglycerol | 3764.137 | 3798.212 | 4112.193 | 14052.75 | 15211.24 | 15545.93 | 4845.753 | 5369.719 | 5828.277 | 11148.22 | 11527.13 | 12127.99 |
| 1017 | pos_402 | D-Galactosamine | 61.42065 | 65.38409 | 70.51019 | 83.06703 | 87.56412 | 93.74412 | 91.35943 | 93.28383 | 96.83261 | 83.2405 | 85.1184 | 93.03601 |
| 1018 | pos_403 | PhosphoribosylformiminoAICAR-phosphate | 163.785 | 163.9752 | 167.0324 | 0 | 0 | 0 | 7.856904 | 7.89497 | 8.552833 | 6.406233 | 7.077903 | 7.464953 |
| 1019 | pos_404 | Gluconolactone | 53.77247 | 58.08493 | 63.69399 | 12.78483 | 12.80106 | 14.03798 | 12.79081 | 13.0181 | 13.27304 | 10.42912 | 11.3355 | 12.41248 |
| 1020 | pos_405 | o-nitrophenol beta-thiogalactoside | 35.97354 | 39.30617 | 40.04224 | 69.57383 | 76.30309 | 81.84509 | 61.67092 | 61.78412 | 64.16091 | 105.1784 | 111.0559 | 114.6307 |
| 1021 | pos_406 | Hotrienol | 28.08358 | 29.67123 | 32.02542 | 29.36639 | 30.33017 | 32.36634 | 31.29561 | 32.04034 | 34.55568 | 26.1917 | 29.04794 | 30.69748 |
| 1022 | pos_407 | 9-[6(RS)-C-carboxamido-5,6,7-trideoxy-beta-D-ribo-octofuranosyl]-9H-purin-6-amine | 475.5677 | 527.1462 | 557.6729 | 547.0744 | 568.4556 | 612.554 | 1277.02 | 1384.534 | 1443.131 | 590.1936 | 612.2995 | 654.0545 |
| 1023 | pos_408 | Ethyl 4-hydroxybutanoate | 458.1409 | 492.2431 | 509.7182 | 490.2254 | 495.3844 | 517.4298 | 470.9142 | 504.0505 | 539.9676 | 475.8972 | 488.7259 | 536.7347 |
| 1024 | pos_409 | actinonin | 13.08419 | 14.1599 | 14.48238 | 17.50781 | 17.79732 | 18.81097 | 365.2747 | 404.1239 | 430.7327 | 19.34904 | 19.5303 | 21.46607 |

Continued Table S2

| 1025 | pos_410 | methyl-1,4-benzoquinone | 467.8307 | 513.1048 | 556.7351 | 428.1759 | 469.9434 | 516.0196 | 454.4913 | 499.1601 | 515.1498 | 444.6366 | 465.4986 | 510.7994 |
| --- | --- | --- | --- | --- | --- | --- | --- | --- | --- | --- | --- | --- | --- | --- |
| 1026 | pos_411 | ADP-D-ribose 1''-phosphate | 379.1333 | 407.0265 | 446.6061 | 20.34438 | 22.11783 | 24.2805 | 0 | 0 | 0 | 27.82644 | 28.45309 | 30.67843 |
| 1027 | pos_412 | 2-phenylethanol | 39.5733 | 41.96771 | 45.20914 | 1.375801 | 1.491629 | 1.624966 | 3.97121 | 4.161214 | 4.224611 | 1.855692 | 1.97694 | 2.173003 |
| 1028 | pos_413 | N1-Acetylspermine | 3911.762 | 3914.926 | 4251.192 | 3811.216 | 4088.459 | 4416.518 | 2948.706 | 3084.604 | 3308.258 | 3577.471 | 3952.809 | 4191.38 |
| 1029 | pos_414 | Amyl propanoate | 153.2092 | 154.241 | 159.9228 | 150.5992 | 161.5149 | 176.789 | 149.1183 | 150.3876 | 163.3256 | 143.1816 | 157.7057 | 168.0483 |
| 1030 | pos_415 | PE(19:0/16:1(9Z)) | 103823.5 | 107426.8 | 109810.8 | 89031.15 | 96557.5 | 100646 | 97732.6 | 99851.18 | 106388.8 | 56289.82 | 56885.65 | 60633.61 |
| 1031 | pos_416 | N7-Pan | 59.84181 | 59.91349 | 65.38822 | 51.83981 | 57.32145 | 58.56762 | 54.19052 | 55.56949 | 58.92677 | 48.96798 | 51.39765 | 56.21658 |
| 1032 | pos_417 | 4-acetamidobutanal | 8.879907 | 9.364431 | 9.855288 | 0 | 0 | 0 | 9.74955 | 10.41241 | 10.76905 | 0 | 0 | 0 |
| 1033 | pos_418 | 1-Aminocyclopropane-1-carboxylic acid | 567.8256 | 581.0093 | 594.1346 | 1643.592 | 1776.385 | 1861.316 | 272.5911 | 277.6184 | 301.662 | 1892.797 | 1960.863 | 2061.879 |
| 1034 | pos_419 | Cytidine monophosphate | 117.2285 | 122.4115 | 123.8885 | 15.55413 | 16.28468 | 17.57797 | 32.86299 | 34.81493 | 37.76282 | 16.55101 | 17.24501 | 17.29236 |
| 1035 | pos_420 | Hexa-2,4-dienol | 58.32608 | 62.52568 | 67.55385 | 39.66825 | 41.20802 | 42.42603 | 39.72622 | 43.95115 | 48.28717 | 41.37492 | 45.54168 | 45.58741 |
| 1036 | pos_421 | Quercetin 3-O-glucuronide | 151.7088 | 159.0587 | 163.4428 | 13.95162 | 15.16963 | 15.74947 | 27.26146 | 29.30162 | 30.36544 | 18.64891 | 20.24718 | 20.41027 |
| 1037 | pos_422 | Hexyl acetate | 1149.982 | 1215.312 | 1278.786 | 1228.835 | 1242.66 | 1320.394 | 1157.654 | 1233.12 | 1274.731 | 1206.355 | 1227.253 | 1252.774 |
| 1038 | pos_423 | Itaconyl-CoA | 1343.106 | 1450.755 | 1457.282 | 291.9971 | 319.0389 | 331.4071 | 293.3471 | 302.2614 | 312.5757 | 324.1514 | 347.2463 | 374.4953 |
| 1039 | pos_424 | Arbutin | 42.6441 | 45.10663 | 46.17577 | 174.8149 | 178.4553 | 187.7805 | 3.557417 | 3.572415 | 3.576834 | 338.5354 | 344.9697 | 359.9917 |
| 1040 | pos_425 | S-adenosyl-1,8-diamino-3-thiooctane | 77.26565 | 79.67732 | 84.46225 | 0 | 0 | 0 | 0 | 0 | 0 | 0 | 0 | 0 |
| 1041 | pos_426 | 2-Isopropyl-3-oxosuccinate | 17844.82 | 19450.05 | 21106.47 | 419.5401 | 427.463 | 428.3784 | 586.8302 | 614.7844 | 647.2097 | 315.4591 | 336.2713 | 342.4969 |
| 1042 | pos_427 | S-Formylglutathione | 749.8876 | 762.3378 | 814.3257 | 358.749 | 361.0962 | 377.2038 | 280.9144 | 285.5513 | 298.9455 | 392.6963 | 416.953 | 432.95 |
| 1043 | pos_428 | coformycin | 617.7021 | 645.443 | 677.3273 | 108.1146 | 113.3561 | 122.0313 | 115.3864 | 117.0505 | 124.8705 | 59.31414 | 60.89804 | 62.73717 |
| 1044 | pos_429 | Glycineamideribotide | 5.005029 | 5.344996 | 5.447658 | 120.2472 | 131.695 | 140.077 | 160.8938 | 177.8088 | 180.5367 | 112.5011 | 113.5447 | 114.1542 |
| 1045 | pos_430 | (R)-Pantoate | 0 | 0 | 0 | 88.37246 | 93.96236 | 95.04179 | 2.760264 | 2.939898 | 2.959435 | 212.9956 | 216.2807 | 227.9338 |
| 1046 | pos_431 | Furylacetone | 23.40819 | 24.02507 | 24.8246 | 11.36547 | 11.74379 | 12.78479 | 16.34487 | 16.56515 | 18.05292 | 9.684122 | 9.939427 | 10.67363 |
| 1047 | pos_432 | S(8)-succinyldihydrolipoamide | 3014.531 | 3051.994 | 3179.616 | 3552.717 | 3758.466 | 3889.025 | 3294.376 | 3390.804 | 3628.405 | 3634.078 | 3840.176 | 4164.638 |
| 1048 | pos_433 | Phylloquinone | 10828.51 | 11898.68 | 12032.63 | 11685.12 | 12451.67 | 12667.38 | 11355.35 | 11958.58 | 12622.15 | 11565.07 | 11874.14 | 12258.03 |
| 1049 | pos_434 | 2-methyltetrahydrothiophen-3-one | 1734.027 | 1907.842 | 2082.459 | 1736.472 | 1777.272 | 1909.237 | 1706.025 | 1722.702 | 1884.42 | 1597.36 | 1740.795 | 1834.755 |
| 1050 | pos_435 | Chitobiose | 524.7551 | 528.4936 | 570.3698 | 661.2858 | 710.3582 | 767.8123 | 543.1548 | 589.1824 | 616.3299 | 601.0667 | 639.2283 | 673.3954 |

Continued Table S2

| 1051 | pos_436 | DG(18:0/18:1(9Z)/0:0) | 824.5216 | 864.2382 | 940.011 | 756.1867 | 794.0813 | 814.8112 | 701.9394 | 772.5907 | 834.19 | 749.3872 | 778.4433 | 817.8527 |
| --- | --- | --- | --- | --- | --- | --- | --- | --- | --- | --- | --- | --- | --- | --- |
| 1052 | pos_437 | zymosterol | 265.702 | 270.1319 | 294.6916 | 275.6683 | 277.3918 | 290.9593 | 532.1939 | 537.4715 | 552.5822 | 414.6289 | 431.0617 | 459.4947 |
| 1053 | pos_438 | 2-Dehydro-3-deoxy-D-arabino-heptonate 7-phosphate | 34.87427 | 35.75309 | 37.2527 | 35.60625 | 35.6828 | 36.37111 | 42.28986 | 42.55769 | 44.2685 | 20.719 | 22.13415 | 23.38487 |
| 1054 | pos_439 | Malvidin | 452.7285 | 473.4818 | 506.6172 | 214.2529 | 219.5536 | 229.8624 | 217.5225 | 223.2647 | 237.253 | 209.1919 | 219.7268 | 234.984 |
| 1055 | pos_440 | Niacinamide | 1834.095 | 1949.178 | 1979.198 | 512.1118 | 543.896 | 576.4132 | 897.2369 | 918.2056 | 988.7765 | 431.7008 | 446.8116 | 486.516 |
| 1056 | pos_441 | ethyl-2-methylacetoacetate | 163.1023 | 165.5939 | 178.2752 | 270.7053 | 279.4155 | 290.7878 | 464.839 | 512.616 | 550.6901 | 241.7488 | 268.3886 | 277.3217 |
| 1057 | pos_442 | Leukotriene D4 | 185.3546 | 203.5679 | 221.92 | 192.7677 | 200.7071 | 206.7612 | 240.4946 | 240.8424 | 254.7037 | 149.3902 | 158.7216 | 165.5103 |
| 1058 | pos_443 | L-histidinol | 1831.295 | 1904.744 | 1988.83 | 1792.779 | 1932.163 | 2119.198 | 1729.939 | 1889.522 | 1940.29 | 1873.205 | 1894.63 | 1969.945 |
| 1059 | pos_444 | Cer 18:0;3/18:0;1 | 9.64711 | 9.85164 | 10.42089 | 7.275301 | 7.749792 | 8.314246 | 1.219451 | 1.234265 | 1.285561 | 10.50615 | 10.77237 | 11.36999 |
| 1060 | pos_445 | Phytosphingosine 1-phosphate | 8578.574 | 8917.05 | 9093.891 | 8301.851 | 9115.798 | 9660.291 | 9237.415 | 9967.454 | 10518.55 | 8222.799 | 8916.15 | 9752.466 |
| 1061 | pos_446 | D-Ribulose 5-phosphate | 63.2264 | 68.34848 | 73.95106 | 69.14426 | 72.60589 | 77.39499 | 42.38961 | 46.88421 | 51.30439 | 104.2871 | 104.3805 | 109.7473 |
| 1062 | pos_447 | N-Succinyl-L-glutamate | 233.5392 | 253.9877 | 264.9833 | 106.9094 | 107.4985 | 111.6089 | 126.3461 | 135.3729 | 139.1245 | 100.4028 | 106.379 | 112.2676 |
| 1063 | pos_448 | 7-cyano-7-carbaguanine | 23.39049 | 23.39412 | 23.78109 | 30.64247 | 31.60556 | 33.27076 | 190.9436 | 197.4829 | 204.7982 | 7.776002 | 8.465977 | 8.992533 |
| 1064 | pos_449 | 5-methylaminomethyl-2-thiouridine | 530.5583 | 581.121 | 589.2619 | 2233.708 | 2346.925 | 2551.888 | 2411.366 | 2453.536 | 2693.443 | 2043.543 | 2248.904 | 2406.493 |
| 1065 | pos_450 | phenylalaninol | 91.25494 | 99.87324 | 105.5775 | 104.5732 | 109.7483 | 111.9932 | 96.87085 | 103.6672 | 108.0907 | 88.99454 | 94.54346 | 102.52 |
| 1066 | pos_451 | Pentanoic acid | 33.94631 | 35.56391 | 37.14825 | 25.66048 | 26.28127 | 26.98944 | 23.24174 | 25.262 | 26.19033 | 25.81074 | 26.62838 | 28.1943 |
| 1067 | pos_452 | Stearic acid-propyl ester | 0 | 0 | 0 | 0 | 0 | 0 | 426.722 | 466.958 | 503.9168 | 0 | 0 | 0 |
| 1068 | pos_453 | N2-Succinyl-L-ornithine | 204.3545 | 218.7427 | 225.7234 | 230.4763 | 237.8 | 258.0723 | 194.9979 | 215.5637 | 221.7836 | 192.935 | 211.7503 | 214.0528 |
| 1069 | pos_454 | (S)-3-Hydroxyisobutyryl-CoA | 0 | 0 | 0 | 297.7227 | 308.3778 | 330.1236 | 160.7036 | 165.2339 | 170.5188 | 150.9224 | 151.1838 | 163.0146 |
| 1070 | pos_455 | (1S,2R)-1-C-(indol-3-yl)glycerol 3-phosphate | 602.5872 | 650.1973 | 712.0161 | 4.998583 | 5.016518 | 5.216978 | 22.24059 | 24.38099 | 25.17369 | 1.741994 | 1.778191 | 1.788951 |
| 1071 | pos_456 | alpha-(2,6-anhydro-3-deoxy-D-arabino-heptulopyranosid)onate 7-phosphate | 35.45951 | 36.53485 | 39.40073 | 0 | 0 | 0 | 0 | 0 | 0 | 0 | 0 | 0 |
| 1072 | pos_457 | Guanine | 1392.855 | 1488.681 | 1584.175 | 322.1341 | 332.7631 | 351.6633 | 281.3299 | 311.792 | 315.4879 | 636.7393 | 706.8864 | 729.1145 |
| 1073 | pos_458 | Dihydrolipoamide | 30.59023 | 32.93218 | 34.47911 | 36.47352 | 39.45025 | 42.91941 | 43.45262 | 44.98331 | 47.4748 | 53.78584 | 54.80595 | 56.66065 |
| 1074 | pos_459 | N-Acetylneuraminic acid | 101.0668 | 106.5323 | 113.3872 | 36.866 | 37.43448 | 41.12451 | 24.73207 | 24.86871 | 25.04443 | 26.27495 | 26.62703 | 26.71979 |

Continued Table S2

| 1075 | pos_460 | 3-alpha,12-alpha-dihydroxy-7-oxo-5-beta-cholanate | 44.38404 | 45.34478 | 45.35521 | 41.91898 | 42.62781 | 45.00565 | 22.17117 | 24.51727 | 26.71239 | 35.17027 | 38.92877 | 40.39147 |
| --- | --- | --- | --- | --- | --- | --- | --- | --- | --- | --- | --- | --- | --- | --- |
| 1076 | pos_461 | (1/2,5,6)-2-(3-azibutylthio)-5,6-epoxy-3-cyclohexen-1-ol | 63.94605 | 63.97947 | 70.31574 | 59.02938 | 65.43554 | 67.28861 | 68.17724 | 71.5169 | 76.63806 | 69.93011 | 70.6337 | 75.3622 |
| 1077 | pos_462 | Malvidin glucoside-ethyl-catechin | 83.50417 | 86.19999 | 92.06526 | 0.684039 | 0.722638 | 0.735565 | 1.149587 | 1.211358 | 1.286256 | 0.292815 | 0.296639 | 0.31103 |
| 1078 | pos_463 | hexanoic acid | 147.9845 | 152.5213 | 160.1689 | 86.47781 | 92.55659 | 95.37317 | 115.4526 | 118.5102 | 120.7373 | 72.03553 | 73.76634 | 74.88886 |
| 1079 | pos_464 | D-4'-Phosphopantothenate | 102.7369 | 110.1286 | 115.9741 | 527.1896 | 570.3118 | 618.397 | 539.6072 | 574.6133 | 621.6781 | 593.0139 | 615.4467 | 625.6251 |
| 1080 | pos_465 | N-Acetyl-D-valine | 74.80401 | 78.19451 | 79.77096 | 79.49866 | 80.27944 | 84.49934 | 77.06787 | 84.87664 | 91.79454 | 72.74033 | 76.34294 | 82.8976 |
| 1081 | pos_466 | CDP-DG(18:0/16:0) | 245.6025 | 269.2325 | 292.8918 | 252.0311 | 256.2552 | 258.8459 | 469.2755 | 517.8814 | 555.251 | 250.1519 | 260.8843 | 279.7079 |
| 1082 | pos_467 | 2-Methylisoborneol | 23.56195 | 24.44858 | 26.1231 | 24.65101 | 26.5035 | 27.90166 | 23.94274 | 25.28575 | 27.31061 | 23.83229 | 26.24934 | 27.23367 |
| 1083 | pos_468 | PE(14:0/18:1(11Z)) | 329.2741 | 354.6496 | 377.8266 | 352.4263 | 365.1019 | 400.4448 | 508.7175 | 518.0955 | 535.5347 | 365.0721 | 370.3446 | 386.0111 |
| 1084 | pos_469 | PC(12:0/16:0) | 210.2414 | 210.786 | 223.0453 | 206.5965 | 211.8549 | 222.2334 | 603.1185 | 612.1233 | 646.5346 | 209.2148 | 210.1278 | 228.0322 |
| 1085 | pos_470 | Palmitic acid | 2302.787 | 2516.061 | 2683.453 | 2474.292 | 2702.678 | 2970.814 | 2377.548 | 2519.754 | 2731.581 | 2318.855 | 2441.416 | 2593.26 |
| 1086 | pos_471 | 1,5-dideoxy-1,5-imino-D-galactitol | 35.05956 | 37.8576 | 39.22444 | 27.81465 | 30.05101 | 32.53518 | 32.07049 | 34.14968 | 36.16259 | 23.68277 | 24.64139 | 26.20665 |
| 1087 | pos_472 | Oxoadipic acid | 73.82429 | 77.01146 | 78.25744 | 0 | 0 | 0 | 0 | 0 | 0 | 0 | 0 | 0 |
| 1088 | pos_473 | 3-dehydroquinate | 21.89867 | 23.38761 | 24.32308 | 0 | 0 | 0 | 0 | 0 | 0 | 0 | 0 | 0 |
| 1089 | pos_474 | L-Tyrosine | 352.6751 | 363.7637 | 376.5305 | 386.7668 | 405.3263 | 423.1481 | 380.9869 | 392.1425 | 392.6652 | 374.6544 | 396.8392 | 424.4927 |
| 1090 | pos_475 | N-Acetyl-D-phenylalanine | 28.01656 | 30.4855 | 32.39976 | 41.99192 | 42.26697 | 42.39064 | 38.66865 | 39.05957 | 39.41495 | 34.82053 | 38.13419 | 38.47271 |
| 1091 | pos_476 | 3-Oxo-7,8-dihydro-alpha-ionol | 2183.843 | 2255.52 | 2374.736 | 2440.137 | 2478.973 | 2724.536 | 1954.23 | 2152.066 | 2210.594 | 2425.761 | 2643.22 | 2726.701 |
| 1092 | pos_477 | PC(18:0/P-18:1(11Z)) | 105590.1 | 108565.2 | 115701.3 | 90305.1 | 98713.19 | 106737.7 | 98086.04 | 105774.6 | 114886.3 | 86581.18 | 87557.48 | 91243.45 |
| 1093 | pos_478 | Sorbic acid | 857.4212 | 898.7502 | 931.1302 | 663.028 | 713.7704 | 758.1124 | 513.4746 | 547.8882 | 555.1938 | 766.7538 | 770.2074 | 814.9395 |
| 1094 | pos_479 | 4-Glutathionyl cyclophosphamide | 72.02522 | 72.53452 | 77.31104 | 452.0529 | 467.1552 | 503.8066 | 349.7851 | 367.8136 | 384.3181 | 502.5031 | 535.8362 | 543.6061 |
| 1095 | pos_480 | 3a,7a,12b-Trihydroxy-5b-cholanoic acid | 53.62043 | 53.93777 | 58.7435 | 12.06541 | 13.18766 | 13.47669 | 3.216459 | 3.472105 | 3.774525 | 0 | 0 | 0 |
| 1096 | pos_481 | (2-trans,6-trans)-Farnesol | 534.0879 | 565.7671 | 601.6001 | 554.2953 | 609.4723 | 612.755 | 11718.41 | 12473.5 | 12867.85 | 522.7789 | 556.8474 | 561.8562 |
| 1097 | pos_482 | D-Urobilinogen | 4147.161 | 4151.459 | 4327.707 | 4476.829 | 4545.94 | 4933.085 | 3941.839 | 4359.668 | 4381.959 | 4133.545 | 4474.043 | 4752.503 |
| 1098 | pos_483 | N-amino DAP | 30.57493 | 30.62787 | 31.9613 | 37.98926 | 38.48652 | 39.03978 | 28.76918 | 30.03017 | 31.32334 | 34.71951 | 37.28825 | 38.53048 |

Continued Table S2

| 1099 | pos_484 | PS(17:0/16:0) | 1269.414 | 1332.882 | 1357.608 | 875.4016 | 897.187 | 980.6612 | 1206.226 | 1213.514 | 1235.262 | 759.9365 | 826.901 | 874.0543 |
| --- | --- | --- | --- | --- | --- | --- | --- | --- | --- | --- | --- | --- | --- | --- |
| 1100 | pos_485 | PC(18:3(9Z,12Z,15Z)/22:1(13Z)) | 12034.36 | 12347.84 | 12397.3 | 6640.79 | 7110.02 | 7791.993 | 10766.85 | 11561.11 | 11679.39 | 5655.913 | 5799.646 | 6114.412 |
| 1101 | pos_486 | PC(18:4(6Z,9Z,12Z,15Z)/20:2(11Z,14Z)) | 2742.063 | 2904.278 | 3072.867 | 1170.334 | 1179.651 | 1261.545 | 2871.365 | 2980.497 | 3039.262 | 795.0263 | 814.2275 | 841.5409 |
| 1102 | pos_487 | Heptanoic acid | 38.59969 | 42.75612 | 43.27841 | 45.83043 | 47.25263 | 48.48461 | 37.91956 | 40.67235 | 41.76031 | 52.42263 | 52.61457 | 54.06732 |
| 1103 | pos_488 | S-Adenosylmethionine | 135.0884 | 145.9272 | 159.9246 | 166.7445 | 171.1974 | 184.015 | 142.1736 | 157.8872 | 173.0902 | 137.5639 | 146.2208 | 159.829 |
| 1104 | pos_489 | 2-(S-Glutathionyl)acetyl glutathione | 0 | 0 | 0 | 65.98598 | 70.12895 | 74.59211 | 27.10589 | 28.69754 | 29.08901 | 60.98593 | 65.80187 | 69.84325 |
| 1105 | pos_490 | Pyrocatechol | 39.89927 | 42.55627 | 43.72085 | 62.71952 | 69.47359 | 69.89513 | 62.57862 | 65.15129 | 65.1703 | 64.18613 | 65.27362 | 66.1658 |
| 1106 | pos_491 | N-iodoacetylglucosamine 6-phosphate | 630.7466 | 676.9167 | 689.4703 | 0 | 0 | 0 | 0 | 0 | 0 | 0 | 0 | 0 |
| 1107 | pos_492 | tryptophanhydroxamate | 4.575469 | 4.638812 | 4.955477 | 5.013029 | 5.337277 | 5.367058 | 2.560358 | 2.598284 | 2.680963 | 8.686647 | 9.059958 | 9.394393 |
| 1108 | pos_493 | L-Glutamic acid 5-phosphate | 33.88601 | 34.32441 | 36.74559 | 18.65136 | 18.71742 | 19.43524 | 34.16676 | 36.62952 | 38.02189 | 14.60336 | 15.15501 | 15.50172 |
| 1109 | pos_494 | L-histidinol phosphate | 25.39925 | 25.6646 | 27.5456 | 9.329287 | 9.977129 | 10.50749 | 16.75169 | 17.37749 | 18.47639 | 6.986667 | 7.31897 | 7.638414 |
| 1110 | pos_495 | Spermine | 11288.46 | 11700.75 | 12159.17 | 11176.36 | 12398.19 | 13492.83 | 11582.78 | 11605.56 | 12739.51 | 10566.63 | 11385.61 | 11875.39 |
| 1111 | pos_496 | cis-3-hexen-1-ol | 123.8352 | 135.3831 | 147.0437 | 129.0828 | 131.4812 | 138.6627 | 998.1054 | 1065.519 | 1152.229 | 125.4745 | 135.7771 | 139.9325 |
| 1112 | pos_497 | Alpha-Linolenic acid | 252.5012 | 271.4399 | 272.7142 | 297.6935 | 327.5231 | 335.0829 | 272.8863 | 302.8556 | 318.38 | 241.4644 | 267.3741 | 270.4537 |
| 1113 | pos_498 | Galactosylglycerol | 18.90781 | 20.99744 | 22.14401 | 184.7439 | 193.5551 | 212.6242 | 179.5588 | 188.1067 | 191.1062 | 172.6785 | 190.0916 | 199.4295 |
| 1114 | pos_499 | L-Aspartic acid | 4949.548 | 5097.09 | 5382.944 | 599.756 | 624.5662 | 658.1098 | 4003.043 | 4213.756 | 4382.468 | 330.0498 | 355.8199 | 377.4052 |
| 1115 | pos_500 | Fertaric acid | 35.63491 | 36.1617 | 39.3059 | 125.8136 | 126.3595 | 136.413 | 57.77904 | 58.43593 | 59.71875 | 143.8079 | 146.5303 | 148.0175 |
| 1116 | pos_501 | Undecanol | 49.39085 | 49.47125 | 53.65859 | 56.89468 | 58.70306 | 61.73709 | 51.12023 | 53.77521 | 58.8412 | 50.16839 | 50.38254 | 52.00787 |
| 1117 | pos_502 | D-penicillamine | 163.0088 | 179.2284 | 196.7373 | 193.3529 | 203.7933 | 211.4391 | 174.3371 | 185.8704 | 189.0228 | 170.8685 | 174.7916 | 174.9333 |
| 1118 | pos_503 | N-acetyl-D-glucosamine 6-phosphate | 13.86623 | 14.56398 | 15.44925 | 9.965306 | 10.03046 | 10.39776 | 8.977716 | 9.638389 | 10.54906 | 8.712666 | 8.819114 | 8.855331 |
| 1119 | pos_504 | 2-formamido-N(1)-(5-phospho-D-ribosyl)acetamidine | 765.5553 | 839.127 | 885.0808 | 13.71857 | 14.14091 | 14.2959 | 57.2125 | 58.19728 | 63.13176 | 33.51622 | 35.3514 | 35.60274 |
| 1120 | pos_505 | UDP-2,3-bis(3-hydroxytetradecanoyl)glucosamine | 188.8084 | 204.3432 | 206.9499 | 23.37142 | 25.50344 | 26.2603 | 50.18849 | 51.93997 | 53.76814 | 19.82964 | 21.4205 | 21.52868 |
| 1121 | pos_506 | N2-hydroxyguanosine 5'-monophosphate | 127.7826 | 135.9713 | 146.1671 | 71.12248 | 71.16095 | 73.90565 | 67.48576 | 68.776 | 73.04604 | 68.55703 | 74.33397 | 77.24115 |
| 1122 | pos_507 | N-Acetylmannosamine | 2716.31 | 2858.676 | 3143.66 | 2719.196 | 2922.536 | 3050.407 | 2824.959 | 2898.433 | 3033.564 | 2792.833 | 2808.787 | 2977.313 |

Continued Table S2

| 1123 | pos_508 | L-Ala-gamma-D-Glu-Dap | 6.873749 | 7.252054 | 7.271963 | 27.31562 | 29.45047 | 29.55302 | 8.141358 | 8.49692 | 9.21939 | 24.9555 | 26.92805 | 27.39273 |
| --- | --- | --- | --- | --- | --- | --- | --- | --- | --- | --- | --- | --- | --- | --- |
| 1124 | pos_509 | N-acetylmuramate(beta-methyl)-L-alanyl-D-glutamate | 0 | 0 | 0 | 0 | 0 | 0 | 144.4204 | 150.8233 | 154.1171 | 0 | 0 | 0 |
| 1125 | pos_510 | Menthol | 72.96463 | 75.16129 | 82.44692 | 64.03311 | 68.49169 | 71.41117 | 58.43381 | 64.17887 | 69.21588 | 87.27477 | 87.35902 | 92.39367 |
| 1126 | pos_511 | PA(P-16:0e/18:2(9Z,12Z)) | 24.73175 | 25.34671 | 27.37072 | 18.56911 | 20.0381 | 20.59591 | 8.989107 | 9.451056 | 10.25801 | 28.77984 | 29.91248 | 30.01921 |
| 1127 | pos_512 | polymixin B | 295.3508 | 309.2771 | 324.7598 | 341.1487 | 344.6774 | 356.068 | 375.9113 | 393.9108 | 428.9869 | 331.2111 | 365.2705 | 386.5511 |
| 1128 | pos_513 | Linoleic acid | 0 | 0 | 0 | 0 | 0 | 0 | 38.67835 | 41.36589 | 44.30996 | 0 | 0 | 0 |
| 1129 | pos_514 | piericidin A | 56.99745 | 60.2891 | 63.19786 | 49.25454 | 50.04507 | 52.36792 | 47.13969 | 47.27588 | 51.47069 | 48.02361 | 51.53417 | 53.09512 |
| 1130 | pos_515 | Dimethyl-L-arginine | 6.248213 | 6.628576 | 7.030305 | 74.25653 | 75.82277 | 78.04204 | 29.7372 | 31.52583 | 31.68647 | 52.90875 | 56.60593 | 58.90471 |
| 1131 | pos_516 | 4-Vinylguaiacol | 59.32263 | 64.248 | 68.35171 | 122.1892 | 130.1656 | 141.2415 | 107.2447 | 117.3982 | 123.2254 | 116.0414 | 127.3973 | 137.1403 |
| 1132 | pos_517 | D,L- gamma-hydroxyphosphinothricin | 0 | 0 | 0 | 0 | 0 | 0 | 45.75294 | 49.84993 | 54.82443 | 0 | 0 | 0 |
| 1133 | pos_518 | Glutaryl-CoA | 180.4477 | 197.0281 | 210.3722 | 83.9424 | 87.82368 | 88.94921 | 115.9475 | 123.6727 | 132.8768 | 75.60756 | 79.89744 | 81.69585 |
| 1134 | pos_519 | Phenyl acetate | 24.50149 | 26.43968 | 27.51398 | 14.79945 | 16.3411 | 16.49441 | 13.01266 | 14.33655 | 15.27206 | 12.54475 | 13.50565 | 13.53135 |
| 1135 | pos_520 | gamma-butyrolactone | 62.09958 | 65.92309 | 67.05024 | 67.2953 | 68.96045 | 72.56057 | 44.15806 | 49.02963 | 53.72214 | 63.3987 | 67.36971 | 73.91537 |
| 1136 | pos_521 | Phenylethylacetate | 8.64127 | 9.543552 | 9.956809 | 10.21965 | 10.83835 | 10.86004 | 10.33451 | 10.7776 | 11.60859 | 10.2258 | 10.34984 | 10.96992 |
| 1137 | pos_522 | L-gamma-Glutamyl-L-alanine | 338.5964 | 342.82 | 363.9528 | 347.7486 | 351.2646 | 370.9615 | 324.7651 | 334.2806 | 347.0151 | 298.8023 | 325.8482 | 357.3738 |
| 1138 | pos_523 | UDP-N-acetylmuramoyl-L-alanyl-D-gamma-glutamyl-meso-2,6-diaminopimelate | 491.5734 | 541.2575 | 589.7972 | 72.02665 | 76.12332 | 80.77043 | 56.49826 | 56.80777 | 57.82946 | 106.4955 | 116.209 | 118.8427 |
| 1139 | pos_524 | (3S)-3-Hydroxyadipyl-CoA | 0 | 0 | 0 | 32.34621 | 33.99297 | 36.19577 | 23.16017 | 23.96071 | 25.48894 | 26.3302 | 28.70469 | 29.33631 |
| 1140 | pos_525 | Ethyl octanoate | 51.48352 | 55.96921 | 58.12657 | 59.70681 | 61.84087 | 67.88929 | 198.39 | 203.7099 | 215.5494 | 56.09441 | 56.7653 | 58.59865 |
| 1141 | pos_526 | PE(14:1(9Z)/16:0) | 2708.965 | 2810.301 | 3064.905 | 2767.275 | 2890.377 | 3141.621 | 3605.902 | 3946.697 | 4234.562 | 2581.911 | 2828.618 | 2904.137 |
| 1142 | pos_527 | PA(14:0/0:0) | 420.6667 | 461.046 | 505.3934 | 486.8962 | 532.2441 | 580.4192 | 694.478 | 717.2627 | 779.2587 | 456.0919 | 483.6695 | 498.5272 |
| 1143 | pos_528 | (2S)-2-Hydroxy-3-oxobutyl phosphate | 31.84012 | 33.98578 | 35.15089 | 34.98764 | 38.56377 | 40.6969 | 34.38391 | 34.97575 | 35.2459 | 36.72712 | 40.63276 | 41.31487 |
| 1144 | pos_529 | N6-(1,2-Dicarboxyethyl)-AMP | 23.49637 | 24.81243 | 25.62163 | 21.27338 | 22.78425 | 24.78838 | 22.78933 | 24.86733 | 26.59203 | 17.98094 | 18.01441 | 19.63169 |
| 1145 | pos_530 | trans-2,3-Dihydroxycinnamate | 1984.256 | 2145.06 | 2273.876 | 77.20928 | 83.96478 | 87.2575 | 295.8794 | 305.2831 | 309.4374 | 73.35164 | 77.69542 | 79.24935 |
| 1146 | pos_531 | N-delta-(phosphonoacetyl)-L-ornithine | 34.56457 | 35.98897 | 36.66289 | 57.26913 | 60.80886 | 66.66006 | 97.22006 | 99.22923 | 106.6523 | 43.43321 | 47.447 | 50.78581 |

Continued Table S2

| 1147 | pos_532 | alpha-methylmethionine | 3032.069 | 3233.466 | 3410.003 | 1414.179 | 1445.594 | 1553.461 | 1013.115 | 1122.818 | 1187.488 | 1117.872 | 1236.947 | 1281.348 |
| --- | --- | --- | --- | --- | --- | --- | --- | --- | --- | --- | --- | --- | --- | --- |
| 1148 | pos_533 | S-Methyl-5-thio-alpha-D-ribose 1-phosphate | 409.4 | 413.6509 | 438.8305 | 345.3267 | 375.5615 | 389.519 | 100.7205 | 111.3149 | 114.2771 | 394.2443 | 426.3083 | 457.2516 |
| 1149 | pos_534 | Phosphoenolpyruvic acid | 27.34681 | 29.55063 | 30.1499 | 29.71374 | 30.09171 | 32.53381 | 25.25777 | 27.76926 | 29.57665 | 27.50457 | 29.64477 | 31.63154 |
| 1150 | pos_535 | 3-(3-hydroxyphenyl)propionate | 1962.524 | 2014.348 | 2036.577 | 99.96603 | 110.7403 | 110.8934 | 1114.644 | 1142.471 | 1172.883 | 83.87484 | 87.21245 | 92.8265 |
| 1151 | pos_536 | TG(18:0/18:0/18:1(9Z)) | 208.4947 | 216.9151 | 224.6563 | 362.0468 | 368.0277 | 373.2205 | 181.5114 | 185.6819 | 198.7395 | 298.2384 | 328.8004 | 339.0927 |
| 1152 | pos_537 | L-Arginine | 457.0234 | 458.2116 | 486.7664 | 441.0772 | 480.272 | 520.4584 | 439.5091 | 454.1412 | 487.3745 | 431.3077 | 460.2775 | 472.8884 |
| 1153 | pos_538 | Phenylacetic acid | 484.6443 | 517.6731 | 528.7818 | 7.64895 | 7.773883 | 7.932963 | 6.773162 | 6.939254 | 7.141011 | 10.2912 | 10.6877 | 11.20922 |
| 1154 | pos_539 | beta-D-Galactose | 79.19592 | 83.09313 | 87.99731 | 175.3496 | 180.6663 | 180.8518 | 78.8348 | 83.67134 | 90.97866 | 211.2788 | 219.1605 | 221.9077 |
| 1155 | pos_540 | MurNAc-6-P | 2012.363 | 2027.993 | 2084.677 | 84.10097 | 88.32383 | 90.64536 | 98.57877 | 109.4275 | 114.0948 | 69.83962 | 74.68478 | 76.18778 |
| 1156 | pos_541 | 2-Isopropylmalic acid | 47.12875 | 51.25009 | 54.00957 | 243.9086 | 245.4649 | 251.905 | 322.5281 | 337.0913 | 352.8798 | 102.0843 | 112.1331 | 112.9778 |
| 1157 | pos_542 | Tetrahydrofolic acid | 111.0261 | 120.5471 | 123.5016 | 95.43299 | 100.1932 | 107.0168 | 75.46071 | 77.1496 | 80.4699 | 91.06891 | 99.3958 | 106.9479 |
| 1158 | pos_543 | dihydro-UDP-N-acetylmuramate | 591.9546 | 621.698 | 653.3423 | 0 | 0 | 0 | 0 | 0 | 0 | 0 | 0 | 0 |
| 1159 | pos_544 | Deoxyuridine triphosphate | 50.1653 | 51.49066 | 53.72254 | 24.23145 | 26.12023 | 26.74042 | 127.7901 | 133.0283 | 145.0009 | 17.74997 | 18.90559 | 19.04064 |
| 1160 | pos_545 | LIPC 18:0;3 | 19.98221 | 21.27523 | 22.8546 | 31.56748 | 32.18932 | 34.18158 | 1063.689 | 1142.804 | 1143.494 | 23.21411 | 25.419 | 26.55882 |
| 1161 | pos_546 | Deoxycytidine | 349.0475 | 350.9487 | 369.5275 | 351.9719 | 377.1567 | 409.4626 | 292.8153 | 321.2416 | 330.7053 | 363.454 | 377.6967 | 414.8492 |
| 1162 | pos_547 | CDP-DG(16:0/18:1(9Z)) | 47.12144 | 49.25899 | 53.16224 | 56.81138 | 59.59242 | 63.84051 | 79.5024 | 87.76869 | 92.30914 | 48.41213 | 50.60654 | 55.51794 |
| 1163 | pos_548 | PC(18:0/18:0) | 2456.176 | 2523.034 | 2663.856 | 2401.2 | 2516.847 | 2734.785 | 3340.221 | 3361.308 | 3493.792 | 2244.759 | 2404.23 | 2546.388 |
| 1164 | pos_549 | decoyinine | 123987.6 | 124290.1 | 129346.6 | 123340.9 | 123804.2 | 124431.6 | 112579.9 | 122424.7 | 125889.7 | 116633.2 | 120162.6 | 132096.4 |
| 1165 | pos_550 | 4-methylene DAP | 274.3111 | 289.6377 | 296.4648 | 283.2045 | 301.9223 | 331.7074 | 270.5988 | 272.7797 | 283.9605 | 242.5253 | 262.5729 | 272.5479 |
| 1166 | pos_551 | 2-(3-Carboxy-3-(methylammonio)propyl)-L-histidine | 90.39122 | 94.95254 | 102.3177 | 87.15635 | 91.44807 | 97.73716 | 102.6478 | 103.5211 | 107.0914 | 86.27109 | 90.58492 | 92.12283 |
| 1167 | pos_552 | Porphyrin-ring | 224.532 | 233.2631 | 233.3224 | 484.5614 | 490.8713 | 497.0323 | 482.2977 | 530.983 | 548.4633 | 512.9515 | 520.9863 | 566.3127 |
| 1168 | pos_553 | trans-2-Enoyl-OPC4-CoA | 12.20827 | 13.17387 | 13.2364 | 54.74394 | 54.77342 | 55.54558 | 51.32278 | 55.71784 | 60.26107 | 61.27605 | 65.91184 | 69.26801 |
| 1169 | pos_554 | dTDP | 118.2545 | 128.1805 | 136.503 | 2.551412 | 2.593401 | 2.75499 | 3.65286 | 3.786348 | 3.897675 | 0 | 0 | 0 |
| 1170 | pos_555 | ADP-D-glycero-D-manno-heptose | 0.793666 | 0.83704 | 0.871286 | 223.7957 | 232.3482 | 252.2495 | 174.5317 | 184.7721 | 194.1762 | 248.1631 | 261.2365 | 281.3465 |
| 1171 | pos_556 | 2-thiouridine | 169.5919 | 177.069 | 182.8609 | 0 | 0 | 0 | 1.333802 | 1.358855 | 1.460125 | 0 | 0 | 0 |

Continued Table S2

| 1172 | pos_557 | O-Acetylserine | 47.05598 | 51.20266 | 53.76524 | 18.81501 | 19.974 | 21.61866 | 14.41087 | 15.651 | 16.42162 | 22.95037 | 24.55099 | 25.62628 |
| --- | --- | --- | --- | --- | --- | --- | --- | --- | --- | --- | --- | --- | --- | --- |
| 1173 | pos_558 | dCDP | 113.1673 | 119.4459 | 123.3119 | 68.75692 | 72.28598 | 73.04387 | 44.49882 | 48.86004 | 53.08133 | 87.90209 | 95.55424 | 102.6834 |
| 1174 | pos_559 | S-hexyl glutathione | 11.14201 | 12.20269 | 13.40152 | 185.4781 | 201.5585 | 218.659 | 3.73858 | 3.97685 | 4.195495 | 84.35952 | 90.99205 | 91.45845 |
| 1175 | pos_560 | 4-Amino-4-deoxychorismate | 829.7328 | 865.702 | 900.0681 | 4.071365 | 4.161188 | 4.460271 | 0 | 0 | 0 | 0 | 0 | 0 |
| 1176 | pos_561 | L-Aspartate-semialdehyde | 0 | 0 | 0 | 0.722044 | 0.776035 | 0.787854 | 0.38249 | 0.399615 | 0.4259 | 1.710302 | 1.771142 | 1.91927 |
| 1177 | pos_562 | cerotic acid | 1115.102 | 1124.428 | 1223.106 | 1098.008 | 1189.577 | 1258.842 | 1223.3 | 1338.513 | 1457.613 | 1037.843 | 1150.949 | 1256.537 |
| 1178 | pos_563 | (2R,3R)-2,3-Dihydroxy-3-methylpentanoate | 89.10957 | 93.38792 | 101.7109 | 90.46514 | 95.73861 | 99.34536 | 85.47898 | 86.74971 | 90.55997 | 95.03594 | 97.73022 | 103.2591 |
| 1179 | pos_564 | Palmitaldehyde | 2387.417 | 2538.528 | 2771.117 | 2723.698 | 2913.239 | 3065.087 | 2335.686 | 2540.226 | 2740.067 | 2115.514 | 2338.599 | 2359.317 |
| 1180 | pos_565 | Cyanidin 3-O-(acetylglucoside) | 14.95062 | 15.81801 | 16.55152 | 31.29226 | 33.33013 | 36.59802 | 6.127005 | 6.645656 | 6.729182 | 29.5914 | 30.25811 | 33.24235 |
| 1181 | pos_566 | Purine | 1127.804 | 1222.606 | 1273.528 | 3833.651 | 3959.322 | 4157.564 | 1543.006 | 1605.891 | 1662.034 | 4008.008 | 4320.276 | 4343.857 |
| 1182 | pos_567 | N5-Pan | 561.5976 | 576.5643 | 578.6178 | 551.1451 | 592.0134 | 638.5878 | 558.6069 | 589.0571 | 620.4732 | 598.0247 | 605.1728 | 653.8939 |
| 1183 | pos_568 | PG(16:1(9Z)/18:1(9Z)) | 795.4424 | 802.1096 | 838.4936 | 805.156 | 844.3999 | 851.7477 | 1034.42 | 1039.376 | 1077.219 | 737.7588 | 762.4919 | 814.3441 |
| 1184 | pos_569 | Homovanillic acid | 166.6203 | 182.0119 | 193.8549 | 191.1872 | 202.12 | 219.7371 | 181.3999 | 197.8307 | 197.8955 | 183.086 | 193.4211 | 210.3778 |
| 1185 | pos_570 | 7,8-Dihydro-7-hydroxy-8-S-glutathionyl-benzo[a]pyrene | 68.9754 | 72.69609 | 75.50332 | 48.02617 | 48.13846 | 52.89763 | 69.54404 | 71.79768 | 73.01723 | 54.92142 | 56.11542 | 61.06759 |
| 1186 | pos_571 | FADH | 27.47848 | 30.36966 | 32.31057 | 140.5525 | 146.3903 | 149.504 | 226.7716 | 236.8502 | 250.3193 | 92.97706 | 101.4641 | 103.936 |
| 1187 | pos_572 | 2,5-diamino-6-hydroxy-4-(5-phosphoribosylamino)pyrimidine | 0 | 0 | 0 | 177.7298 | 178.2308 | 188.4865 | 154.7818 | 163.7904 | 168.0447 | 138.8869 | 147.0839 | 150.5909 |
| 1188 | pos_573 | 3-carboxy-1-hydroxypropylthiamine diphosphate | 157.7208 | 168.0985 | 178.0673 | 15.45471 | 16.12437 | 17.3838 | 45.35538 | 45.46746 | 49.16504 | 15.48969 | 15.80598 | 16.27415 |
| 1189 | pos_574 | beta-ketophosphonate | 89.10273 | 95.72158 | 99.08875 | 61.03356 | 66.08633 | 70.90072 | 108.1132 | 117.1463 | 118.6449 | 48.94313 | 53.22225 | 57.58735 |
| 1190 | pos_575 | Tetrahydrodipicolinate | 52.83123 | 58.43169 | 62.47298 | 44.88802 | 47.78068 | 48.55328 | 7.086545 | 7.217656 | 7.692353 | 37.01011 | 38.33011 | 38.67991 |
| 1191 | pos_576 | PC(18:4(6Z,9Z,12Z,15Z)/dm18:0) | 2442.454 | 2496.901 | 2561.489 | 1947.151 | 2111.692 | 2288.271 | 2314.171 | 2406.687 | 2421.631 | 1810.087 | 1905.473 | 1982.46 |
| 1192 | pos_577 | D-mannosamine | 14.10056 | 14.47407 | 15.1947 | 13.71648 | 15.12276 | 15.60056 | 17.64992 | 17.72153 | 18.99202 | 12.62813 | 13.72608 | 13.95872 |
| 1193 | pos_578 | 1,2-Dimethoxy-3-propylbenzene | 39.32769 | 41.16292 | 43.62785 | 42.15728 | 46.18116 | 47.5252 | 36.58926 | 36.69396 | 39.97998 | 35.5664 | 37.07119 | 37.78865 |
| 1194 | pos_579 | PE(14:0/14:0) | 0 | 0 | 0 | 0 | 0 | 0 | 130.9228 | 138.4008 | 141.7781 | 0 | 0 | 0 |

Continued Table S2

| 1195 | pos_580 | Tyramine | 38.56372 | 39.20992 | 39.86945 | 37.79785 | 39.45867 | 40.16306 | 32.11118 | 35.17237 | 35.84339 | 43.94674 | 46.64674 | 48.32137 |
| --- | --- | --- | --- | --- | --- | --- | --- | --- | --- | --- | --- | --- | --- | --- |
| 1196 | pos_581 | alpha-D-glucose 1-methylene-phosphonate | 158.7207 | 167.6237 | 170.1471 | 133.6889 | 140.2934 | 147.7715 | 98.25131 | 108.4318 | 111.8033 | 145.3835 | 149.2133 | 152.1725 |
| 1197 | pos_582 | N-(5-Phospho-D-ribosyl)anthranilate | 2227.611 | 2420.374 | 2440.169 | 3.386408 | 3.697699 | 3.841222 | 13.41272 | 14.46415 | 15.73395 | 3.075631 | 3.227354 | 3.257028 |
| 1198 | pos_583 | CDP-ribitol | 19.07232 | 19.22612 | 20.9123 | 18.72012 | 20.34061 | 20.39907 | 3.773947 | 4.010631 | 4.398193 | 25.39363 | 26.59854 | 27.78166 |
| 1199 | pos_584 | 2'-Deoxyadenosine | 0 | 0 | 0 | 0 | 0 | 0 | 77.22298 | 78.92095 | 86.71867 | 0 | 0 | 0 |
| 1200 | pos_585 | 4-Methylthiazole | 87.53785 | 88.63761 | 93.2665 | 81.55958 | 87.96767 | 94.0534 | 85.1122 | 92.91777 | 98.39463 | 83.22669 | 88.57682 | 89.32503 |
| 1201 | pos_586 | Dihydrothymine | 1.417816 | 1.541333 | 1.694815 | 4.881363 | 5.273232 | 5.478532 | 3.550217 | 3.911552 | 4.047303 | 4.331838 | 4.698195 | 4.996233 |
| 1202 | pos_587 | (2E)-2-(methoxycarbonylmethyl)but-2-enedioic acid | 7879.11 | 8064.724 | 8275.751 | 136.7007 | 139.1911 | 144.1435 | 120.1517 | 129.489 | 141.6435 | 102.9214 | 112.6492 | 121.8142 |
| 1203 | pos_588 | phenyl-1-thio-beta-D-galactopyranoside | 183.2145 | 183.6137 | 192.5553 | 117.0027 | 124.2718 | 125.9685 | 60.53622 | 65.77736 | 67.00766 | 88.10956 | 97.2621 | 102.988 |
| 1204 | pos_589 | 2-Methyl-5-propylpyrazine | 104.4869 | 113.2351 | 117.1843 | 16.6346 | 17.76441 | 18.42391 | 5.120124 | 5.630174 | 5.833674 | 7.910216 | 8.228951 | 8.953306 |
| 1205 | pos_590 | Arbutin 6-phosphate | 93.39591 | 97.99686 | 98.47936 | 4.546657 | 4.655725 | 4.83191 | 4.981078 | 5.080113 | 5.197308 | 0 | 0 | 0 |
| 1206 | pos_591 | decan-1-ol | 6003.183 | 6248.578 | 6618.82 | 5136.992 | 5155.193 | 5411.041 | 5469.058 | 5509.049 | 5778.01 | 4548.978 | 4965.825 | 5416.447 |
| 1207 | pos_592 | 2,6-Dimethyl-1,7-octadien-3,6-diol | 1904.211 | 1994.327 | 2145.191 | 2022.556 | 2069.059 | 2122.236 | 14625.49 | 15565.81 | 15661.62 | 2080.135 | 2087.611 | 2255.753 |
| 1208 | pos_593 | Phenylethylamine | 345.9869 | 374.019 | 407.7103 | 493.8049 | 501.8391 | 544.1733 | 371.4673 | 395.4514 | 414.2874 | 454.0602 | 473.0792 | 493.2629 |
| 1209 | pos_594 | 7-Methylguanine | 40.23302 | 42.10319 | 46.07446 | 48.33227 | 50.72094 | 52.61952 | 52.60477 | 56.65691 | 60.44819 | 46.58027 | 48.96863 | 49.34608 |
| 1210 | pos_595 | Uridine 5'-monophosphate | 70.93138 | 73.02459 | 74.54534 | 3.461731 | 3.472695 | 3.55949 | 12.56375 | 13.46454 | 13.82658 | 1.603733 | 1.682267 | 1.837764 |
| 1211 | pos_596 | CDP-DG(16:0/22:6(4Z,7Z,10Z,13Z,16Z,19Z)) | 208.0644 | 222.1678 | 222.8842 | 198.723 | 202.4259 | 203.1111 | 326.4271 | 336.8864 | 360.3723 | 194.3908 | 197.7777 | 204.9034 |
| 1212 | pos_597 | Benzaldehyde-cyanohydrin | 81.76119 | 83.57774 | 84.76488 | 66.47621 | 73.54157 | 76.0838 | 78.57989 | 86.39903 | 94.26157 | 64.6074 | 65.65419 | 69.90219 |
| 1213 | pos_598 | 5-Methylthioribose | 41.84238 | 42.87468 | 46.74345 | 85.42707 | 92.57309 | 98.03045 | 94.63003 | 103.5676 | 109.8063 | 108.2056 | 109.8985 | 110.569 |
| 1214 | pos_599 | L-Tryptophan | 20.29783 | 21.15066 | 23.06299 | 11.5003 | 12.10566 | 12.35026 | 9.975833 | 10.64245 | 10.77839 | 3.773591 | 4.183393 | 4.588848 |
| 1215 | pos_600 | trans-Isohumulone | 332.8357 | 346.7673 | 381.2413 | 319.0633 | 338.1357 | 346.7191 | 390.0268 | 422.4281 | 431.1137 | 306.785 | 337.7363 | 352.8323 |
| 1216 | pos_601 | PC(14:1(9Z)/22:2(13Z,16Z)) | 2001.415 | 2038.56 | 2083.377 | 1499.811 | 1644.239 | 1770.833 | 1959.882 | 1995.385 | 2025.166 | 1309.6 | 1440.458 | 1521.861 |
| 1217 | pos_602 | ethylglyoxalbis(guanylhydrazone) | 230.0378 | 236.0317 | 254.2731 | 20.03198 | 20.81454 | 22.46471 | 392.0964 | 429.1163 | 432.2878 | 19.99378 | 20.04972 | 21.37655 |
| 1218 | pos_603 | cephalexin | 1163.118 | 1259.872 | 1296.057 | 701.3587 | 727.8111 | 735.0006 | 1449.695 | 1566.115 | 1707.853 | 705.984 | 742.5576 | 749.6946 |

Continued Table S2

| 1219 | pos_604 | tridecaprenyl diphosphate | 311.1687 | 331.9041 | 333.7261 | 391.2049 | 422.3128 | 463.8185 | 630.4404 | 640.7697 | 644.4867 | 460.3678 | 495.3733 | 532.6539 |
| --- | --- | --- | --- | --- | --- | --- | --- | --- | --- | --- | --- | --- | --- | --- |
| 1220 | pos_605 | 9-[6(S),9-diamino-5,6,7,8,9-pentadeoxy-beta-D-ribo-nonafuranosyl]-9H-purin-6-amine | 37.29021 | 38.48544 | 42.05937 | 71.47865 | 76.07051 | 80.18905 | 242.4495 | 260.4105 | 276.2337 | 54.07656 | 56.11637 | 57.22154 |
| 1221 | pos_606 | 4-Hexanolide | 327.7053 | 360.585 | 380.5534 | 338.0976 | 369.2089 | 375.1535 | 359.4856 | 366.187 | 388.3357 | 345.269 | 357.0791 | 358.4944 |
| 1222 | pos_607 | PC(14:0/20:2(11Z,14Z)) | 2235.457 | 2394.978 | 2508.865 | 1486.295 | 1486.58 | 1592.86 | 4317.545 | 4776.367 | 5150.281 | 803.8862 | 875.8397 | 907.564 |
| 1223 | pos_608 | Cyanidin | 252.1756 | 275.0569 | 277.3607 | 26.44219 | 29.03283 | 29.33437 | 9.964154 | 11.02564 | 11.682 | 16.79414 | 18.62455 | 19.42286 |
| 1224 | pos_609 | thiazolidine | 420.4628 | 434.2146 | 472.8673 | 375.9407 | 391.2507 | 410.2588 | 381.4294 | 397.5588 | 411.4217 | 378.8822 | 384.2866 | 420.1753 |
| 1225 | pos_610 | bacimethrin | 81.62067 | 88.23421 | 90.05729 | 92.77233 | 93.61106 | 99.95924 | 82.08486 | 89.16881 | 93.39599 | 94.60198 | 99.56236 | 101.7297 |
| 1226 | pos_611 | Pyridoxal | 410.6959 | 435.4513 | 453.3052 | 408.9266 | 440.6367 | 467.1786 | 513.8492 | 548.8425 | 570.5643 | 425.2628 | 467.4626 | 484.9798 |
| 1227 | pos_612 | Pyrazinecarboxamide | 0 | 0 | 0 | 162.2227 | 178.7441 | 196.2861 | 346.4816 | 377.9203 | 400.2033 | 194.8165 | 208.6352 | 227.5068 |
| 1228 | pos_613 | UDP-N-acetylmuramoyl-L-alanyl-gamma-D-glutamyl-L-lysine | 0 | 0 | 0 | 153.8485 | 155.6089 | 156.4553 | 171.9961 | 187.9334 | 193.1108 | 177.1743 | 194.5811 | 196.8002 |
| 1229 | pos_614 | 2-Hydroxy-cis-hex-2,4-dienoate | 17790.29 | 17808.47 | 19181.3 | 17625.87 | 18294.38 | 19577.02 | 17010.64 | 17477.88 | 17599.38 | 16206.64 | 17694.64 | 19382.49 |
| 1230 | pos_615 | Dimethyl sulfide | 28.46037 | 30.88051 | 33.58417 | 22.96074 | 25.14302 | 26.52643 | 25.33184 | 26.28875 | 28.12659 | 26.3039 | 27.99197 | 30.75035 |
| 1231 | pos_616 | 3-ethoxy-1-propanol | 94.75281 | 102.0462 | 102.1522 | 82.26709 | 88.55658 | 88.87998 | 144.3623 | 157.4107 | 163.5932 | 83.39887 | 86.95513 | 88.29374 |
| 1232 | pos_617 | 3-(4-hydroxyphenyl)lactic acid | 144.9956 | 146.4699 | 150.2827 | 1.191113 | 1.312607 | 1.335942 | 6.737621 | 6.802189 | 6.906729 | 0.017704 | 0.019667 | 0.021115 |
| 1233 | pos_618 | Dodecenoylcarnitine | 42.97963 | 45.07885 | 47.86548 | 52.71849 | 55.36736 | 55.79041 | 1548.527 | 1550.947 | 1551.523 | 53.08544 | 55.72394 | 60.6579 |
| 1234 | pos_619 | Ethyl 3-hexenoate | 68.86621 | 70.22811 | 71.03874 | 68.21201 | 69.63776 | 70.43419 | 70.71323 | 72.36051 | 79.04897 | 77.77954 | 84.65188 | 91.04056 |
| 1235 | pos_620 | Galactonic acid | 18.15121 | 19.22197 | 19.49899 | 19.50546 | 21.24869 | 23.35341 | 22.27403 | 22.31899 | 23.61305 | 14.72722 | 15.07909 | 16.34091 |
| 1236 | pos_621 | GDP-4-dehydro-6-deoxy-L-mannose | 102.7339 | 103.2075 | 111.8946 | 32.79411 | 36.33844 | 38.14018 | 52.93618 | 54.7902 | 60.06487 | 37.11904 | 38.96405 | 39.00647 |
| 1237 | pos_622 | Inosinic acid | 289.9583 | 308.7964 | 318.9431 | 7.548839 | 7.78898 | 7.82724 | 20.58252 | 21.70083 | 21.85508 | 6.47362 | 6.475949 | 6.661527 |
| 1238 | pos_623 | O-Phospho-4-hydroxy-L-threonine | 39.74974 | 42.62829 | 44.54083 | 8.348609 | 8.789766 | 9.561679 | 8.954814 | 9.258676 | 9.583642 | 0 | 0 | 0 |
| 1239 | pos_624 | 3-aminopropylphosphonate | 53.37403 | 59.02361 | 62.04623 | 61.54863 | 67.97757 | 71.58676 | 59.43703 | 59.71816 | 63.13794 | 62.76377 | 66.22429 | 71.05257 |
| 1240 | pos_625 | C16 Sphinganine | 1303.459 | 1440.534 | 1580.679 | 1405.028 | 1465.278 | 1471.348 | 1119.875 | 1180.643 | 1271.091 | 1485.158 | 1506.662 | 1630.698 |
| 1241 | pos_626 | 16-hydroxy hexadecanoic acid | 5274.441 | 5387.413 | 5509.846 | 5332.349 | 5513.196 | 5789.479 | 4112.842 | 4491.295 | 4660.662 | 5303.579 | 5631.115 | 6049.164 |
| 1242 | pos_627 | Apiin | 139.8841 | 149.9582 | 162.6956 | 63.86355 | 68.81421 | 74.95645 | 52.84473 | 57.44643 | 62.84654 | 39.84476 | 43.14619 | 46.37919 |

Continued Table S2

| 1243 | pos_628 | Astragalin | 1140.602 | 1195.235 | 1274.164 | 0.051273 | 0.051682 | 0.052026 | 0 | 0 | 0 | 0 | 0 | 0 |
| --- | --- | --- | --- | --- | --- | --- | --- | --- | --- | --- | --- | --- | --- | --- |
| 1244 | pos_629 | N-(14-Methylhexadecanoyl)pyrrolidine | 833.9764 | 888.3859 | 902.9719 | 1241.245 | 1324.245 | 1383.584 | 1130.478 | 1207.118 | 1299.914 | 1111.633 | 1146.421 | 1166.85 |
| 1245 | pos_630 | Apigenin 7-glucoside | 6047.171 | 6505.887 | 6588.708 | 8213.983 | 8830.285 | 9438.728 | 4645.988 | 4807.492 | 5205.331 | 5105.322 | 5557.901 | 5823.334 |
| 1246 | pos_631 | 13Z-Docosenamide | 378.4684 | 405.308 | 417.5326 | 296.5832 | 313.5514 | 316.0525 | 199.0079 | 219.3998 | 240.1869 | 454.5125 | 485.5698 | 490.1661 |
| 1247 | pos_632 | Oleandrin | 3780.7 | 4192.238 | 4552.073 | 4464.875 | 4642.277 | 4750.749 | 4062.074 | 4431.995 | 4593.863 | 5316.578 | 5678.357 | 6218.078 |
| 1248 | pos_633 | Shikimate | 6918.033 | 7266.024 | 7816.97 | 340.7403 | 355.6334 | 368.3821 | 591.9924 | 608.8617 | 664.122 | 197.8811 | 202.459 | 207.7392 |
| 1249 | pos_634 | Prunitrin | 69.56178 | 72.79599 | 78.01318 | 34.15972 | 36.23963 | 39.16918 | 10.7051 | 11.84361 | 12.0586 | 14.33204 | 15.31255 | 15.33163 |
| 1250 | pos_635 | Quercitrin | 522.8545 | 533.5391 | 558.5076 | 138.0903 | 150.6372 | 153.0981 | 32.73802 | 36.1679 | 38.12374 | 9.257164 | 10.20648 | 10.48159 |
| 1251 | pos_636 | (+)-Abscisic acid | 22.45153 | 23.98526 | 26.08393 | 19.00794 | 20.43697 | 22.25713 | 128.0877 | 136.5807 | 149.9427 | 15.83577 | 16.36974 | 17.87576 |
| 1252 | pos_637 | 3-Hydroxy-3-methylglutaric acid | 104.8137 | 109.1719 | 116.6064 | 75.06596 | 75.51238 | 76.04442 | 61.70725 | 64.09267 | 66.25884 | 75.69786 | 78.88835 | 83.26867 |
| 1253 | pos_638 | Epigallocatechin gallate | 168.8616 | 176.6338 | 190.7922 | 15.05021 | 16.39679 | 17.54729 | 145.7928 | 159.752 | 167.86 | 0 | 0 | 0 |
| 1254 | pos_639 | Kaempferol 3-O-rutinoside | 243.5118 | 253.3063 | 265.5234 | 1700.075 | 1774.645 | 1862.022 | 2142.977 | 2209.936 | 2390.892 | 1302.023 | 1410.724 | 1551.69 |
| 1255 | pos_640 | Amrinone | 26042.33 | 26384.3 | 27460.68 | 11744.58 | 11937.94 | 12976.22 | 9053.372 | 9407.371 | 10120.27 | 10110.72 | 10218.94 | 10377.97 |
| 1256 | pos_641 | Oxyphencyclimine | 1199.882 | 1283.386 | 1386.901 | 1251.999 | 1357.565 | 1462.534 | 1348.826 | 1416.968 | 1486.494 | 1183.927 | 1312.798 | 1342.433 |
| 1257 | pos_642 | Undecanoic Acid | 3696.04 | 3723.067 | 3742.583 | 3489.45 | 3772.724 | 3881.156 | 3100.991 | 3361.668 | 3659.72 | 4138.441 | 4492.951 | 4845.428 |
| 1258 | pos_643 | Desacetylvindoline | 77045.95 | 85079.49 | 90956.47 | 103631.7 | 106937.5 | 109689.1 | 80251.03 | 88925.06 | 92444.48 | 101027.7 | 111821.8 | 112454.2 |
| 1259 | pos_644 | Aspartyl-Proline | 52.33283 | 56.86591 | 59.446 | 53.53516 | 55.77889 | 58.7966 | 46.28595 | 46.45847 | 48.17604 | 57.32953 | 62.89824 | 66.38588 |
| 1260 | pos_645 | (-)-Epicatechin | 12904.23 | 13552.01 | 14774.58 | 100.6814 | 101.0793 | 108.5848 | 139.4009 | 142.1493 | 146.873 | 98.46935 | 102.1238 | 108.0496 |
| 1261 | pos_646 | .alpha.-Estradiol | 472.4509 | 491.5436 | 506.3739 | 442.1116 | 463.1227 | 497.4752 | 10691.75 | 11176.97 | 12037.43 | 465.1052 | 477.3483 | 515.8689 |
| 1262 | pos_647 | Hyperoside | 423.9606 | 439.259 | 477.1989 | 5587.841 | 5680.037 | 5751.29 | 842.5099 | 871.3996 | 949.4545 | 4726.015 | 4799.67 | 5202.213 |
| 1263 | pos_648 | Erucic acid | 4745.259 | 5248.208 | 5338.04 | 5533.026 | 5564.596 | 6080.964 | 4694.5 | 5001.557 | 5030.179 | 7041.241 | 7115.929 | 7516.038 |
| 1264 | pos_649 | Threoninyl-Methionine | 2811.085 | 2936.775 | 3158.376 | 2946.445 | 3020.81 | 3107.708 | 2642.282 | 2898.914 | 3107.362 | 2664.032 | 2856.112 | 2957.333 |
| 1265 | pos_650 | Glycyl-Arginine | 127.1394 | 141.1144 | 141.959 | 138.8972 | 151.8642 | 162.251 | 124.218 | 125.1819 | 133.6381 | 150.7443 | 162.3841 | 167.6176 |
| 1266 | pos_651 | Serinyl-Lysine | 265.5929 | 275.8216 | 289.0026 | 48.8373 | 53.06653 | 54.97732 | 18.04534 | 19.25514 | 20.96443 | 33.38909 | 35.20186 | 36.69377 |
| 1267 | pos_652 | 6''-O-Malonyldaidzin | 69.1723 | 76.10246 | 78.61354 | 121.9765 | 133.7208 | 144.6717 | 118.3544 | 121.9841 | 124.5274 | 88.02877 | 92.29853 | 99.56198 |
| 1268 | pos_653 | Apigenin 7-O-neohesperidoside | 224.1566 | 244.6767 | 251.4769 | 115.951 | 127.4669 | 132.1084 | 140.2743 | 145.2408 | 153.025 | 116.6991 | 127.4151 | 139.1367 |

Continued Table S2

| 1269 | pos_654 | Arginyl-Valine | 530.1287 | 575.0617 | 626.059 | 597.9156 | 599.6499 | 615.749 | 576.6788 | 627.4746 | 652.8442 | 527.2596 | 529.3811 | 551.7122 |
| --- | --- | --- | --- | --- | --- | --- | --- | --- | --- | --- | --- | --- | --- | --- |
| 1270 | pos_655 | Ergothioneine | 32.07245 | 33.21231 | 35.81668 | 36.06491 | 36.40281 | 38.90643 | 35.27508 | 36.4466 | 36.70662 | 44.23243 | 46.66868 | 48.69061 |
| 1271 | pos_656 | Atenolol | 1963.858 | 2012.236 | 2029.021 | 2157.205 | 2190.405 | 2322.634 | 2125.409 | 2214.875 | 2268.071 | 2396.166 | 2606.211 | 2669.051 |
| 1272 | pos_657 | Methionyl-Glutamate | 266.9081 | 268.1516 | 276.9612 | 107.2261 | 107.7046 | 110.1678 | 157.6768 | 157.9067 | 163.5404 | 145.7603 | 152.8321 | 162.8675 |
| 1273 | pos_658 | Gemcitabine | 417.7227 | 419.7398 | 452.521 | 7.048956 | 7.498265 | 7.808683 | 130.2567 | 141.0549 | 149.9002 | 0.028627 | 0.03022 | 0.032023 |
| 1274 | pos_659 | 2'-Deoxycytidine 5'-monophosphate (dCMP) | 90.2126 | 94.53014 | 95.85499 | 0 | 0 | 0 | 0 | 0 | 0 | 0 | 0 | 0 |
| 1275 | pos_660 | Exalamide | 998.6112 | 1059.102 | 1089.92 | 1100.983 | 1151.634 | 1168.928 | 985.6305 | 1073.243 | 1106.109 | 986.4246 | 1084.174 | 1085.768 |
| 1276 | pos_661 | Embelin | 650.8534 | 719.3345 | 773.7477 | 823.1481 | 908.5972 | 991.7259 | 906.7946 | 921.7332 | 995.8542 | 806.7128 | 852.8435 | 887.0831 |
| 1277 | pos_662 | Mimosine | 854.1404 | 923.7998 | 1012.282 | 264.3218 | 267.5476 | 274.0202 | 311.6495 | 338.7292 | 364.1618 | 169.7368 | 174.387 | 176.0368 |
| 1278 | pos_663 | Fisetinidol | 1617.436 | 1748.58 | 1841.333 | 858.9151 | 922.5958 | 948.6463 | 1773.252 | 1929.183 | 1946.676 | 318.6087 | 336.7244 | 337.7529 |
| 1279 | pos_664 | Loganic Acid | 96.01096 | 98.17331 | 100.73 | 302.956 | 307.0234 | 324.7988 | 313.6023 | 319.525 | 339.5172 | 269.6396 | 279.7885 | 293.1088 |
| 1280 | pos_665 | 6-O-Acetylglycitin | 269.1507 | 295.2909 | 300.1033 | 113.5831 | 124.8017 | 126.8053 | 87.96563 | 94.62124 | 98.24937 | 105.5354 | 107.906 | 113.5024 |
| 1281 | pos_666 | Naringenin-7-O-Glucoside | 136.7052 | 150.6461 | 157.9066 | 27.06414 | 28.46618 | 29.55688 | 102.695 | 105.7775 | 112.9844 | 135.1453 | 137.7205 | 142.8386 |
| 1282 | pos_667 | Amarogentin | 4109.932 | 4290.423 | 4539.549 | 486.6828 | 494.0077 | 536.0753 | 2127.317 | 2280.674 | 2380.391 | 291.3645 | 302.8626 | 317.9999 |
| 1283 | pos_668 | Stavudine | 52.66439 | 53.51816 | 55.46436 | 0.090977 | 0.10105 | 0.103784 | 0 | 0 | 0 | 0 | 0 | 0 |
| 1284 | pos_669 | p-Cresol | 367.7328 | 371.7893 | 384.0393 | 53.02087 | 58.65339 | 59.83961 | 64.16064 | 65.92432 | 68.66757 | 39.69187 | 42.36634 | 44.69154 |
| 1285 | pos_670 | Arginyl-Glutamine | 249.2124 | 275.4996 | 281.7812 | 297.2212 | 324.561 | 356.0025 | 293.8796 | 319.5377 | 348.2925 | 291.4537 | 318.623 | 329.3314 |
| 1286 | pos_671 | Procyanidin A1 | 415.7453 | 435.3738 | 477.8093 | 1132.643 | 1234.912 | 1306.863 | 1782.212 | 1782.527 | 1943.588 | 186.484 | 187.6257 | 187.7559 |
| 1287 | pos_672 | Triamcinolone Diacetate | 87.99536 | 92.85954 | 97.90436 | 119.4196 | 132.0185 | 142.5433 | 110.0362 | 115.1454 | 121.4396 | 205.4211 | 206.5492 | 207.1352 |
| 1288 | pos_673 | Threoninyl-Arginine | 56.78715 | 58.0344 | 63.60746 | 59.1046 | 61.97412 | 62.64653 | 58.95986 | 59.42514 | 62.22081 | 64.11422 | 65.90453 | 71.0912 |
| 1289 | pos_674 | 6''-O-Acetylglycitin | 432.7502 | 478.6787 | 488.1941 | 52.51038 | 54.34118 | 55.31897 | 114.3865 | 115.609 | 121.3952 | 17.38263 | 18.08852 | 18.22888 |
| 1290 | pos_675 | N-Methyl-1-deoxynojirimycin | 30.02582 | 31.61954 | 31.67786 | 57.17443 | 61.8959 | 66.78063 | 44.58027 | 45.89085 | 48.70696 | 61.34316 | 66.45925 | 69.91446 |
| 1291 | pos_676 | 6''-O-Acetylgenistin | 982.614 | 1056.05 | 1132.63 | 1042.695 | 1093.27 | 1100.223 | 948.8056 | 1037.521 | 1137.833 | 1156.994 | 1230.985 | 1308.282 |
| 1292 | pos_677 | Pristanic acid | 1159.337 | 1259.109 | 1296.393 | 1202.488 | 1300.703 | 1360.166 | 1174.77 | 1288.343 | 1292.546 | 1178.597 | 1211.525 | 1217.539 |
| 1293 | pos_678 | Oleamide | 177.7908 | 179.5657 | 196.2744 | 180.2742 | 193.5574 | 195.6685 | 175.2787 | 186.3933 | 194.8379 | 173.3002 | 177.0584 | 178.2698 |
| 1294 | pos_679 | Glutamyl-Methionine | 502.2113 | 515.9521 | 554.4459 | 631.6675 | 680.0081 | 695.1888 | 524.2557 | 527.8598 | 546.1581 | 638.7182 | 683.1425 | 711.2174 |

Continued Table S2

| 1295 | pos_680 | Methyl Heptadecanoic acid | 125.3713 | 136.9066 | 137.7902 | 127.1087 | 137.9509 | 142.5555 | 129.3051 | 138.4531 | 146.0271 | 125.1944 | 127.9702 | 129.4264 |
| --- | --- | --- | --- | --- | --- | --- | --- | --- | --- | --- | --- | --- | --- | --- |
| 1296 | pos_681 | Betaine-1 | 3654.416 | 3667.18 | 3802.219 | 3466.052 | 3699.463 | 4058.765 | 3263.962 | 3602.643 | 3620.092 | 3557.919 | 3780.95 | 3993.877 |
| 1297 | pos_682 | 6''-O-Malonylglycitin | 122.1038 | 135.1213 | 143.2578 | 26.65305 | 28.0468 | 28.93814 | 75.94704 | 81.10183 | 84.71647 | 25.49734 | 27.41784 | 29.02602 |
| 1298 | pos_683 | Streptozocin | 142.6698 | 146.6119 | 159.7375 | 106.2193 | 111.0056 | 113.4698 | 122.9758 | 123.7379 | 128.1685 | 107.7274 | 108.0674 | 109.8714 |
| 1299 | pos_684 | alpha-hydroxy myristic acid | 272.0782 | 301.2627 | 324.0332 | 389.677 | 399.8276 | 403.1057 | 311.1242 | 312.0885 | 317.8813 | 290.7871 | 321.8137 | 346.1748 |
| 1300 | pos_685 | PGD2 | 88.54558 | 92.31938 | 95.84938 | 94.45768 | 104.0184 | 109.424 | 101.5016 | 106.107 | 110.2278 | 90.30218 | 98.18241 | 104.1216 |
| 1301 | pos_686 | Tryptophyl-Lysine | 673.5868 | 734.9011 | 750.4356 | 14.60899 | 16.16677 | 17.68854 | 132.5108 | 138.0814 | 151.7674 | 28.30835 | 29.35793 | 31.89656 |
| 1302 | pos_687 | Lamivudine | 43.80058 | 46.41368 | 50.23392 | 0 | 0 | 0 | 0 | 0 | 0 | 0 | 0 | 0 |
| 1303 | pos_688 | Isoleucyl-Asparagine | 429.2363 | 433.4401 | 462.3204 | 56.427 | 57.96702 | 61.67052 | 52.1816 | 56.35957 | 57.6947 | 122.3967 | 129.4062 | 131.3785 |
| 1304 | pos_689 | P-Fluorophenylalanine | 125.3906 | 136.7163 | 144.7375 | 124.5028 | 128.7926 | 131.975 | 116.6444 | 125.2318 | 129.0482 | 128.067 | 132.5672 | 137.6201 |
| 1305 | pos_690 | .beta.-Cryptoxanthin | 79053.49 | 80972.87 | 85794.14 | 76457.99 | 84832.75 | 89784.56 | 76547.76 | 82200.1 | 90236.6 | 75539.42 | 82067.46 | 88070.08 |
| 1306 | pos_691 | Dodecanoic acid | 2902.657 | 2990.777 | 3192.418 | 2968.685 | 3106.971 | 3398.461 | 2876.136 | 3171.545 | 3370.106 | 4179.538 | 4430.081 | 4539.624 |
| 1307 | pos_692 | 2'-O-methylinosine | 41.35449 | 45.42805 | 47.79815 | 121.0892 | 123.7963 | 127.8025 | 267.2212 | 273.7896 | 278.5181 | 129.6978 | 142.3085 | 152.9688 |
| 1308 | pos_693 | L-leucyl-L-proline | 35.81782 | 38.29392 | 38.42324 | 37.63612 | 37.80151 | 39.99955 | 37.9931 | 40.60215 | 44.25339 | 34.36569 | 36.10946 | 39.46301 |
| 1309 | pos_694 | (-)-Catechin 3-O-gallate | 154.2131 | 162.0924 | 173.8908 | 6.677346 | 7.017688 | 7.495657 | 52.40539 | 57.12305 | 60.15094 | 1.615953 | 1.669467 | 1.708987 |
| 1310 | pos_695 | Rutin | 548.9133 | 553.0833 | 595.6281 | 1.777905 | 1.881906 | 1.99211 | 13.05982 | 13.96389 | 15.0289 | 4.931989 | 5.065566 | 5.570584 |
| 1311 | pos_696 | 12-Oxo-2,3-dinor-10,15-phytodienoic acid | 350.1885 | 357.4485 | 371.8899 | 377.8672 | 416.7692 | 426.9581 | 331.8349 | 361.8072 | 379.9422 | 345.3802 | 347.9484 | 381.555 |
| 1312 | pos_697 | Palatinose | 31.32579 | 31.97936 | 34.78872 | 16.10681 | 17.40971 | 17.43827 | 31.48282 | 31.69198 | 33.73681 | 25.82748 | 27.93405 | 28.85899 |
| 1313 | pos_698 | Muramic acid | 1.473322 | 1.590271 | 1.610788 | 0.071578 | 0.076332 | 0.076454 | 42.98704 | 45.68348 | 48.40341 | 0 | 0 | 0 |
| 1314 | pos_699 | Illudin M | 807.1826 | 813.8478 | 875.7751 | 898.817 | 948.5616 | 963.0319 | 958.1142 | 958.2589 | 1048.511 | 1014.019 | 1018.89 | 1105.581 |
| 1315 | pos_700 | Phenylalanyl-Tryptophan | 0 | 0 | 0 | 1890.956 | 2056.555 | 2184.766 | 400.4303 | 418.0256 | 459.2214 | 490.3208 | 519.5282 | 568.8186 |
| 1316 | pos_701 | 10-Hydroxycamtothecin | 181.4645 | 190.3922 | 203.64 | 58.38592 | 59.20075 | 59.49311 | 48.64104 | 53.19597 | 54.44137 | 50.61206 | 55.01112 | 56.82203 |
| 1317 | pos_702 | Estriol 16.alpha.-(.beta.-D-glucuronide) | 143.3293 | 145.5122 | 157.63 | 173.6698 | 178.5978 | 192.6513 | 175.4552 | 193.2221 | 195.8517 | 194.7691 | 198.9616 | 211.224 |
| 1318 | pos_703 | Procyanidin B2 | 194.6856 | 198.634 | 213.2536 | 408.1569 | 445.3279 | 456.6119 | 138.7191 | 138.7416 | 152.407 | 290.5798 | 299.128 | 328.0233 |
| 1319 | pos_704 | APGPR Enterostatin | 103.9246 | 112.8905 | 117.5499 | 113.9731 | 121.5964 | 121.6777 | 116.5384 | 118.8126 | 130.4773 | 127.7533 | 134.5656 | 137.6225 |
| 1320 | pos_705 | Ginkgetin | 109.2978 | 118.4329 | 122.8261 | 1349.45 | 1406.225 | 1438.98 | 1453.994 | 1468.247 | 1592.394 | 726.1997 | 736.6687 | 762.1042 |

Continued Table S2

| 1321 | pos_706 | Alanyl-Histidine | 32.24916 | 32.85689 | 34.67619 | 38.31184 | 38.93623 | 42.46866 | 39.21492 | 41.34619 | 45.39458 | 42.25974 | 44.41914 | 45.36193 |
| --- | --- | --- | --- | --- | --- | --- | --- | --- | --- | --- | --- | --- | --- | --- |
| 1322 | pos_707 | 1-Octadecanol (Drug) | 588.2743 | 650.693 | 703.6437 | 673.7353 | 716.9908 | 733.4157 | 728.3927 | 775.2166 | 827.8494 | 850.1743 | 914.2976 | 941.6862 |
| 1323 | pos_708 | Mitomycin | 199.7807 | 208.2362 | 211.7869 | 179.148 | 192.7851 | 208.5099 | 191.4358 | 191.7848 | 195.8404 | 181.9134 | 196.9223 | 202.7456 |
| 1324 | pos_709 | Cytarabine | 501.7317 | 517.1927 | 549.8528 | 27.62314 | 29.25038 | 31.66929 | 81.50226 | 84.82161 | 87.49926 | 12.03564 | 12.42128 | 13.29995 |
| 1325 | pos_710 | Xanthosine | 337.0199 | 366.7708 | 402.2091 | 148.5822 | 161.1842 | 172.5981 | 348.2363 | 386.1401 | 412.0275 | 117.5792 | 124.4309 | 135.7803 |
| 1326 | pos_711 | (-)-Naringenin | 80.66084 | 88.09196 | 89.75129 | 0 | 0 | 0 | 0 | 0 | 0 | 0 | 0 | 0 |
| 1327 | pos_712 | Hygromycin B | 0 | 0 | 0 | 0 | 0 | 0 | 981.9456 | 1005.139 | 1103.972 | 0 | 0 | 0 |
| 1328 | pos_713 | Methionyl-Histidine | 59.08539 | 62.62579 | 65.20534 | 27.62862 | 30.14152 | 32.73384 | 45.03834 | 47.13813 | 47.19186 | 12.80229 | 13.1639 | 13.94697 |
| 1329 | pos_714 | Sulindac | 56.15747 | 60.68315 | 62.23081 | 32.89071 | 35.83318 | 37.17135 | 32.34766 | 33.25436 | 35.57665 | 40.22828 | 42.28243 | 45.24336 |
| 1330 | pos_715 | deoxyguanosine 5'-monophosphate (dGMP) | 42.88688 | 44.42805 | 44.81946 | 75.5474 | 78.18759 | 78.55545 | 96.09658 | 97.94476 | 102.1343 | 61.09097 | 65.91082 | 70.91295 |
| 1331 | pos_716 | 3-Hydroxy-4-methoxybenzoic acid | 5956.865 | 6077.778 | 6600.838 | 343.4005 | 353.4568 | 373.3207 | 667.9683 | 687.6598 | 726.9354 | 298.812 | 320.075 | 324.3357 |
| 1332 | pos_717 | S-Allyl-L-cysteine | 222.8754 | 231.4516 | 232.2687 | 34.23994 | 34.3993 | 37.20127 | 51.10259 | 56.10164 | 59.3382 | 32.71135 | 34.04124 | 35.81702 |
| 1333 | pos_718 | Diosmin | 96.50729 | 99.15554 | 105.4098 | 42.51844 | 43.428 | 47.50378 | 57.83621 | 62.0149 | 67.27866 | 34.41652 | 35.19972 | 36.8956 |
| 1334 | pos_719 | 9(S)-HOTrE | 0 | 0 | 0 | 158.0843 | 166.771 | 176.7268 | 0 | 0 | 0 | 99.02853 | 105.2993 | 114.9247 |
| 1335 | pos_720 | L-phenylalanyl-L-proline | 3.138766 | 3.286964 | 3.38504 | 58.8494 | 59.93526 | 64.30535 | 2.089596 | 2.261853 | 2.442829 | 204.9253 | 206.8502 | 214.6365 |
| 1336 | pos_721 | 3-Amino-3-(4-hydroxyphenyl)propanoate | 38.39724 | 40.63227 | 41.56454 | 25.5455 | 25.57781 | 27.46674 | 26.64473 | 28.03713 | 30.732 | 23.63233 | 25.93004 | 26.79448 |
| 1337 | pos_722 | S-Nitroso-L-glutathione | 70.76664 | 75.1426 | 78.67199 | 73.1984 | 77.99058 | 85.28546 | 122.8 | 135.9149 | 136.073 | 155.3558 | 164.3306 | 169.9892 |
| 1338 | pos_723 | Galactosylsphingosine | 152.4935 | 161.647 | 172.5063 | 136.771 | 151.9395 | 160.187 | 131.2181 | 142.0279 | 152.2847 | 115.7134 | 126.8265 | 133.9211 |
| 1339 | pos_724 | Floxuridine | 5.556072 | 5.849216 | 6.109971 | 2.92961 | 3.037805 | 3.193032 | 5.57404 | 5.662498 | 5.871925 | 2.327597 | 2.501094 | 2.574669 |
| 1340 | pos_725 | beta-Nicotinamide D-ribonucleotide | 2.923566 | 3.020845 | 3.264272 | 14.59838 | 14.69182 | 16.02711 | 27.14066 | 29.96586 | 30.26096 | 16.73439 | 17.0302 | 18.65498 |
| 1341 | pos_726 | 5'-Phosphoribosyl-5-amino-4-imidazolecarboxamide (AICAR) | 24.49137 | 26.1642 | 27.67179 | 109.4569 | 114.8162 | 125.3112 | 285.3385 | 288.2484 | 301.0178 | 98.92349 | 102.7708 | 107.7652 |
| 1342 | pos_727 | Parthenin | 51.59548 | 53.83498 | 55.98059 | 57.05828 | 59.91621 | 61.91048 | 63.49199 | 64.02229 | 64.41988 | 61.30464 | 61.34917 | 62.22357 |
| 1343 | pos_728 | Methaqualone | 12.32647 | 13.67339 | 14.35435 | 24.33586 | 26.14808 | 27.74282 | 827.5746 | 841.0675 | 864.1543 | 161.4623 | 171.4493 | 184.5902 |
| 1344 | pos_729 | Deoxycoformycin | 203.2153 | 224.3673 | 236.1166 | 171.2579 | 182.0868 | 191.8443 | 181.6434 | 196.5476 | 196.6914 | 172.1113 | 189.3223 | 196.4361 |
| 1345 | pos_730 | Vindoline | 75.09992 | 79.74008 | 86.5894 | 103.8765 | 105.4351 | 114.4589 | 85.47722 | 88.63161 | 90.96667 | 125.0957 | 128.31 | 136.2029 |

Continued Table S2

| 1346 | pos_731 | Ginkgolide J | 11.97393 | 12.74553 | 13.10167 | 268.8469 | 285.8594 | 289.0119 | 78.80761 | 79.28084 | 84.42205 | 319.2474 | 332.4541 | 359.9944 |
| --- | --- | --- | --- | --- | --- | --- | --- | --- | --- | --- | --- | --- | --- | --- |
| 1347 | pos_732 | Helenalin | 227.0606 | 244.8494 | 263.2368 | 258.2146 | 282.053 | 296.665 | 296.0806 | 317.5007 | 319.5421 | 352.1452 | 376.3137 | 407.0769 |
| 1348 | pos_733 | Phenylpyruvate | 941.1199 | 998.5172 | 1004.876 | 1890.379 | 2033.121 | 2042.145 | 1220.14 | 1306.502 | 1371.291 | 7438.795 | 7457.726 | 8052.353 |
| 1349 | pos_734 | Ginkgolide C | 594.9731 | 627.13 | 677.6911 | 146.5791 | 161.7876 | 168.7117 | 99.24396 | 103.3889 | 111.2459 | 127.8022 | 133.4073 | 140.2497 |
| 1350 | pos_735 | Ribothymidine | 74.5568 | 75.71519 | 79.73861 | 143.1228 | 144.1431 | 144.651 | 250.8584 | 265.2159 | 270.297 | 119.5286 | 124.6996 | 129.2004 |
| 1351 | pos_736 | Oleanolic acid | 505.7505 | 526.7165 | 569.7253 | 523.1627 | 540.1534 | 546.4116 | 579.7881 | 596.5977 | 624.8601 | 525.0011 | 529.0914 | 559.455 |
| 1352 | pos_737 | Tryptophyl-Glutamine | 18.38151 | 20.31919 | 20.42468 | 20.13472 | 21.46708 | 22.75296 | 1022.066 | 1081.642 | 1140.651 | 16.57497 | 16.92472 | 18.30897 |
| 1353 | pos_738 | Piracetam | 352.1757 | 356.8836 | 384.3466 | 334.7136 | 345.1865 | 375.3855 | 338.1266 | 374.8661 | 390.1598 | 319.5984 | 345.1675 | 378.788 |
| 1354 | pos_739 | Azacitidine | 100.3726 | 107.2862 | 110.6649 | 3.153117 | 3.496252 | 3.810928 | 3.421172 | 3.616829 | 3.976019 | 2.720048 | 2.739047 | 2.818494 |
| 1355 | pos_740 | Betulafolienetriol | 457.4057 | 493.6802 | 518.961 | 521.9251 | 524.9704 | 530.4141 | 636.515 | 652.5771 | 695.7173 | 475.7535 | 516.2853 | 521.5528 |
| 1356 | pos_741 | Mestranol | 9128.125 | 10050.12 | 10665.49 | 9681.505 | 10208.6 | 10612.99 | 9610.922 | 9973.443 | 10030.21 | 9502.101 | 10335.34 | 11174.51 |
| 1357 | pos_742 | 3,4-Dihydroxymandelic acid | 72.9317 | 80.43157 | 86.92286 | 55.33869 | 61.46012 | 65.02177 | 57.64531 | 61.89982 | 63.21893 | 58.73572 | 59.06025 | 62.09351 |
| 1358 | pos_743 | Tryptophanol | 240.043 | 252.7069 | 266.3603 | 197.3494 | 198.2281 | 201.0423 | 274.1312 | 278.3241 | 285.1498 | 186.9516 | 206.4847 | 220.1391 |
| 1359 | pos_744 | Neolinustatin | 90.32594 | 95.31726 | 102.5253 | 0 | 0 | 0 | 0 | 0 | 0 | 1.175703 | 1.188937 | 1.200693 |
| 1360 | pos_745 | L-erythro-tetrahydrobiopterin | 76.28932 | 80.47445 | 88.17254 | 80.82866 | 83.6154 | 91.6521 | 71.90649 | 72.05435 | 76.31842 | 66.98121 | 69.25295 | 70.35492 |
| 1361 | pos_746 | Asparaginyl-Asparagine | 50.05999 | 51.89374 | 56.16629 | 78.9758 | 81.12107 | 88.33669 | 122.6716 | 133.7774 | 139.726 | 95.1309 | 99.37541 | 102.7284 |
| 1362 | pos_747 | Terfenadine | 8.113278 | 8.988721 | 9.202181 | 2.52499 | 2.71569 | 2.763672 | 139.7892 | 148.366 | 156.8902 | 1.391928 | 1.498101 | 1.49937 |
| 1363 | pos_748 | Chlorogenate | 1283.759 | 1375.901 | 1498.736 | 50.78692 | 52.30001 | 57.44699 | 69.87671 | 74.96031 | 82.23922 | 39.3762 | 40.83697 | 44.31237 |
| 1364 | pos_749 | Ginkgolide B | 28.18444 | 30.28043 | 30.43419 | 35.71732 | 37.82383 | 40.50951 | 2268.564 | 2381.381 | 2414.474 | 25.31695 | 27.22068 | 28.00719 |
| 1365 | pos_750 | Carbamazepine | 110.6958 | 113.2411 | 120.8998 | 5.640708 | 6.052146 | 6.278259 | 6.390506 | 7.041271 | 7.595799 | 2.188664 | 2.35232 | 2.374896 |
| 1366 | pos_751 | Daidzin | 21.94227 | 23.26885 | 25.36309 | 8.952199 | 9.227068 | 9.337952 | 12.1154 | 13.12404 | 14.42626 | 9.260321 | 10.17943 | 10.37743 |
| 1367 | pos_752 | gamma-L-Glutamyl-L-glutamic acid | 151.198 | 153.1638 | 163.3075 | 206.3684 | 219.8917 | 236.4857 | 331.7069 | 342.3649 | 364.2984 | 199.5251 | 208.6953 | 217.2706 |
| 1368 | pos_753 | Narcissin | 7.220865 | 7.82686 | 8.581877 | 522.3435 | 523.1927 | 556.7562 | 431.2801 | 458.139 | 471.8592 | 489.3865 | 523.0558 | 533.8058 |
| 1369 | pos_754 | Lysyl-Proline | 344.0532 | 354.7982 | 357.7447 | 331.5824 | 367.5396 | 376.9274 | 344.1142 | 363.0383 | 385.569 | 330.1872 | 356.9397 | 376.6668 |
| 1370 | pos_755 | Prednisone | 0 | 0 | 0 | 64.24612 | 69.0019 | 73.04744 | 0 | 0 | 0 | 278.1844 | 286.6016 | 303.3873 |
| 1371 | pos_756 | 3-methylcytidine | 110.801 | 113.0405 | 118.0107 | 112.6147 | 115.6227 | 119.6852 | 104.0352 | 111.0468 | 114.1172 | 104.958 | 106.7816 | 114.2268 |

Continued Table S2

| 1372 | pos_757 | Baicalin | 19.97007 | 19.98791 | 21.70437 | 53.99319 | 59.48772 | 62.80488 | 84.86318 | 85.75246 | 89.91079 | 48.88191 | 48.88928 | 49.24643 |
| --- | --- | --- | --- | --- | --- | --- | --- | --- | --- | --- | --- | --- | --- | --- |
| 1373 | pos_758 | Teniposide | 177.0267 | 186.5637 | 194.1787 | 302.2002 | 330.0875 | 344.8817 | 335.9608 | 337.9038 | 365.9562 | 272.018 | 298.0861 | 314.8082 |
| 1374 | pos_759 | 5'-O-methylthymidine | 93.59029 | 97.73768 | 99.67424 | 87.64359 | 88.67275 | 93.12752 | 83.83108 | 88.57864 | 89.35225 | 76.78981 | 82.6486 | 89.24178 |
| 1375 | pos_760 | 2-Aminoheptanedioic acid | 154001.7 | 162313.4 | 171495.7 | 122991.5 | 130077.2 | 142942.3 | 118039.6 | 123786.5 | 127938 | 120180.1 | 129180.1 | 131088.4 |
| 1376 | pos_761 | Triacetate | 414.2024 | 433.4405 | 444.0182 | 437.3063 | 446.3246 | 459.0004 | 380.0934 | 411.6123 | 437.415 | 379.7002 | 406.1558 | 433.6863 |
| 1377 | pos_762 | Aspartame | 112.1529 | 124.1347 | 124.7703 | 178.0498 | 195.1522 | 207.2893 | 117.165 | 127.4088 | 131.6574 | 315.354 | 318.289 | 325.8859 |
| 1378 | pos_763 | Flavin mononucleotide (FMN) | 383.8163 | 410.3837 | 431.5805 | 45.79219 | 49.4644 | 52.31919 | 36.40111 | 38.65988 | 40.92452 | 47.11228 | 51.87375 | 52.56688 |
| 1379 | pos_764 | Geldanamycin | 0 | 0 | 0 | 363.5065 | 373.027 | 395.1815 | 148.8497 | 158.1128 | 158.3773 | 223.3743 | 230.8985 | 251.6159 |
| 1380 | pos_765 | Glycerol 1-myristate | 99.32296 | 110.0263 | 117.244 | 118.5417 | 120.243 | 125.597 | 106.477 | 117.4643 | 122.408 | 110.4677 | 112.7644 | 119.3354 |

The different fermentation treatments of instant dark tea were the blank control (IDTA), *Aspergillus* *niger* (IDTB), *Aspergillus cristatus* (IDTC), and *Aspergillus* *tubingensis* (IDTD). The metabolites obtained by positive ion mode was named as “pos_n”, and it obtained by negative ion mode was named as “neg_n”.
